# A functional genomic screen in vivo identifies CEACAM5 as a clinically relevant driver of breast cancer metastasis

**DOI:** 10.1038/s41523-018-0062-x

**Published:** 2018-04-30

**Authors:** Emily Powell, Jiansu Shao, Hector M. Picon, Christopher Bristow, Zhongqi Ge, Michael Peoples, Frederick Robinson, Sabrina L. Jeter-Jones, Christopher Schlosberg, Caitlin L. Grzeskowiak, Fei Yang, Yun Wu, Ignacio Wistuba, Stacy L. Moulder, William F. Symmans, Kenneth L. Scott, John R. Edwards, Han Liang, Timothy P. Heffernan, Helen Piwnica-Worms

**Affiliations:** 1 Department of Experimental Radiation Oncology, The University of Texas MD Anderson Cancer Center, Houston, TX 77030 USA; 2 Center for Co-Clinical Trials and Institute for Applied Cancer Science, The University of Texas MD Anderson Cancer Center, Houston, TX 77030 USA; 3 Department of Bioinformatics and Computational Biology, The University of Texas MD Anderson Cancer Center, Houston, TX 77030 USA; 4 Department of Systems Biology, The University of Texas MD Anderson Cancer Center, Houston, TX 77030 USA; 5 Center for Pharmacogenomics, Department of Medicine, Washington University in St. Louis School of Medicine, St. Louis, MO 63110 USA; 6 Department of Molecular and Human Genetics, Baylor College of Medicine, Houston, TX 77030 USA; 7 Department of Translational Molecular Pathology, The University of Texas MD Anderson Cancer Center, Houston, TX 77030 USA; 8 Department of Pathology, The University of Texas MD Anderson Cancer Center, Houston, TX 77030 USA; 9 Department of Breast Medical Oncology, The University of Texas MD Anderson Cancer Center, Houston, TX 77030 USA

## Abstract

Tumor cells disseminate early in tumor development making metastasis-prevention strategies difficult. Identifying proteins that promote the outgrowth of disseminated tumor cells may provide opportunities for novel therapeutic strategies. Despite multiple studies demonstrating that the mesenchymal-to-epithelial transition (MET) is critical for metastatic colonization, key regulators that initiate this transition remain unknown. We serially passaged lung metastases from a primary triple negative breast cancer xenograft to the mammary fat pads of recipient mice to enrich for gene expression changes that drive metastasis. An unbiased transcriptomic signature of potential metastatic drivers was generated, and a high throughput gain-of-function screen was performed in vivo to validate candidates. Carcinoembryonic antigen-related cell adhesion molecule 5 (CEACAM5) was identified as a metastatic driver. CEACAM5 overproduction enriched for an epithelial gene expression pattern and facilitated tumor outgrowth at metastatic sites. Tissues from patients with metastatic breast cancer confirmed elevated levels of CEACAM5 in lung metastases relative to breast tumors, and an inverse correlation between CEACAM5 and the mesenchymal marker vimentin was demonstrated. Thus, CEACAM5 facilitates tumor outgrowth at metastatic sites by promoting MET, warranting its investigation as a therapeutic target and biomarker of aggressiveness in breast cancer.

## Metastasis: Protein implicated in cancer spread to the lungs

A screen for drivers of metastasis has revealed a key protein involved in the spread of breast cancer into lung tissues. A US research team led by Helen Piwnica-Worms from the University of Texas MD Anderson Cancer Center in Houston enriched cells for genes involved in metastasis by engrafting mice with breast tumor biopsies taken from women with metastatic triple negative breast cancer and then metastases of these mice to mammary fat pads of recipient mice. The researchers pinpointed the gene encoding CEACAM5—a protein known to play a role in cell invasion and spread—as a key promoter of the cellular transition associated with metastasis. Tissues samples from patients confirmed that CEACAM5 levels were elevated in metastatic lung tumors relative to primary breast tumors. The protein provides a potential therapeutic target for drug development and candidate biomarker for patient stratification.

## Introduction

Metastatic breast cancer is incurable in most cases.^1^ Triple negative breast cancer (TNBC) is an aggressive subtype of breast cancer that is characterized by high rates of metastasis and poor prognosis.^2,3^ TNBC preferentially metastasizes to visceral organs including lungs.^2^ Metastasis is a multistep process that includes escape from the primary tumor, survival in the blood circulation, extravasation, and tumor outgrowth in the metastatic site.^4^ Successful completion of each of these steps is essential for the formation of macroscopic metastatic disease. The development of novel strategies to prevent or treat metastatic TNBC will rely on defining properties of the tumor cell as well as the metastatic niche that enable dissemination, seeding, and outgrowth of metastatic tumor cells.^5,6^

Previous studies identified gene expression changes in metastatic subpopulations of breast cancer cells,^7–10^ and in some cases these gene expression changes predicted patient survival.^9,11,12^ Unbiased genetic screens are useful for simultaneously identifying and functionalizing metastatic drivers as well as suppressors. In vivo screening approaches including those that utilize shRNAs or CRISPR technology have been useful for identifying genes that suppress metastasis.^13^ However, metastasis suppressors are generally not candidate therapeutic targets. Gain-of-function forward genetic screens in orthotopically-engrafted cancer cell lines have identified a limited number of metastasis drivers.^14,15^ However, successful translation of these collective findings into the clinical setting has been limited.^16^ Furthermore, discordance between in vitro and in vivo screens has been reported, highlighting the importance of in vivo model systems.^17^ Orthotopic patient-derived xenograft (PDX) models are powerful tools for identifying metastatic drivers because the tumors retain much of the genomic heterogeneity of the original patient tumor and require metastatic cells to dynamically regulate gene expression programs as they metastasize in vivo.^18^

Epithelial-to-mesenchymal transition (EMT) and its reverse process mesenchymal-to-epithelial transition (MET) are dynamic developmental programs that contribute to metastasis.^19,20^ Epithelial cells undergoing EMT down regulate expression of cell adhesion molecules, lose adhesive contacts with neighboring cells, and acquire an invasive migratory phenotype. Thus, EMT endows cells with the ability to escape the primary tumor, enter into the hematogeneous and lymphatic circulatory systems, extravasate, and invade the metastatic site. However, cells that have undergone EMT exhibit decreased rates of proliferation, and thus are not well equipped to populate the metastatic site.^19^ Therefore, upregulation of genes that promote MET may endow tumor cells with a selective advantage at the metastatic site. Despite findings from multiple studies that MET is critical for metastatic colonization,^19,21,22^ the key regulators that initiate the transition from the mesenchymal to the epithelial cellular state at the metastatic site remain largely unknown. These regulators may serve as clinical biomarkers for the aggressiveness of metastatic lesions that have already colonized the metastatic site. Indeed, numerous studies have revealed that tumor cells disseminate to metastatic sites early in tumor progression,^23–25^ and thus metastasis-prevention strategies may not be clinically feasible. Therefore, focusing preclinical efforts on characterizing and targeting tumor cells that have already seeded the secondary site may be a promising clinical intervention for patients with metastatic TNBC. Here, a functional genomic screen, performed in vivo using a PDX model derived from the primary breast tumor of a patient with metastatic TNBC, identified CEACAM5 as a metastatic driver, and functional studies demonstrated a role for CEACAM5 in late-stage metastasis.

## Results

### Serial passaging of lung metastases in vivo enriches for metastatic potential

The PDX line WU-BC3 was generated by engrafting breast tumor cells from a patient with metastatic TNBC into the humanized mammary fat pads (MFPs) of NOD/SCID mice mice.^26,27^ We engineered these tumor cells to stably express Click Beetle Red Luciferase (CBR-Luc), mCherry, and an shRNA specific for p53 (shA2) to create the PDX line BC3_A2 (formerly known as BC3-p53KD^28^) (Supplementary Fig. 1a). We demonstrated that tumor cells metastasized from MFPs to physiologically relevant sites including lung, liver, bone, brain, and lymph nodes.^28^ Lung metastases (P0) were isolated from replicate mice, pooled, and engrafted into the MFPs of recipient mice. The resulting lung metastases (P1) were isolated, pooled, and engrafted into the MFPs of additional mice. The lung metastases from these mice (P2) were also collected and pooled (Supplementary Fig. 1b). We performed bioluminescence imaging (BLI) of lungs, bones, and livers at necropsy to quantify the relative magnitude of metastasis at each passage (Supplementary Fig. 1c). Serial passaging enriched for tumor cells with increased capacity to metastasize to all sites (Supplementary Fig. 1d–f, h) and required mice to be euthanized sooner (Supplementary Fig. 1i). By contrast, serial passaging tumors between MFPs did not result in increased lung metastasis (Supplementary Fig. 1g), indicating that acquisition of enhanced metastatic capacity was not simply a consequence of serially passaging tumors in vivo. Furthermore, tumor cells with enhanced metastatic capacity did not exhibit a significant increase in growth properties when isolated from MFPs and placed back into the MFPs of recipient mice (Supplementary Fig. 1j). Taken together, these results demonstrate that serial passaging of tumor cells from lungs to MFPs in this model enriches for metastatic potential but not organ-specific homing.

### Mesenchymal gene expression is reduced in lung metastases relative to MFP tumors

**Fig. 1:**
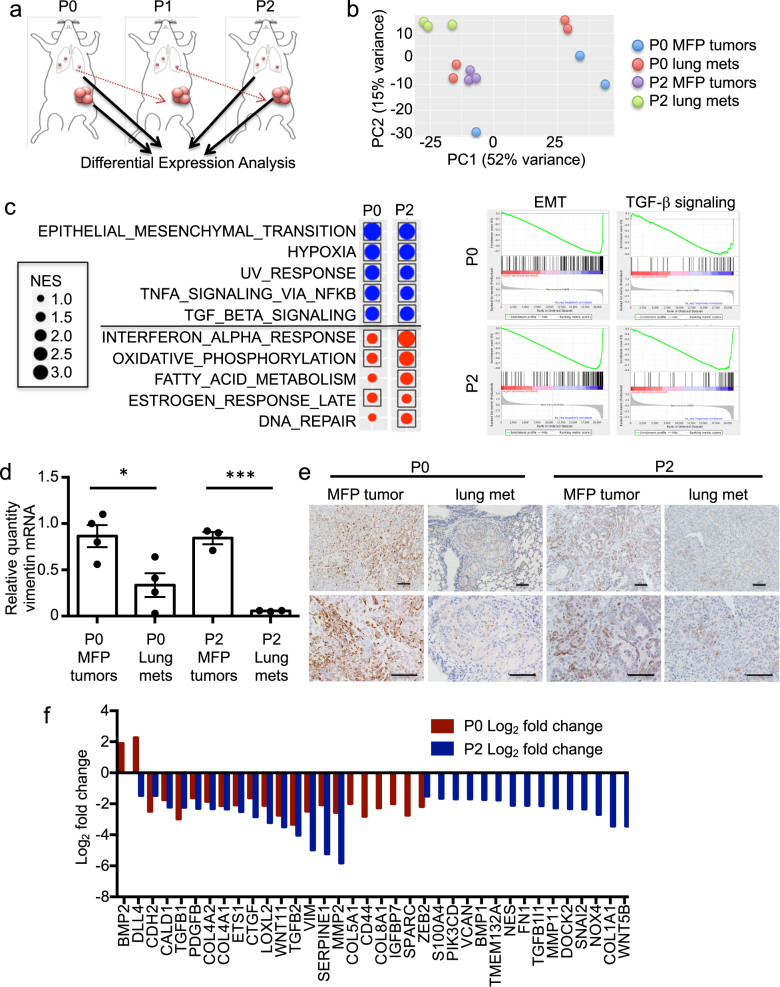
PDX lung metastasis signatures identify genes de-regulated in metastases. **a** Schematic representation of MPF tumors and lungs metastases isolated for RNAseq. **b** Principal component analyses (PCAs) from RNAseq data generated from MFP tumors and lung metastases used in this study. Each data point represents one sample from an individual mouse. **c** GSEA pathway analysis on the enriched lung metastasis signature (P2); the top 5 down (blue)- and up (red)regulated processes are shown. NES, normalized enrichment score. Boxes indicate FDR <0.1 for statistical significance. Enrichment plots for epithelial mesenchymal transition (EMT) and TGF-β signaling for P0 and P2 are shown in the right-hand panel. *p* < 0.001 for EMT and TGF-β signaling at P0 and P2. **d** qRT-PCR analysis of vimentin expression in BC3_A2 MFP tumors and metastatic subpopulations isolated from lung. Paired *t*-tests, *p* = 0.02 for P0 lung, *p* < 0.001 for P2 lung. Each data point represents one mouse. Error bars represent SEM of biological replicates. See also Supplementary Fig. 2. **e** IHC staining was performed on the indicated tissue sections using an antibody recognizing the human-specific form of vimentin. Scale bars indicate 100 μm. **f** Waterfall plot showing expression of EMT genes in P0 and P2 lung metastases compared with corresponding MFP tumors. *p*-values are provided in Supplementary Table 1. Samples from at least three independent mice were included for RNAseq analyses

To identify changes in gene expression patterns that accompany metastasis, we performed RNA sequencing (RNAseq) on lung metastases (P0 and P2) and MFP (P0 and P2) tumors (Fig. 1a and Supplementary Table 1). After subtracting RNAseq reads mapping to the mouse transcriptome, only human transcript reads were used for downstream analyses. Principal component analysis (PCA) revealed decreased discordance among biological replicates with repeated passaging in vivo, as biological replicates of P2 tumor samples clustered more closely with each other than with other subpopulations analyzed (Fig. 1b). Gene Set Enrichment Analysis (GSEA) revealed that EMT was the most significantly downregulated pathway in both P0 and P2 lung metastases relative to MFP tumors (Fig. 1c). TGF-β signaling, which regulates EMT, was also significantly down-regulated in both P0 and P2 lung metastases (Fig. 1c).

Quantitative real time (qRT-PCR) was performed to monitor expression of vimentin, a mesenchymal marker, in MFP tumors and lung metastases throughout serial passaging. Vimentin expression exhibited a dynamic pattern with higher expression in MFP tumors relative to corresponding lung metastases at each passage (Fig. 1d) and to liver metastases at P0 (Supplementary Fig. 2a). Vimentin protein levels recapitulated this dynamic pattern (Fig. 1e). Additional EMT genes were also downregulated in lung metastases relative to corresponding MFP tumors (Fig. 1f). Expression of the mesenchymal marker CDH2 (the gene encoding N-cadherin) followed the same pattern as vimentin (Supplementary Fig. 2b), whereas expression of the epithelial marker CDH1 (the gene encoding E-cadherin) exhibited an inverse trend (Supplementary Fig. 2c). Co-engrafted human stromal fibroblasts were cleared by the time of tumor harvest (Supplementary Fig. 3a–f), ruling out the possibility that they contributed to the expression of mesenchymal markers in MFP tumors. Taken together, these results demonstrate that tumors downregulate expression of mesenchymal genes once established in metastatic sites.

### Determination of library complexity for high throughput gain of function screen

A high throughput gain-of-function (GOF) screen was designed to identify functional drivers of metastasis from our P2 lung metastases signature. HOXA1, a known driver of metastasis,^29^ served as a positive control for screen optimization, and GFP was used as a negative control. BC3_A2 cells were transduced with lentivirus encoding either HOXA1 or GFP, and infected tumor cells were mixed in different ratios (Supplementary Fig. 4a). Mixed populations of tumor cells were then engrafted into the MFPs of recipient mice, and BLI was used to score lung metastases at the time of necropsy (Supplementary Fig. 4b). In a second set of experiments, the metastatic driver ID1^9^ was used in combination with GFP, but in this case mixed populations of tumor cells were injected into the tail vein of recipient mice to model the final stages of metastasis (Supplementary Fig. 4c). A significant increase in photon flux was observed from tumors with a HOXA1: GFP or ID1: GFP ratio of 1:10. These complexity tests demonstrated that a *bona fide* metastasis driver could be detected in our screen if pooled with 9 non-driver open reading frames (ORFs), but a pool of one driver with 19 non-drivers masked our ability to detect the driver. Based on these results we limited our pool size to 12 ORFs.

### High throughput gain-of-function screening in vivo identifies candidate functional drivers of TNBC metastasis

**Fig. 2:**
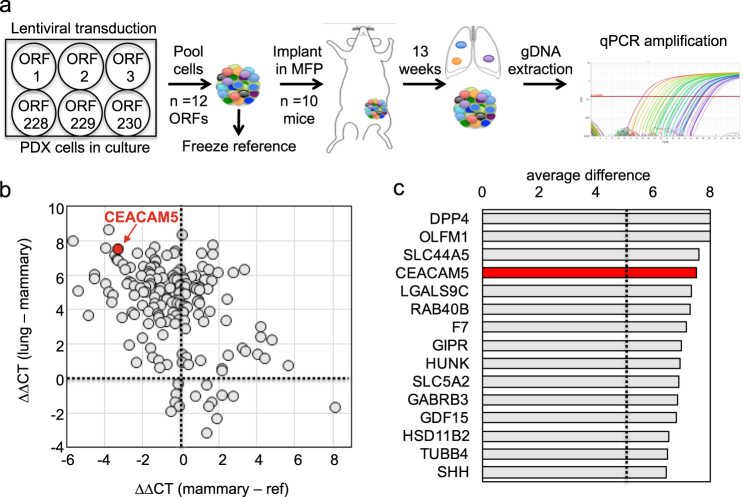
High throughput gain-of-function screening *in vivo* identifies functional drivers of metastasis. **a** Schematic depicting format used for gain-of-function (GOF) screen. See also Supplementary Fig. 5. ORF, open reading frame. Ten mice were implanted in each cohort. **b** Scatter plot showing relative representation of each ORF in MFP tumors following normalization to the reference (*x* axis) and in lungs relative to MFP tumors (*y* axis). The ΔΔCT method was used for quantification of qPCR data. CEACAM5 is shown in red. **c** Genes enriched in lung metastases but not MFP tumors [upper left quadrant of panel (**b**)] were ranked in descending order of enrichment in lung. Dotted line indicates enrichment score cutoff. See also Supplementary Fig. 5 and Supplementary Table 2

To determine which of the upregulated genes from the P2 lung metastasis signature were capable of driving lung metastasis, we designed custom barcoded lentiviral libraries encoding the ORFs of the 230 genes that were most highly upregulated in lung metastases. ORFs were individually expressed in BC3_A2 cells so that each cell line expressed a single ORF, cells were then pooled in groups of 12, and pools were implanted into the MFPs of recipient mice (*n* = 10 mice per pool, Fig. 2a). A control plasmid expressing GFP was included in each pool to gauge intrinsic metastasis in this TNBC model. Each ORF was associated with a unique DNA barcode. A reference pellet of pooled cells was snap frozen to confirm equal representation of each ORF at the time of tumor implantation. Thirteen weeks was chosen as the study endpoint because HOXA1, but not GFP-expressing tumors, metastasized to lung by this time point (Supplementary Fig. 5a). Thirteen weeks post engraftment, lungs were subjected to BLI *ex vivo* (Supplementary Fig. 5a), metastatic lesions were isolated, and genomic DNA (gDNA) was extracted and used as a template for qPCR to amplify unique barcode regions associated with each ORF (Supplementary Fig. 5b). MFP tumors were also isolated and subjected to these analyses (Fig. 2a). The representation of each barcoded ORF in the MFP relative to the reference sample was determined for each pool (Fig. 2b; Supplementary Figs. 5c, d). Representation in the lung was then normalized to enrichment in MFPs vs. the reference (Fig. 2b, c). Metastasis drivers segregated into three groups, including those enriched in both lungs and MFPs (Fig. 2b, upper right quadrant and Supplementary Fig. 5c), those enriched exclusively in MFPs (Fig. 2b, lower right quadrant) and those enriched exclusively in lungs (Fig. 2b, upper left quadrant and Fig. 2c). Cells expressing GFP were enriched in lung metastases in some of the pools and the average enrichment score for GFP in the lung was approximately 5 (Supplementary Fig. 5e). Therefore, we used this as an enrichment score cutoff to define a true hit in our screen and identified 44 genes that were selectively enriched in lung metastases relative to primary tumors. These hits were ranked by enrichment score (Fig. 2c and Supplementary Table 2). Among the top five hits, CEACAM5 was the only one whose expression in primary tumors was found to predict poor survival in breast cancer patients (Supplementary Fig. 6a). Therefore, we further investigated the functional role of CEACAM5 in breast cancer metastasis.

### Confirmation of enhanced CEACAM5 expression in additional metastatic PDX models

**Fig. 3:**
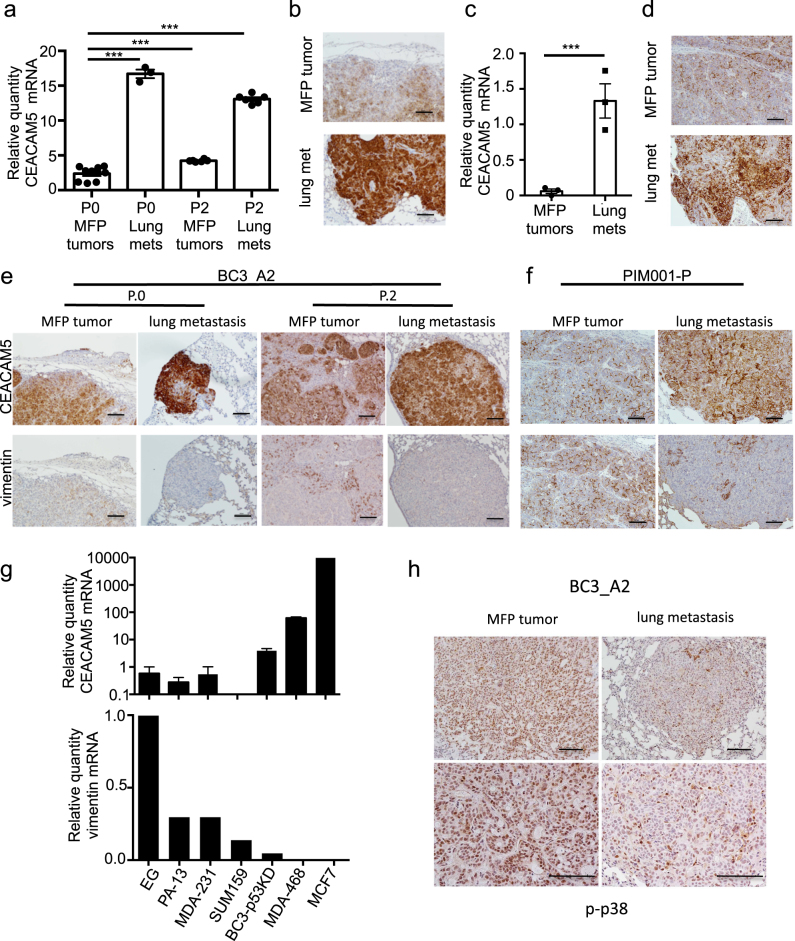
CEACAM5 is upregulated in metastases, and its expression is inversely correlated with that of vimentin in PDX models of TNBC. **a** Expression of CEACAM5 in MFP tumors and metastatic lesions of mice engrafted with BC3_A2 tumors was determined by qRT-PCR. Paired t-tests, *p* < 0.001 for P0 lung, P2 MFP tumor, and P2 lung. Each dot represents an individual mouse. Error bars represent SEM of biological replicates. See also Supplementary Fig. 7. **b** MFP tumors and corresponding metastases from mice engrafted with BC3_A2 tumors were analyzed by IHC using a CEACAM5 antibody. Scale bars indicate 100 μm. **c** Expression of CEACAM5 in MFP tumors and metastatic lesions of mice engrafted with PIM001-P tumors was determined by qRT-PCR. Data are presented on a log scale. Paired *t*-test, *p* < 0.001. Each dot represents an individual mouse. Error bars represent SEM of biological replicates. **d** MFP tumors and corresponding lung metastases from mice engrafted with PIM001-P tumors were analyzed by IHC using a CEACAM5 antibody. Scale bars indicate 100 μm. **e**, **f** IHC of serial tumor tissue sections from mice engrafted with BC3_A2 (**e**) or PIM001-P (**f**) stained with antibodies specific for CEACAM5 (top panel) or vimentin. Scale bars indicate 100 μm. **g** Expression of CEACAM5 (top panel) or vimentin (bottom panel) was determined in a panel of breast cancer cell lines. Error bars represent SEM of technical triplicates. **h** IHC was performed to monitor phosphorylated p38 in MFP tumors and lung metastases of BC3_A2 mice. Scale bars indicate 100 μm

We found that CEACAM5 expression was dynamically regulated as metastatic subpopulations were serially passaged between lungs and MFPs in vivo (Fig. 3a), confirming findings from RNAseq (Supplementary Table 1 and Supplementary Fig. 6b). Similarly, CEACAM5 expression was higher in liver (Supplementary Fig. 7a) and brain (Supplementary Fig. 7b) metastases relative to corresponding MFP tumors. CEACAM5 protein levels were also higher in lung metastases relative to MFP tumors (Fig. 3b). The increase in CEACAM5 expression in metastatic lesions was not dependent on p53 silencing in this PDX line, as lung metastases from the isogenic PDX line expressing wild type p53^26,30^ also displayed higher expression levels of CEACAM5 (Supplementary Fig. 7c).

A second set of PDX models of TNBC (PIM001) were evaluated to determine if the increase in CEACAM5 in metastatic lesions was a general property of metastasis. PIM001-P was established from the treatment-naïve primary tumor of a patient with metastatic TNBC, whereas PIM001-M was established from the dermal metastasis from the same patient. PIM001-P tumor cells were engineered to express both CBR-Luc and mCherry and then engrafted in the MFPs of recipient mice. At time of necropsy, MFP tumors and lung metastases were isolated. CEACAM5 expression was increased at both the mRNA-levels (Fig. 3c) and protein-levels (Fig. 3d) in PIM001-P lung metastases compared with corresponding MFP tumors. Interestingly, CEACAM5 protein levels were higher in PDX MFP tumors established from the patient’s dermal metastasis (PIM001-M) compared with the PDX MFP tumors established from her primary breast tumor (PIM001-P) (Supplementary Fig. 7d). Collectively, these results demonstrate that elevation of CEACAM5 is a common property of metastases in PDX models of TNBC.

### CEACAM5 expression is inversely correlated with vimentin expression and phosphorylation of p38

Because expression of CEACAM5 in PDX models was inversely correlated with expression of vimentin (Figs. 1d, 3a; Supplementary Figs. 2a and 7a, b), immunohistochemistry (IHC) was employed to monitor lung metastases and MFP tumors for relative levels of vimentin and CEACAM5. Protein levels correlated with mRNA levels in both PDX models (Fig. 3e, f). Vimentin expression was also inversely correlated with CEACAM5 expression in a panel of breast cancer cell lines (Fig. 3g). The serine/threonine-specific protein kinase p38alpha (also known as MAPK14, hereafter referred to as p38) is phosphorylated during EMT.^31^ Interestingly, phosphorylated (active) p38 correlated with high expression levels of vimentin in MFP tumors, and p38 phosphorylation decreased in lung metastasis where vimentin levels were low (Fig. 3h). Collectively, these data demonstrate that CEACAM5 expression is inversely correlated with expression of EMT markers in lung metastases.

### Ectopic overproduction of CEACAM5 inhibits TGF-β signaling and expression of EMT markers

**Fig. 4:**
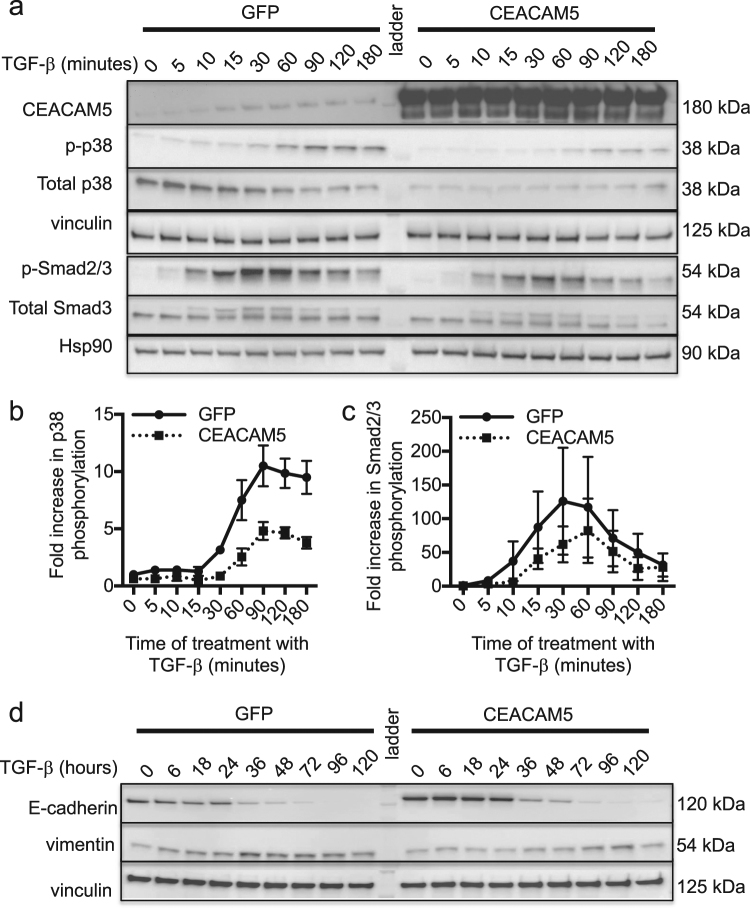
CEACAM5 overproduction downregulates expression of mesenchymal markers and impairs TGF-β signaling and impairs TGF-β signaling. **a** BC3_A2 cells engineered to overproduce CEACAM5 or GFP were cultured in the presence or absence of 1 ng/ml TGF-β for the indicated time periods. Cellular lysates were subjected to Western blotting for the indicated proteins. **b**, **c** Histogram shows the fold change (log scale) in phosphorylation status of p38 (**b**) or Smad2/3 (**c**) compared with that at time point 0 for cells expressing GFP. Error bars represent SEM of at least three independent experiments. **d** BC3_A2 cells expressing GFP or CEACAM5 were cultured in the presence or absence of 1 ng/ml TGF-β for the indicated time periods. Cellular lysates were subjected to Western blotting for the indicated proteins. All results are representative of at least three independent experiments. See also Supplementary Figs. 8 and 9

Given that CEACAM5 expression inversely correlated with mesenchymal status, we evaluated whether CEACAM5 had a direct role in regulating expression of mesenchymal markers. BC3_A2 cells engineered to overproduce human CEACAM5 or GFP as a negative control (Supplementary Fig. 8a, b) were examined for expression of vimentin and CDH2/N-cadherin (mesenchymal markers) and CDH1/E-cadherin (epithelial marker). Overexpression of CEACAM5 led to increased expression of CDH1 (Supplementary Fig. 8c) and decreased expression of CDH2 (Supplementary Fig. 8d) and vimentin (Supplementary Fig. 8e). In addition, overproduction of CEACAM5 reduced and delayed TGF-β mediated phosphorylation of Smads in BC3_A2 cells (Fig. 4a, c, Supplementary Fig. 9a) and p38 phosphorylation in both BC3_A2 cells (Fig. 4a, b, Supplementary Fig. 9a) and MDA-MB-231 cells (Supplementary Fig. 8f, Supplementary Fig. 9b). Finally, overproduction of CEACAM5 delayed TGF-β-induced EMT as assessed by E-cadherin and vimentin expression (Fig. 4d, Supplementary Fig. 9c). These results suggest that CEACAM5 negatively regulates EMT and identifies a function for CEACAM5 in breast cancer metastasis.

### Overproduction of CEACAM5 promotes metastatic outgrowth and reduces EMT in vivo

**Fig. 5:**
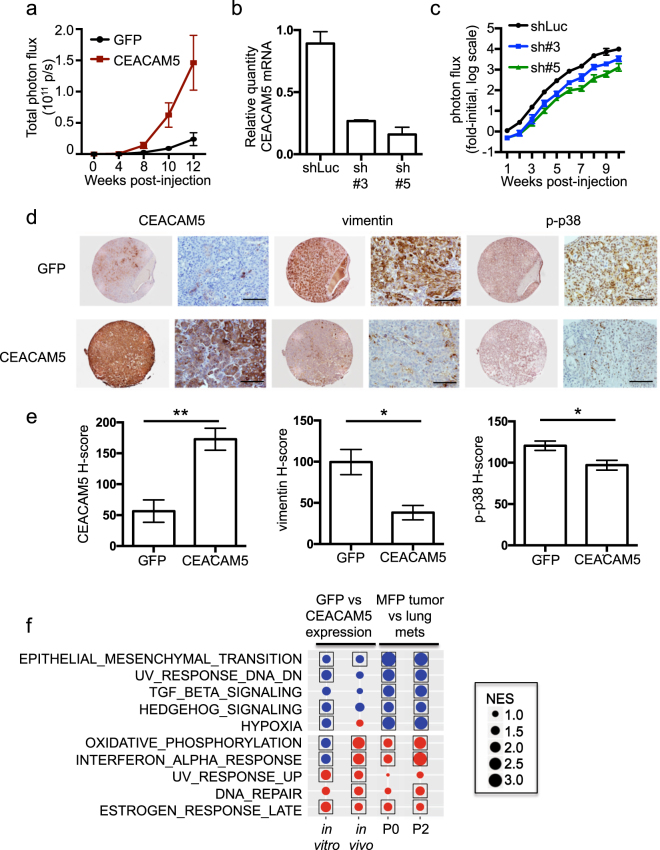
Expression of CEACAM5 promotes growth of lung metastases. **a** BC3_A2 cells overproducing CEACAM5 or GFP were injected into the tail veins of mice. BLI was used to quantify photon flux in the lungs of mice at the indicated time points. Paired t-test, *p* = 0.03. Error bars represent SEM of biological replicates (*n* = 5 mice per group). **b** BC3_A2 cells expressing control shRNA (shLuc) or two different shRNAs (#3 and #5) specific for CEACAM5 were subjected to qRT-PCR to monitor CEACAM5 expression. Error bars represent SEM of technical triplicates. See also Supplementary Fig. 10. **c** BC3_A2 cells expressing shLuc or two different CEACAM5 shRNAs were injected into the tail veins of mice. BLI was used to quantify photon flux in the lungs of mice at the indicated time points. Paired *t*-tests, *p* = 0.01 for sh#3 and *p* < 0.001 for sh#5. Data were normalized to the initial time point and are presented on a log scale. Error bars represent SEM of biological replicates (*n* = 5 mice per group). **d** BC3_A2 cells overproducing CEACAM5 were injected into the tail veins of mice, and lungs were isolated and subjected to IHC with the indicated antibodies. Scale bars indicate 100 μm. **e** Cells staining positive for CEACAM5, vimentin, or p-p38 in the tissue sections represented in panel d were quantitated using the Vectra 3.0 automated imaging system. Paired *t*-tests, *p* = 0.0008 for CEACAM5, *p* = 0.03 for vimentin, and *p* = 0.03 for p-p38. Error bars represent SEM of at least three independent biological replicates. **f** RNA isolated from BC3_A2 cells overproducing CEACAM5 or GFP and cultured either in vitro or isolated from the lung after tail vein injection (in vivo, **a**, **d**) was subjected to GSEA pathway analysis (left two columns). The top 5 down-regulated (blue) and top 5 up-regulated (red) processes are shown. Boxes indicate FDR <0.1 for statistical significance. NES (normalized enrichment score). Significance of these pathways in P0 and P2 MFP tumors vs. corresponding lung metastases is shown for comparison (right two columns). Data are represented of biological triplicates. See also Supplementary Fig. 11

EMT is associated with reduced cellular proliferation.^19^ Thus, the ability of CEACAM5 to inhibit EMT suggested that it may function to promote tumor outgrowth at metastatic sites. To test this hypothesis, BC3_A2 cells engineered to overproduce CEACAM5 or GFP (control) were injected into the tail veins of recipient mice to monitor the effects of CEACAM5 overproduction on tumor growth in the lung. Overexpression of CEACAM5 led to increased tumor growth in lungs (Fig. 5a and Supplementary Fig. 10a), decreased expression of vimentin at the protein level (Supplementary Fig. 10b), and decreased phosphorylation of p38 (Fig. 5d, e). Reducing CEACAM5 levels in BC3_A2 with two different shRNAs (Fig. 5b) had the opposite effect in that transduced cells grew more slowly in the lungs than did control cells following tail vein injection (Fig. 5c and Supplementary Fig. 10c). In addition, CEACAM5 knockdown resulted in increased lesion number but decreased lesion size (Supplementary Fig. 10d, e). These results suggest that CEACAM5 silencing promotes EMT and tumor cell extravasation while at the same time inhibiting metastatic outgrowth.

To monitor how overproduction of CEACAM5 impacted gene expression, BC3_A2 cells engineered to overproduce CEACAM5 or GFP were cultured in vitro or isolated from the lung after tail vein injection (in vivo). RNA was isolated and subjected to RNAseq. Differential gene expression analysis was performed, and GSEA identified EMT as the only cancer hallmark pathway that was significantly downregulated both in vitro and in vivo by CEACAM5 overproduction (Fig. 5f, Supplementary Fig. 11). This result is similar to that observed in P0 and P2 lung metastases (Figs. 1c, 5f). Collectively, these data indicated that upregulation of CEACAM5 expression in metastatic lesions induces MET and enhances the outgrowth of metastatic tumor cells in secondary sites.

### CEACAM5 expression is upregulated in metastatic lesions from breast cancer patients

**Fig. 6:**
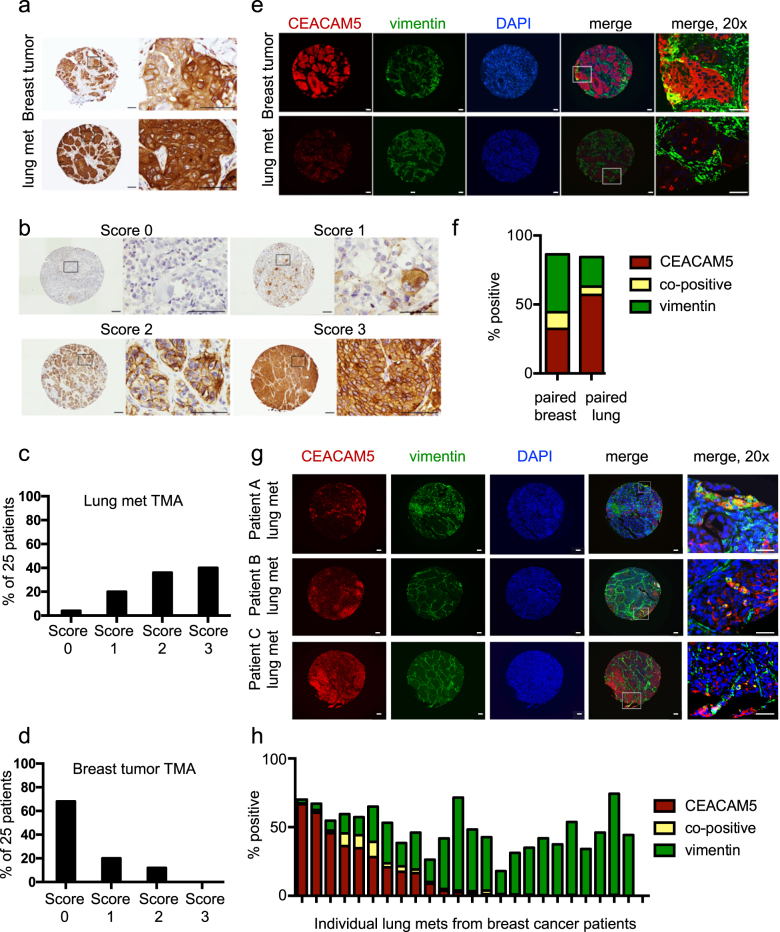
CEACAM5 protein levels are elevated in metastatic lesions of breast cancer patients and are inversely correlated with expression of vimentin. **a** The primary tumor and corresponding lung metastasis from a patient with breast cancer were subjected to IHC to monitor CEACAM5 levels. Scale bars indicate 100 μm. **b** A tumor tissue microarray (TMA) consisting of lung metastases from 25 breast cancer patients was subjected to IHC with a CEACAM5-specific antibody, and staining intensity was scored. Representative images are shown. Scale bars indicate 100 μm. **c** The scoring system in **b** was applied to the TMA consisting of lung metastases from 25 breast cancer patients to assess relative CEACAM5 levels. **d** The scoring system in **b** was applied to a TMA from the Human Protein Atlas consisting of 25 primary breast tumors to assess relative CEACAM5 levels. **e** The primary tumor and corresponding lung metastasis from a patient with breast cancer (from **a**) were co-stained for CEACAM5 and vimentin and analyzed by immunofluorescence microscopy. Scale bars indicate 100 μm. **f** Cells staining positive for CEACAM5, vimentin, or both in the tissue sections represented in **e** were quantitated using the Vectra 3.0 automated imaging system. **g** The TMA consisting of lung metastases from 25 breast cancer patients was co-stained for CEACAM5 and vimentin and analyzed by immunofluorescence microscopy. Representative images are shown. Scale bars indicate 100 μm. **h** Cells staining positive for CEACAM5, vimentin, or both in the tissue sections shown in **g** were quantitated using the Vectra 3.0 automated imaging system. See also Supplementary Fig. 12

Next, CEACAM5 expression was assessed in metastatic lesions and breast tumor tissue obtained from patients with breast cancer. IHC staining on a matched tissue set consisting of a primary tumor and a lung metastasis from a patient with breast cancer (Supplementary Table 3) revealed higher CEACAM5 levels in the lung metastasis relative to the primary breast tumor (Fig. 6a and Supplementary Fig. 12a). To expand these analyses, a tissue microarray (TMA) consisting of lung metastases from 25 breast cancer patients (Supplementary Figs. 12b, c and Supplementary Table 3) was stained for CEACAM5 (Supplementary Fig. 12b), and staining intensity was assigned a score (Fig. 6b). This scoring method was also applied to a TMA from the Human Protein Atlas consisting of human breast tumors stained for CEACAM5.^32^ The majority of lung metastases expressed high levels of CEACAM5 (Fig. 6c) compared with breast tumors (Fig. 6d), demonstrating that CEACAM5 was higher in metastatic lesions compared with primary breast tumors.

We next assessed the matched tissue set for co-expression of CEACAM5 and vimentin. Co-staining for CEACAM5 and vimentin revealed higher levels of CEACAM5 in the lung metastasis relative to matched breast tumor, and the reverse staining pattern was observed for vimentin (Fig. 6e, f). In addition, a minor population of tumor cells exhibited co-staining for both CEACAM5 and vimentin (Fig. 6f). Those cells staining positive for both proteins may represent cells in a hybrid EMT/MET state.^33^ The TMA consisting of lung metastases from 25 breast cancer patients also demonstrated an inverse correlation between CEACAM5 and vimentin staining (Fig. 6g, h; Supplementary Figs. 12a–e). These results confirmed conclusions made with our PDX models of TNBC and demonstrated that CEACAM5 expression is inversely correlated with vimentin expression in primary breast tumors and metastatic lesions. Taken together, these results suggest that CEACAM5 promotes MET to facilitate tumor outgrowth at metastatic sites (Supplementary Figure 12a, b).

## Discussion

In this study, we utilized PDX models of TNBC and high throughput gain-of-function (GOF) screens in vivo to identify drivers of breast cancer metastasis (Supplementary Fig. 13a-b). Serial passaging of metastatic lesions from lung back into the MFPs of recipient mice enriched for metastatic potential rather than for specific homing of tumor cells to any particular organ. In addition, serial passaging of metastases enriched for 845 genes whose expression was significantly upregulated in lung lesions relative to MFP tumors. From this set of upregulated genes, a custom barcoded library encoding 230 ORFs was generated and used in a high-throughput pooled-format gain-of-function screen to evaluate the ability of each gene to drive metastasis in vivo. CEACAM5 was identified as a metastatic driver in this screen. CEACAM5 expression was enhanced in metastases relative to corresponding mammary fat pad (MFP) tumors, and its expression was inversely correlated with expression of mesenchymal markers in both PDX models and patient-derived tissue sections. Furthermore, CEACAM5 overproduction reduced expression of mesenchymal markers in breast cancer cells and promoted the outgrowth of metastatic lesions in the lungs of mice. These results identify a role for CEACAM5 in regulating the late stages of metastasis.

We observed that EMT markers were dynamically regulated as tumors were passaged between the lung and MFP in vivo. EMT signatures were high in MFP tumors and low in lung metastases. Metastatic tumor cells are commonly thought to rely on EMT to escape primary tumors in the initial stages of metastasis by downregulating intercellular adhesion and proliferation and acquiring invasive and stem cell-like properties.^20^ However, the significance of EMT to metastasis has been a long-standing matter of debate due, in part, to the fact that EMT is often difficult to observe in real time in vivo in immune-competent mouse models or in patients because mesenchymal tumor cells that have undergone EMT resemble fibroblasts in the surrounding host stroma. This confounding variable was circumvented in our studies as we employed PDX models, which enable human tumor cells to be distinguished from mouse stromal cells. Moreover, EMT is often a transient process, as the up-regulation of EMT programs in the primary site is associated with growth arrest and allows tumor cells to escape the primary tumor, but reversion to a more epithelial state (MET) is necessary for the upregulation of proliferation to establish macrometastases.^19,21,22^ Studies have shown that the pre-metastatic niche can also influence whether tumor cells will efficiently seed and proliferate there,^34^ but what initiates the switch to MET at the metastatic site has not been determined. Our data demonstrate that CEACAM5 has a role in this process and suggest that perturbation of MET programs may be a therapeutic strategy to restrain tumor outgrowth at the metastatic site.

Although MET programs may retard the growth of metastatic lesions, inhibition of MET in metastatic tumor cells may in turn lead to the upregulation of EMT-associated tumor cell migration, consequently resulting in secondary metastases. Thus, one limitation of this study is that clinical application of these findings may be a palliative, rather than curative, therapeutic strategy. Additionally, the mechanisms by which MET promotes lung metastasis have not been fully elucidated. Upregulation of MET may influence multiple steps in the metastatic cascade including increased cell survival in circulation, increased adhesion to blood vessels in secondary sites, or increased proliferation during metastatic colonization. Finally, it is important to note that these studies were conducted in immune-compromised mice, and thus, the contribution of the immune system to metastasis cannot be fully explored in this system. However, elevated expression of CEACAM5 was confirmed in metastatic lesions obtained directly from cancer patients, thus confirming the clinical relevance of our findings.

CEACAM5 is not expressed on most normal adult tissues including the breast; its expression is restricted to the apical surface of the gastrointestinal tract.^35^ High CEACAM5 expression in breast tumors correlates with reduced patient survival.^36–38^ Additionally, CEACAM5 is secreted into the blood of breast cancer patients, and elevated serum levels indicate progression to metastasis.^39^ CEACAM5 is also a well-recognized tumor and serum biomarker for the progression of colorectal and pancreatic cancers.^40,41^ We demonstrated that CEACAM5 expression is elevated in metastatic breast cancer cells compared with the corresponding primary breast tumors in PDX models of TNBC and in tissue sections obtained directly from breast cancer patients. In preclinical models, anti-CEACAM5 neutralizing antibodies have to been shown to affect cell adhesion, migration, and invasion in vitro, and pre-incubation of tumor cells with these antibodies resulted in decreased metastasis of tumor cells following intrasplenic injection.^42^ Collectively, these results suggest that CEACAM5 may serve as a tumor and serum biomarker for metastatic progression and may also be a viable therapeutic target.

Attempts to target CEACAM5 with anticancer therapeutic approaches have included the development and clinical implementation of vaccines, immunotherapies, and antibodies.^43^ However, the ability of these agents to target tumors and suppress their growth is limited, and relatively few drugs that directly target CEACAM5 have been developed. Accordingly, the most effective treatments are based on stimulation of immune responses against CEACAM5.^43^ Our results suggest that the development of novel therapeutic agents directly targeting CEACAM5 could be effective in inhibiting tumor outgrowth in the metastatic setting. However, one concern is that this could also promote migratory properties of tumor cells through expression of EMT markers thereby favoring mesenchymal (migratory)-like properties and secondary metastatic seeding events. Our finding that CEACAM5 can inhibit p38 activity and promote the growth of metastatic lesions agrees with previous studies showing that pharmacologic inhibition of p38 promotes the outgrowth of micrometastatic tumor cells, likely through induction of MET.^44^ Thus, metastatic stage should be carefully considered before clinical application of pharmacologic agents targeting these pathways.

Despite remarkable recent advances in pharmacologic development, outcomes for patients with metastatic breast cancer remain poor. Therefore, there is a clear need to elucidate the molecular mechanisms governing the progression of established metastatic tumors. Identifying molecules that promote macrometastasis, such as CEACAM5, is expected to lead to the development of new therapeutic targets and the application of existing therapies for patients with advanced metastatic disease.

## Methods

### Study approval

Similar to our previous studies,^28^ this study was carried out in accordance with the sanctions in the Guide for the Care and Use of Laboratory Animals from the National Institutes of Health (NIH) Institutional Animal Care and use Committee (IACUC). The IACUC protocol was approved by the Committee on the Ethics of Animal Experiments of Washington University and the IACUC at MD Anderson Cancer Center. Mice were humanely euthanized as dictated by the Association for Assessment and Accreditation of Laboratory Animal Care International and IACUC euthanasia endpoints when they became moribund or when they reached defined study end points. Animals were euthanized as dictated by the Association for Assessment and Accreditation of Laboratory Animal Care International and IACUC euthanasia end points. Informed consent was obtained from all living patients from whom data was included in this study.

### Establishment of PDX models

PDX models were established according to published methods.^28,45^ Briefly, GFP-labeled human stromal fibroblasts were irradiated with 400 Rads and mixed with an equal number of non-irradiated fibroblasts (2.5 × 10^5^ irradiated cells and 2.5 × 10^5^ non-irradiated cells). Fibroblasts were then mixed with 1 × 10^6^ BC3_A2 tumor cells and 1/3 volume Matrigel. This mixture was injected into the fourth mammary fat pads of 4 to 5-week-old female nonobese diabetic/severe combined immunodeficiency (NOD/SCID) mice (NOD.CB17-Prkdc^scid^/NcrCrl, Charles River, NCI Colony). PIM001-P and PIM001-M tumor cells were not mixed with fibroblasts prior to implantation. Tumors were resected in a survival surgery when they reached approximately 1 cm in diameter. Animals were euthanized when they became moribund or a maximum of 30 weeks post-tumor-implantation.

### Serial passaging of tumors in vivo

Metastases were isolated at animal necropsy using bioluminescence-guided macro-dissection. Tissues were digested to single cells and organoids in collagenase and hyaluronidase for 4 h at 37 ^o^C on a rotator. Tumor cells were washed in DMEM/F12 with 5% bovine calf serum, and red blood cells were lysed for 5 min at room temperature (RBC lysis buffer; Sigma-Aldrich R7757). Cells were passed through a 70 μm filter and counted using a fluorescence-coupled microscope (only the mCherry-positive cells were counted). A maximum of 1 × 10^6^ mCherry-positive tumor cells were then implanted into the fourth mammary fat pads of recipient mice, along with irradiated and non-irradiated human stromal fibroblasts and 1/3 volume Matrigel.

### Analysis of qPCR data for in vivo screen

Barcode quantification was carried out using the DDCt method.^46^ First, barcode quantification cycle (Cq) values were normalized to total DNA with the Cq value for DNA reference probe. Next, tumor sample barcode values were converted into a fold change relative to the respective pooled reference sample. ΔΔCt of the lung vs. the mammary was also calculated for each matched pair, and samples with three or more matched pairs were averaged to rank hits. Samples with fewer than three data points were not considered for analysis. All genes with an average depletion of at least two-fold in the mammary relative to the reference pool were considered as putative metastatic drivers (hits).

### RNAseq and data analysis

Tumors were harvested in a survival surgery when they reached 1 cm and were immediately snap frozen in liquid nitrogen. Metastatic lesions were harvested by bioluminescence-guided macro-dissection and immediately snap frozen in liquid nitrogen. Mice with the largest metastatic lesions were selected for RNAseq. Similar to our previous studies,^28^ tumors were snap frozen to preserve RNA integrity and avoid experimental manipulations that could lead to alterations in gene expression. Tumors were stored at −80 °C, thawed on ice in Trizol, and homogenized with a pedestal homogenizer. Total RNA was extracted with chloroform followed by purification using the RNEASY kit (Qiagen, Venlo, The Netherlands; 74104). Total RNA was treated with DNase (TURBO DNase, Life Technologies, Carlsbad, CA, USA; AM2238) followed by column purification (RNA Clean & Concentrator-5, Zymo Research, Irvine, CA, USA; R1015). mRNA was isolated by poly-A selection. RNA Integrity Number (RIN) was assessed on a Bioanalyzer (Agilent Technologies, Santa Clara, CA, USA). RNAseq libraries were prepared and sequencing was performed by the Genome Technology Access Center (Washington University, St. Louis, MO, USA). RNAseq reads were first mapped to the mouse reference genome (UCSC mm10) using Tophat2 (v2.0.14) and Bowtie2 (v2.2.2), allowing a maximum of two mismatches per 75 bp read end. The unmapped reads were then aligned to the human genome (UCSC hg19) using the same tool and parameters. Gene-level read counts were quantified using HTseq (http://www.huber.embl.de/users/anders/HTSeq/doc/overview.html). Normalization and differential expression analyses for gene/isoform expression were performed using DESeq2.^47^ The log2 fold-change 1.5 and adjusted *p*-value cutoff <0.05 were used to identify differentially expressed genes. log10 *p*-values from the differential analysis were used to build the rank file for pre-ranked GSEA against the Cancer Hallmark pathways.^48,49^ Numbers of mice included in each analysis were as follows: P0 MFP tumors, *n* = 3; P0 lung metastases *n* = 4; P2 MFP tumors, *n* = 3; P2 lung metastases, *n* = 3; BC3_A2 cells expressing GFP and injected into tail veins, *n* = 3; BC3_A2 cells expressing CEACAM5 and injected into tail veins, *n* = 3.

### Statistics

Photon fluxes were normalized to the time of study end point as described previously.^28^ Wilcoxon rank sum tests were used to evaluate the mean differences in photon flux in each organ due to relatively small sample size in most groups. Hierarchical clustering of RNAseq data was performed using Euclidean distance and complete linkage. PCA plots and heat maps, and statistical analyses were performed using R (http://www.r-project.org/).

### Kaplan–Meier survival analysis

Similar to our previous studies,^28^ the univariate Cox proportional hazard model was used to assess the correlation of gene expression with patient overall survival, and the log-rank *p*-value was reported. The dataset from The Cancer Genome Atlas (TCGA)^50^ was used for determining overall survival of breast cancer patients. Log-rank tests were used to estimate the *p*-values between high and low expression groups using the top and bottom 10% as the cutoff for grouping.

### Data availability

RNAseq data are publically available at Sequence Read Archive (SRA), accession numbers SRP133840.

## Electronic supplementary material

Supplementary Figures

Supplementary Table 1

Supplementary Table 2

Supplementary Table 3

Supplementary Table 4

Supplementary methods
