# Supplementary material for: A functional genomic screen in vivo identifies CEACAM5 as a clinically relevant driver of breast cancer metastasis
Source: NPJ Breast Cancer. 2018 Apr 30;4:9. doi: 10.1038/s41523-018-0062-x (PMC5928229; doi:10.1038/s41523-018-0062-x)
Supplement: Supplementary file 1 — Supplementary Figures [file 41523_2018_62_MOESM1_ESM.pdf]

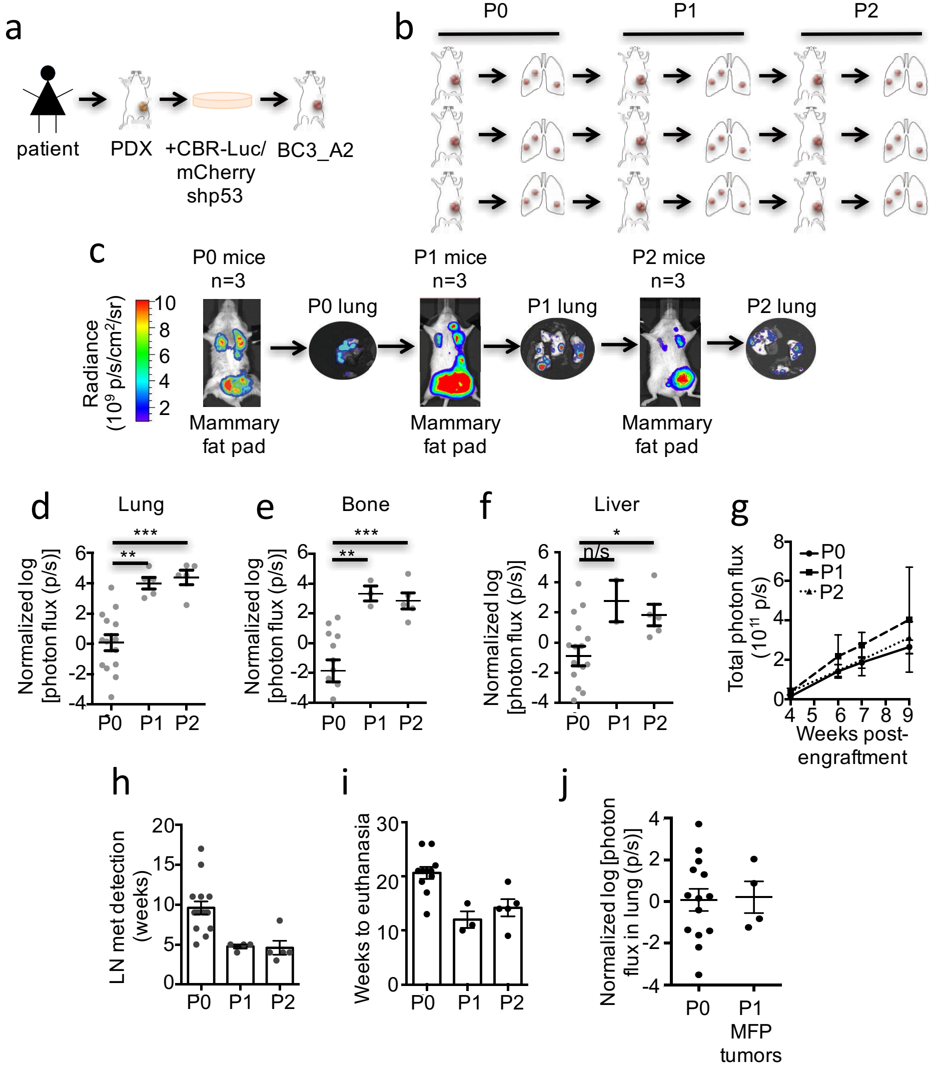

Supplementary Figure 1. Serial passaging of metastases between lung and MFP *in vivo* enriches for metastatic potential. (a) Schematic representation depicting the generation of PDX line BC3_A2. (b) Diagram of the protocol used for serially passaging lung metastases *in vivo*. (c) Representative bioluminescent images of mice and lung metastases during serial passaging of tumors. Also see Figure 1. (d-f) Quantification of photon flux from bioluminescent images of lungs (d), bones (e), and livers (f) following passaging of metastatic subpopulations *in vivo* and normalization to time post-tumor engraftment to euthanasia. Wilcoxan rank-sum tests: lung metastases, p= 0.0007 for P1 and p= 0.0003 for P2; bone metastases, p= 0.004 for P1 and 0.0005 for P2; liver metastases, p= 0.08 for P1 and 0.03 for P2. Each data point represents one mouse. (g) Total photon flux from MFP tumors at each passage was quantified from BLI *in vivo,* and values were plotted as a function of time. Wilcoxan rank-sum test, p= 0.6 (n/s). All error bars represent standard error of the mean (SEM) of biological replicates. At least 3 mice were included in each group. (h) BLI was performed biweekly to detect lymph node metastases, and time to detection is shown. Each data point represents one mouse. Wilcoxan rank-sum test: p = 0.004 for P1 and 0.003 for P2. (i) Time to euthanasia (in weeks) following tumor implantation is shown. Each data point represents one mouse. This data was used for normalization of photon flux from metastases. Wilcoxan rank-sum test, p= 0.02 for P1 and p=0.01 for P2. (j) Quantification of photon flux from bioluminescent images of lungs after passaging of MFP tumor cells *in vivo*. Wilcoxan rank-sum test, p= 0.98 (n/s). Each data point represents one mouse. All error bars represent SEM of biological replicates.

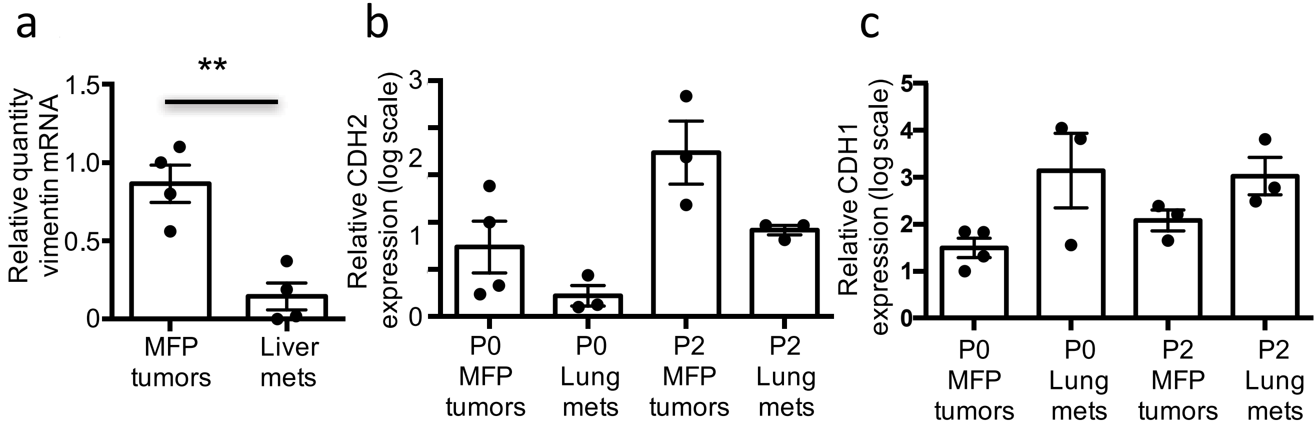

Supplementary Figure 2. Mesenchymal markers are down-regulated in metastatic lesions relative to MFP tumors. (a) qRT-PCR analysis of vimentin expression of BC3_A2 MFP tumors and liver metastases. Paired t-test, p= 0.002. Each data point represents one mouse. (b) qRT-PCR analysis of N-cadherin (CDH2) expression in BC3_A2 MFP tumors and lung metastases. Paired t-tests, p= 0.19 for P1 lung, p= 0.07 for P2 lung. Each data point represents one mouse. (c) qRT-PCR analysis of E-cadherin (CDH1) expression in BC3_A2 MFP tumors and lung metastases. Paired t-tests, p= 0.12 for P1 lung, p= 0.11 for P2 lung. Each data point represents one mouse. All error bars represent SEM of biological replicates.

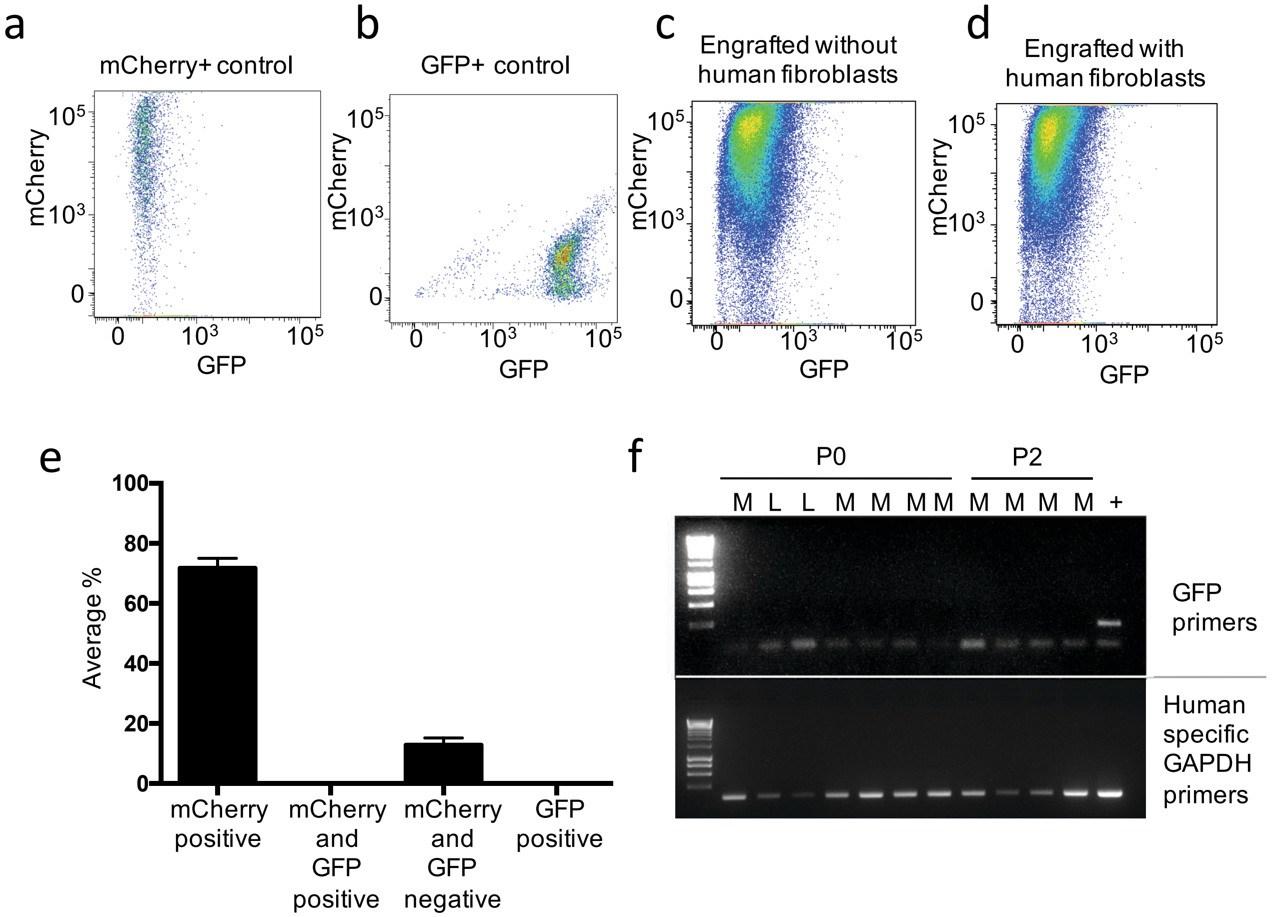

Supplementary Figure 3. Engrafted stromal fibroblasts are cleared from MFP tumors. (a-d) mCherry and GFP FACS profiles of BC3_A2 tumor cells cultured in vitro (a), GFP-positive stromal fibroblast cells cultured in vitro (b), BC3_A2 tumors that were engrafted into the MFP without fibroblasts and harvested 8 to 10 weeks post-implantation (c), and BC3_A2 tumors that were co-engrafted into the MFP with GFP-expressing fibroblasts and harvested 8 to 10 weeks after engraftment (d). (e) Quantification of FACS analysis in panels A-D. Results represent 3 independent experiments. Error bars represent SEM of biological triplicates. (f) PCR using genomic DNA from BC3_A2 MFP tumors (M) and lung metastases (L) as a template to assess the presence of GFP-positive human stromal fibroblasts. gDNA from GFP-positive EG fibroblasts was used as a positive control (+).

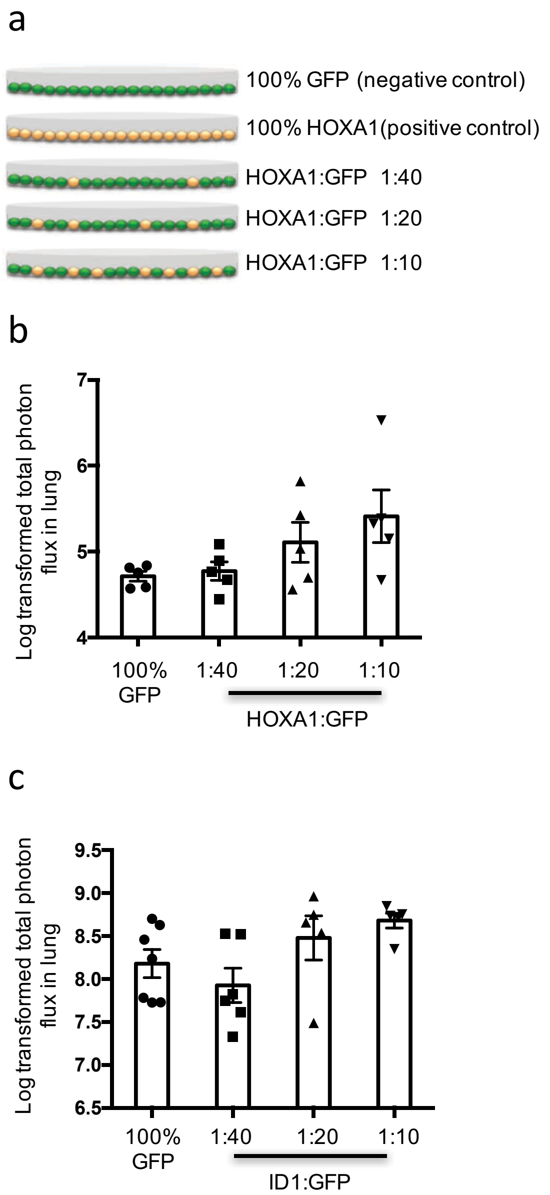

Supplementary Figure 4. Optimization of conditions for high-throughput GOF screen *in vivo.* (a) Schematic diagram depicting complexity test design. (b) A complexity test was performed in BC3_A2 cells following injection of cells into MFPs. Photon fluxes from the lungs were quantified and plotted. Wilcoxan rank-sum tests, p= 0.05 for 1:10, p= 0.69 for 1:20, and p= 0.69 for 1:40. Each data point represents one mouse. (c) A complexity test was performed in BC3_A2 cells following injection of cells into the tail vein. Photon fluxes from the lungs were quantified and plotted. Wilcoxan rank-sum tests, p= 0.02 for 1:10, p= 0.27 for 1:20, and p= 0.26 for 1:40. Each data point represents one mouse. All error bars represent SEM of biological replicates.

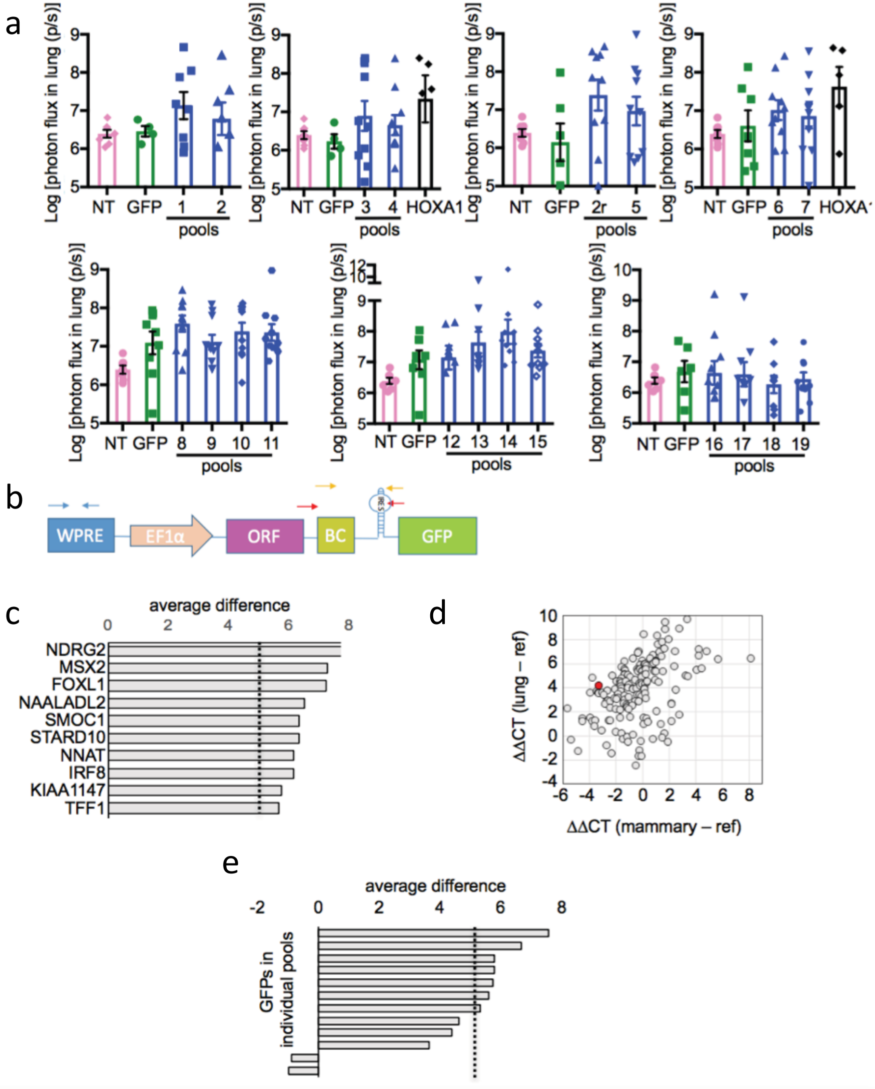

Supplementary Figure 5. High throughput GOF screen identifies metastatic sites with increased photon flux. (a) Quantification of photon fluxes from lungs of mice engrafted with pools of 12 cell lines each overexpressing one putative metastasis driver. Each data point represents one mouse. NT, non-transduced. All error bars represent SEM of biological replicates. (b) Schematic showing primer pairs used for qPCR assay for screen. Blue arrows represent binding sites for lentiviral integration control primers; red arrows represent binding sites for barcode amplification control primers; yellow arrows represent binding sites for primers that bind directly to unique barcode sequences. (c) Genes enriched in lung metastases and MFP tumors (upper right quadrant of Fig. 3B) were ranked in descending order of enrichment in lung. Dotted line indicates enrichment score cutoff. (d) Scatter plot showing relative representation of each ORF in MFP tumors after normalizing MFPs (x axis) and lungs (y axis) to the reference. CEACAM5 is shown in red. (e) Representation of barcodes associated with GFP included in each pool are shown. The DDCT method was used for quantification of qPCR data. Dotted line indicates enrichment score cutoff. See also Fig. 2 and Supplementary Table 2.

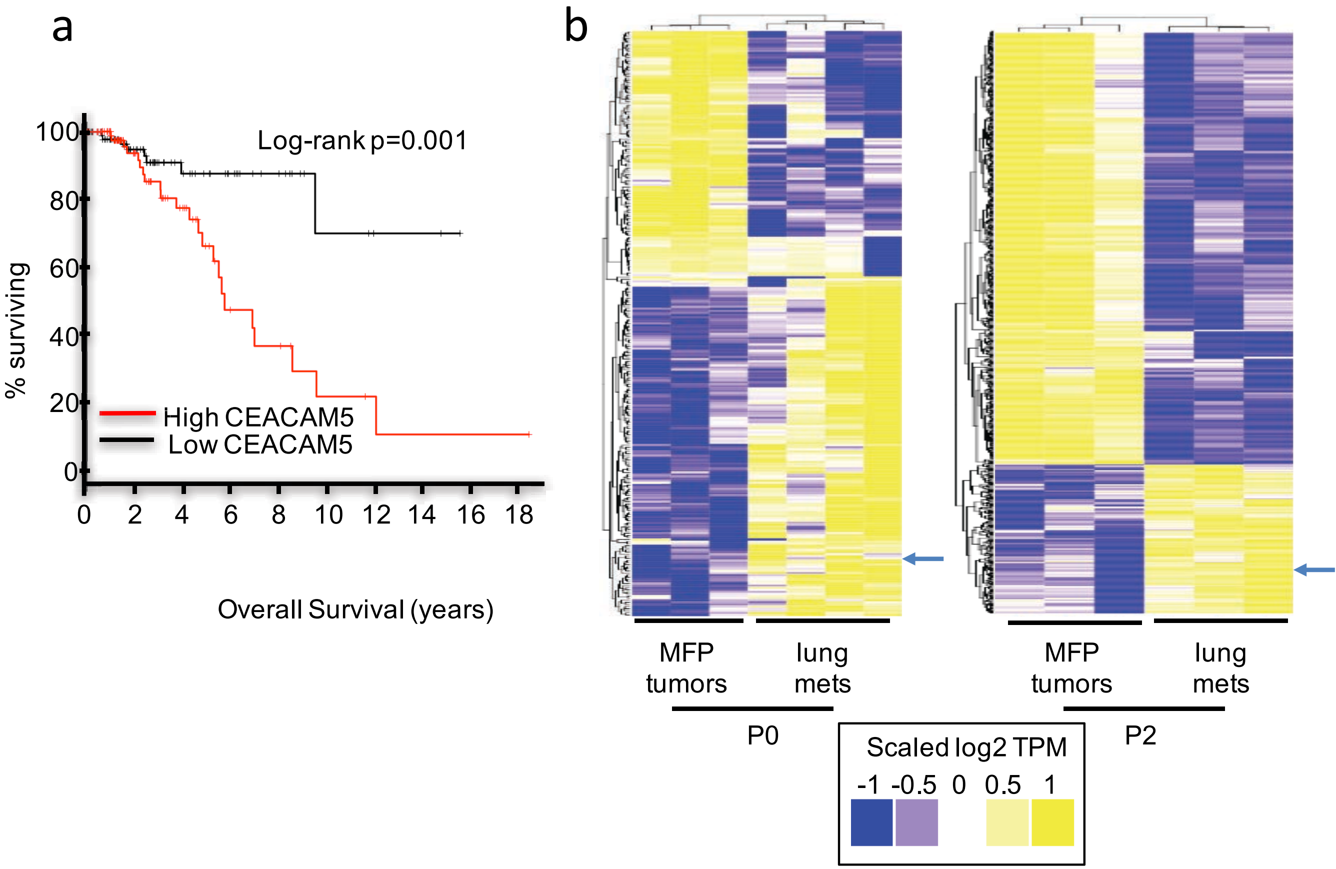

Supplementary Figure 6. High CEACAM5 expression correlates with decreased overall survival of breast cancer patients. (a) Kaplan-Meier curves were generated to assess correlations between CEACAM5 expression and overall patient survival. Top and bottom 10% of patients were used as cutoffs for grouping. (b) Heat maps of RNAseq gene expression signatures for BC3_A2 P0 and P2 lung metastases versus MFP tumors are shown. Arrows denote CEACAM5.

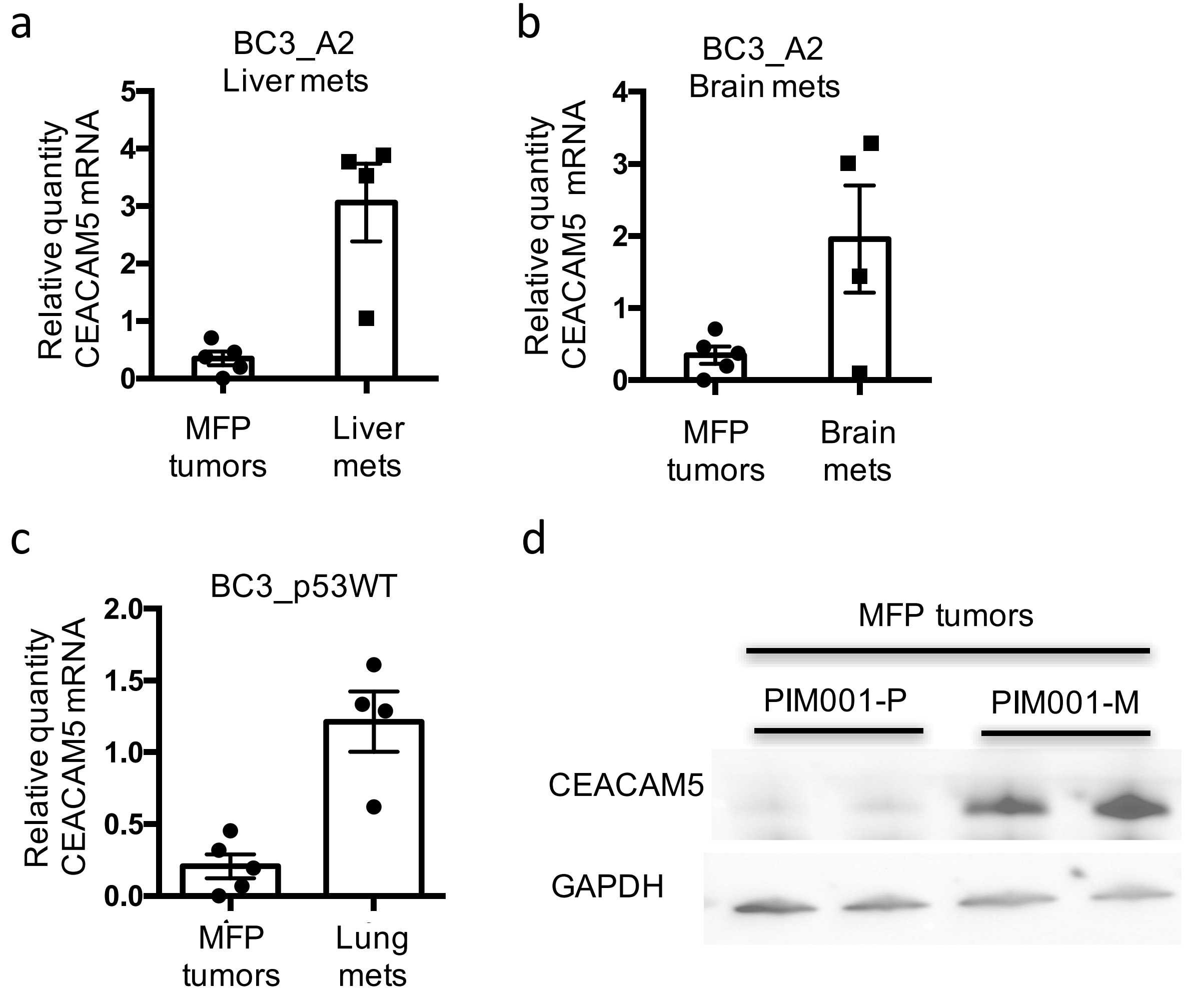

Supplementary Figure 7. CEACAM5 expression is up-regulated in metastatic lesions compared with corresponding MFP tumors. (a) qRT-PCR analysis of CEACAM5 expression in BC3_A2 MFP tumors and liver metastases. Paired t-test, p= 0.02. (b) qRT-PCR analysis of CEACAM5 expression in BC3_A2 MFP tumors and brain metastases. Paired t-test, p= 0.05. (c) qRT-PCR analysis of CEACAM5 expression in p53 wild type BC3 tumors and lung metastases. Paired t-test, p= 0.04. Each data point represents one mouse. All error bars represent SEM of biological replicates. (d) Western blotting on lysates from paired PDX (PIM001-P and PIM001-M) MFP tumors using the indicated antibodies. Biological duplicates from independent mice are shown.

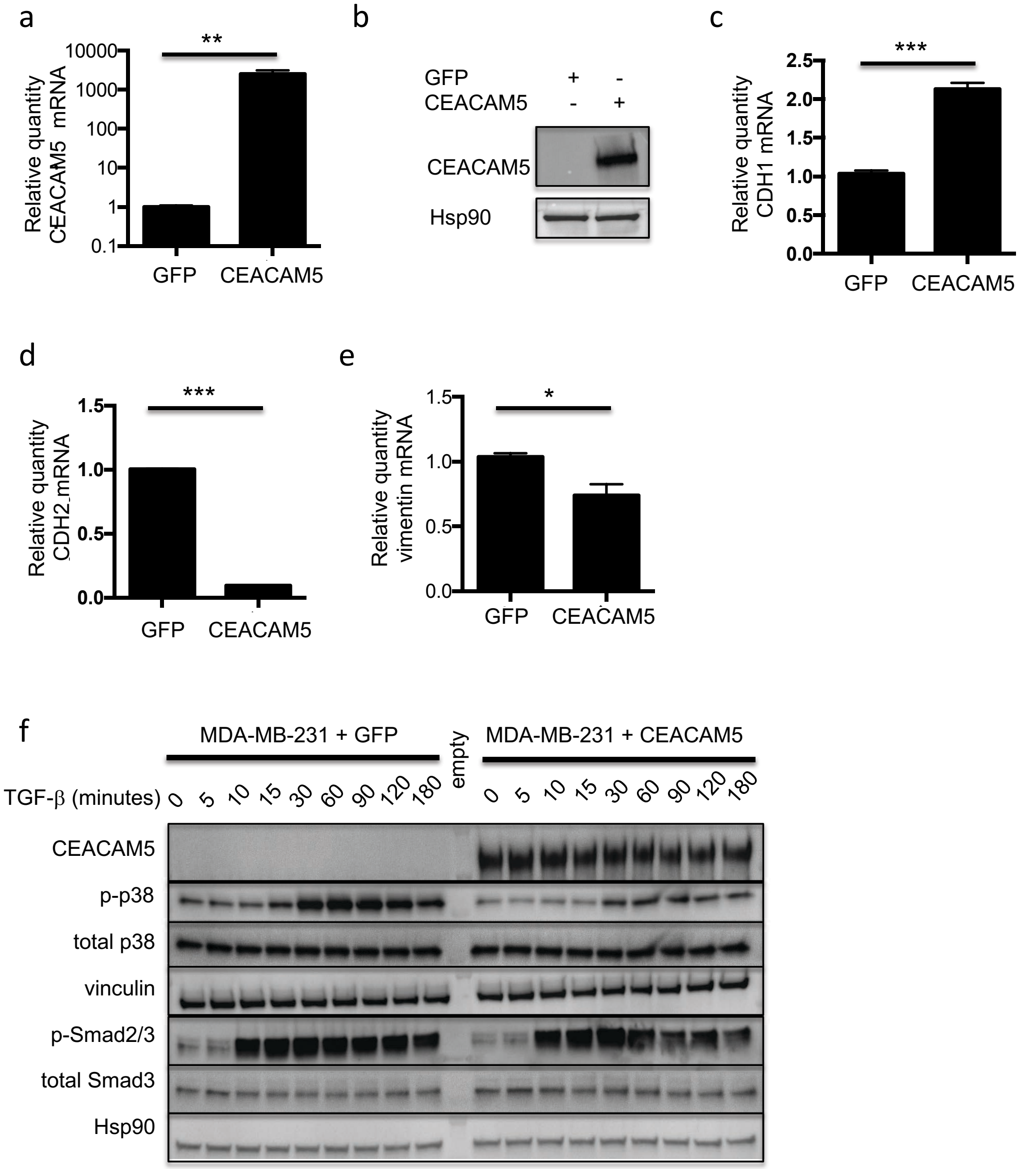

Supplementary Figure 8. Effects of CEACAM5 overproduction on expression of mesenchymal- and epithelial-markers and TGF-b signaling. (a) qRT-PCR analysis of CEACAM5 expression in BC3_A2 cells overproducing CEACAM5 or GFP. Error bars represent SEM of technical triplicates. Paired t-test, $p = 0.002$. (b) Western blot analysis of CEACAM5 protein levels in BC3_A2 cells overproducing CEACAM5 or GFP. (c-e) BC3_A2 cells overproducing CEACAM5 or GFP were analyzed by qRT-PCR for expression of E-cadherin (CDH1) (c), N-cadherin (CDH2) (d), or vimentin (e). Paired t-tests, $p < 0.001$(c), $p < 0.001$ (d), and $p = 0.03$ (e). All error bars represent SEM of technical triplicates. (f) MDA-MB-231 cells overproducing CEACAM5 or GFP were cultured or absence of 1 ng/ml TGF-β for the indicated time periods. Western blotting was performed for the indicated proteins. See also Fig. 4.

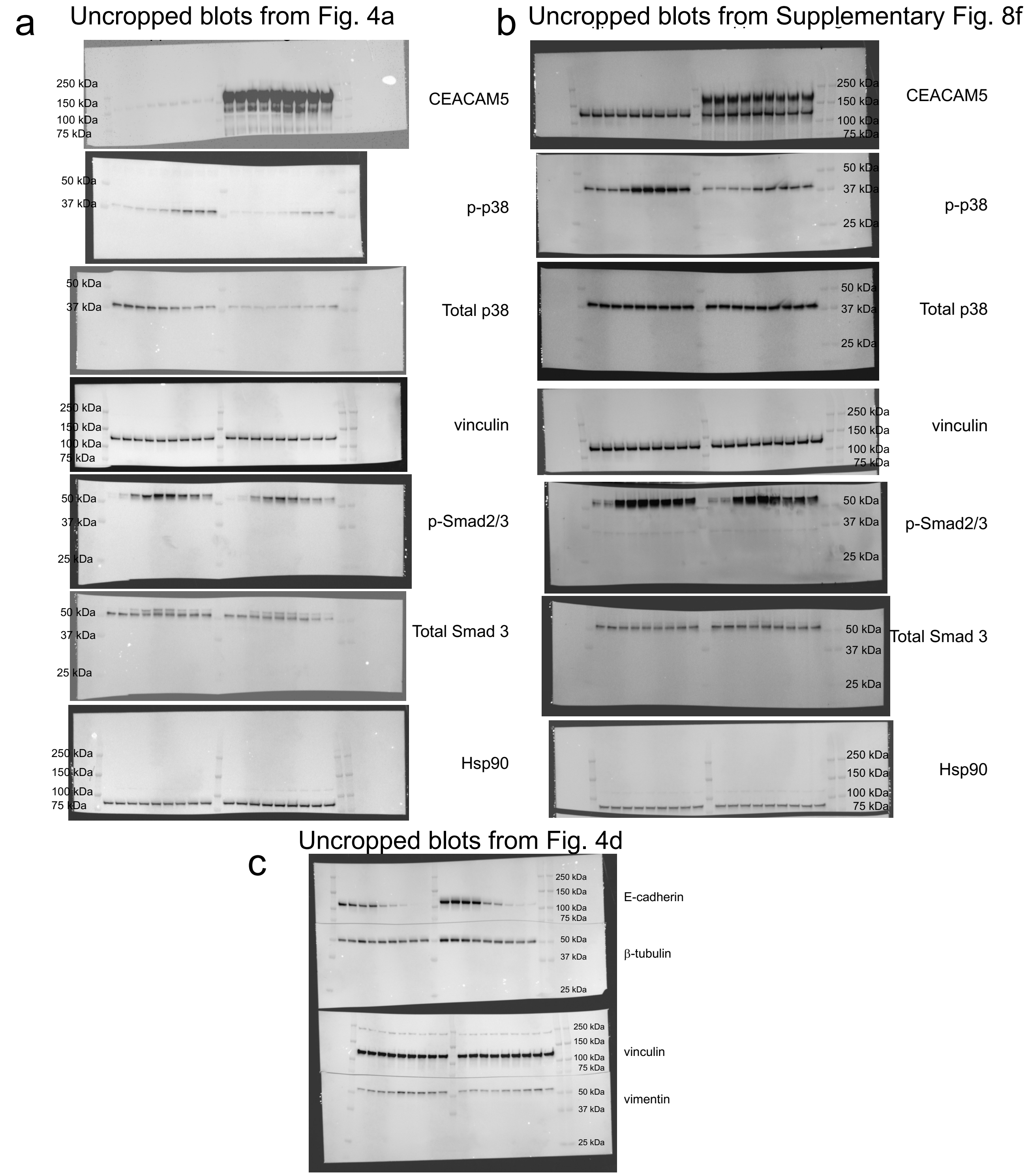

Supplementary Figure 9. Uncropped Western blots. Corresponding figures are indicated, and molecular weight markers are shown.

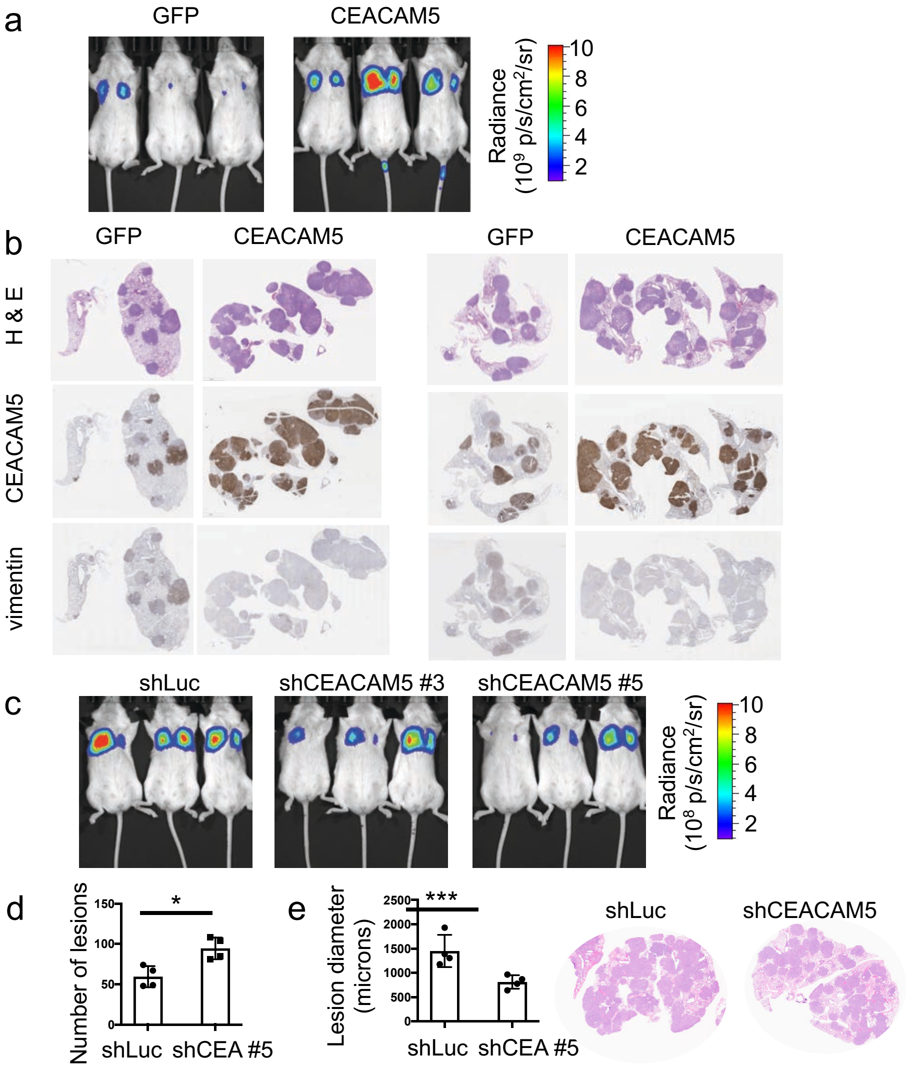

Supplementary Figure 10. Inverse correlation between CEACAM5 and vimentin protein levels *in vivo*. (a) Representative bioluminescence images for the mice represented in Figure 5A. Images were captured 10 weeks after tumor cell injection. (b) BC3_A2 cells engineered to overproduce CEACAM5 or GFP were injected into the tail veins of recipient mice. FFPE lung sections, were stained with H&E or with antibodies specific for CEACAM5 or vimentin. See also Fig. 5. (c) Representative bioluminescence images for the mice represented in Figure 5C. Images were captured 9 weeks after tumor cell injection. (d) Tissue sections of lungs from mice injected with BC3_A2 cells expressing shLuc or shCEACAM5 #5 were stained with H&E, and metastatic foci were counted. Paired t-test, p= 0.01. Error bars represent SEM of biological replicates (n=4 mice). (e) Tissue sections of lungs from mice injected with BC3_A2 cells expressing shLuc or shCEACAM5 #5 were stained with H&E, and the diameter of each lesion was measured. Paired t-test, p< 0.001. Error bars represent SEM of biological replicates (n=4 mice). Representative images of H&E sections are shown in the right panel.

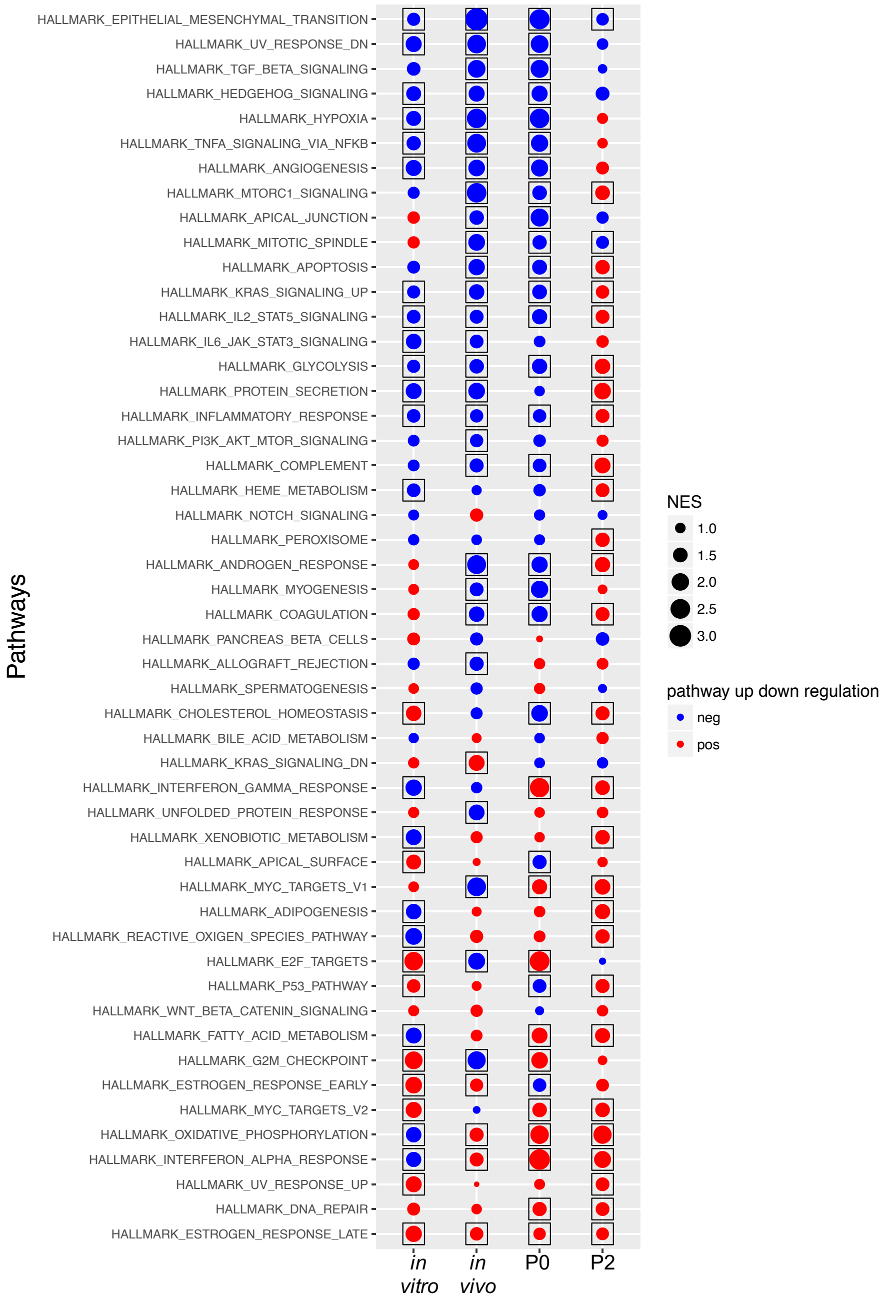

Supplementary Figure 11. Pathways altered by CEACAM5 overproductions. RNA isolated from BC3_A2 cells overproducing CEACAM5 or GFP and cultured either *in vitro* or isolated from the lung after tail vein injection (*in vivo*) was subjected to GSEA pathway analysis (left two columns). Down (blue)- and up (red)-regulated processes are shown. Boxes indicate FDR<0.1 for statistical significance. NES (normalized enrichment score). Significance of these pathways in P0 and P2 MFP tumors versus corresponding lung metastases is shown for comparison (right two columns).

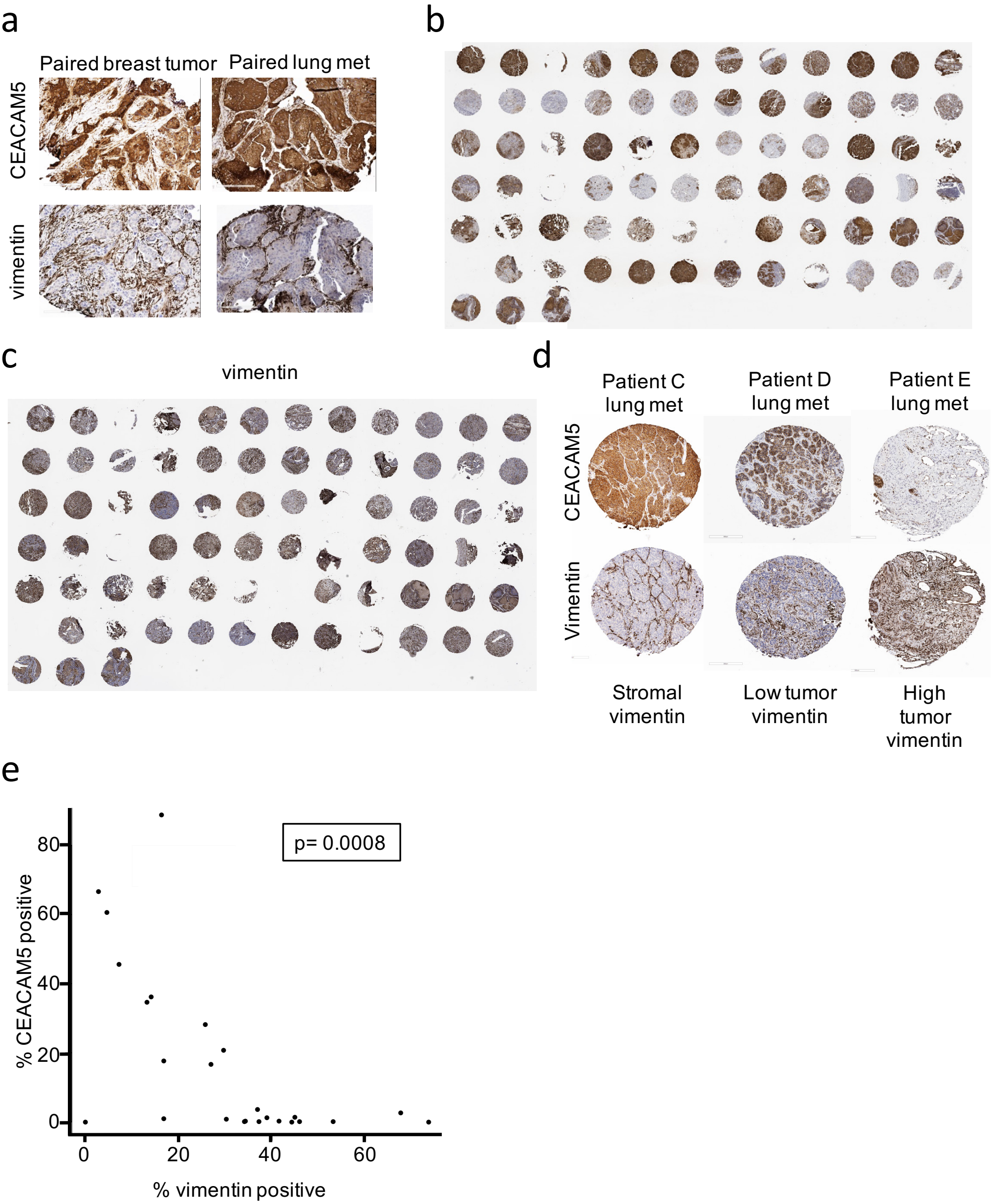

Supplementary Figure 12. Inverse correlation between CEACAM5 and vimentin in patient tumors. (a) The primary tumor and corresponding lung metastasis from a patient with breast cancer were subjected to IHC to monitor CEACAM5 and vimentin protein levels. (b and c) A tumor tissue microarray (TMA) consisting of lung metastases from 25 breast cancer patients was subjected to IHC with a CEACAM5-specific antibody (b) or a vimentin-specific antibody (c). (d) Larger magnifications of representative stained lung metastases from TMA shown in panels b and c. (e) The TMA of lung metastases in Fig. 7 was stained for CEACAM5 and vimentin, and percent positivity was quantified using the Vectra system. Spearman rank correlation, p= 0.0008067.

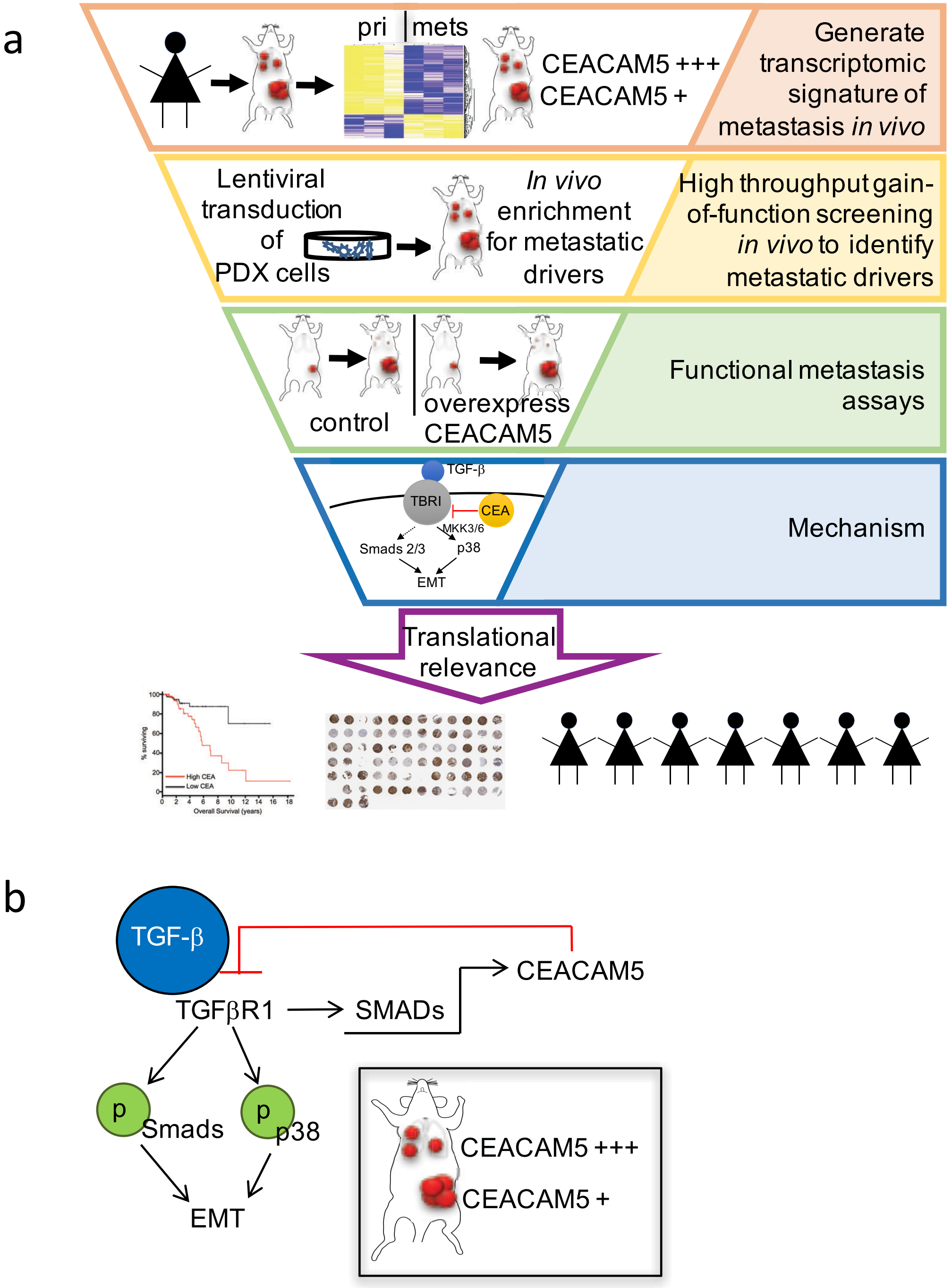

Supplementary Figure 13. Schematic representation of study design and conclusions. (a) Graphical representation of study design. (b) Model for mechanism by which CEACAM5 inhibits signaling pathways leading to EMT to promote tumor outgrowth in metastatic sites.
