# Supplementary material for: A functional genomic screen in vivo identifies CEACAM5 as a clinically relevant driver of breast cancer metastasis
Source: NPJ Breast Cancer. 2018 Apr 30;4:9. doi: 10.1038/s41523-018-0062-x (PMC5928229; doi:10.1038/s41523-018-0062-x)
Supplement: Supplementary file 2 — Supplementary Table 1 [file 41523_2018_62_MOESM2_ESM.docx]

| Supplementary Table 1 | |  |  |  |  |  |
| --- | --- | --- | --- | --- | --- | --- |
| **P0 lung metastasis signature:** | | |  |  |  |  |
| **Gene** | **baseMean** | **log2 Fold Change** | **lfcSE** | **stat** | **pvalue** | **padj** |
| NEU4 | 261.745 | 3.12 | 0.6077 | 5.1277 | 2.93E-07 | 0.00021 |
| DPEP1 | 841.309 | 3.05 | 0.4712 | 6.46992 | 9.81E-11 | 8.15E-07 |
| KRT13 | 174.509 | 2.91 | 0.6199 | 4.6897 | 2.74E-06 | 0.00106 |
| CACNA1C | 18.5196 | 2.76 | 0.59499 | 4.63466 | 3.58E-06 | 0.00126 |
| PPP1R14D | 53.6854 | 2.75 | 0.481 | 5.72651 | 1.03E-08 | 2.13E-05 |
| FXYD4 | 220.669 | 2.73 | 0.62416 | 4.38125 | 1.18E-05 | 0.00293 |
| TNFRSF1B | 190.354 | 2.73 | 0.51631 | 5.28555 | 1.25E-07 | 0.00012 |
| PSCA | 1139.38 | 2.71 | 0.56169 | 4.8333 | 1.34E-06 | 0.0007 |
| RAB37 | 394.091 | 2.71 | 0.55642 | 4.87174 | 1.11E-06 | 0.00059 |
| DIO3OS | 199.075 | 2.64 | 0.59088 | 4.4639 | 8.05E-06 | 0.00222 |
| FABP1 | 103.524 | 2.62 | 0.6414 | 4.0881 | 4.35E-05 | 0.00812 |
| RXFP4 | 19.4374 | 2.61 | 0.5449 | 4.78581 | 1.70E-06 | 0.00081 |
| LINC01342 | 8.86361 | 2.56 | 0.61313 | 4.17531 | 2.98E-05 | 0.00618 |
| SLC12A3 | 40.0974 | 2.55 | 0.63152 | 4.04041 | 5.34E-05 | 0.00944 |
| CREB3L1 | 784.953 | 2.54 | 0.53372 | 4.75154 | 2.02E-06 | 0.00088 |
| TFF1 | 1864.76 | 2.54 | 0.55526 | 4.56657 | 4.96E-06 | 0.00156 |
| SPNS2 | 168.915 | 2.52 | 0.49459 | 5.09389 | 3.51E-07 | 0.00024 |
| KLK13 | 17.4768 | 2.50 | 0.64064 | 3.90456 | 9.44E-05 | 0.01414 |
| LGALS9C | 26.276 | 2.49 | 0.63039 | 3.95768 | 7.57E-05 | 0.01198 |
| SLC51B | 188.94 | 2.49 | 0.5023 | 4.96 | 7.05E-07 | 0.00045 |
| RBBP8NL | 91.6983 | 2.49 | 0.5561 | 4.47778 | 7.54E-06 | 0.00213 |
| CREB3L3 | 11.3545 | 2.46 | 0.62572 | 3.93943 | 8.17E-05 | 0.01281 |
| C8G | 103.735 | 2.46 | 0.61082 | 4.02079 | 5.80E-05 | 0.01004 |
| MUC5AC | 11695.4 | 2.45 | 0.51488 | 4.76762 | 1.86E-06 | 0.00084 |
| ALDOB | 269.273 | 2.45 | 0.56508 | 4.33927 | 1.43E-05 | 0.0035 |
| CD22 | 60.8822 | 2.45 | 0.55845 | 4.38307 | 1.17E-05 | 0.00293 |
| GBGT1 | 87.0682 | 2.43 | 0.49485 | 4.91937 | 8.68E-07 | 0.0005 |
| PRSS36 | 46.2055 | 2.43 | 0.59039 | 4.12008 | 3.79E-05 | 0.00723 |
| PHGR1 | 733.814 | 2.41 | 0.59513 | 4.05573 | 5.00E-05 | 0.00903 |
| MIR192 | 9.83579 | 2.40 | 0.60302 | 3.97663 | 6.99E-05 | 0.01151 |
| PRAP1 | 2204.85 | 2.38 | 0.5957 | 3.99729 | 6.41E-05 | 0.01076 |
| MUC5B | 7932.8 | 2.35 | 0.47716 | 4.92529 | 8.42E-07 | 0.0005 |
| PTH1R | 40.4586 | 2.32 | 0.54398 | 4.27401 | 1.92E-05 | 0.00437 |
| TFF3 | 3370.02 | 2.32 | 0.65135 | 3.5639 | 0.00037 | 0.03797 |
| TMEM82 | 40.0278 | 2.32 | 0.63538 | 3.64372 | 0.00027 | 0.03041 |
| TPO | 9.73318 | 2.31 | 0.63803 | 3.6213 | 0.00029 | 0.03267 |
| AMN | 1099.84 | 2.30 | 0.5143 | 4.47862 | 7.51E-06 | 0.00213 |
| FXYD3 | 1957.55 | 2.28 | 0.58491 | 3.89617 | 9.77E-05 | 0.01451 |
| MAMDC4 | 562.603 | 2.27 | 0.49794 | 4.56565 | 4.98E-06 | 0.00156 |
| GUCA2A | 319.415 | 2.27 | 0.47605 | 4.76904 | 1.85E-06 | 0.00084 |
| RORC | 202.577 | 2.27 | 0.56666 | 4.00337 | 6.24E-05 | 0.01059 |
| SLC17A4 | 31.0901 | 2.27 | 0.4908 | 4.61862 | 3.86E-06 | 0.00131 |
| HS3ST6 | 18.2849 | 2.27 | 0.64752 | 3.49943 | 0.00047 | 0.04481 |
| DLL4 | 27.5235 | 2.26 | 0.50177 | 4.51198 | 6.42E-06 | 0.00198 |
| SMOC1 | 290.365 | 2.25 | 0.57278 | 3.93001 | 8.49E-05 | 0.0132 |
| ADAMTS2 | 184.403 | 2.23 | 0.53422 | 4.1827 | 2.88E-05 | 0.00606 |
| FXYD6 | 58.571 | 2.23 | 0.64156 | 3.47999 | 0.0005 | 0.04683 |
| SSTR5 | 80.8536 | 2.23 | 0.6204 | 3.59074 | 0.00033 | 0.0356 |
| CCR10 | 16.0865 | 2.21 | 0.63883 | 3.46454 | 0.00053 | 0.04768 |
| SCARA5 | 95.4992 | 2.21 | 0.44831 | 4.9332 | 8.09E-07 | 0.0005 |
| LDLRAD1 | 166.227 | 2.20 | 0.39778 | 5.53173 | 3.17E-08 | 4.30E-05 |
| GAL3ST2 | 50.6837 | 2.19 | 0.57622 | 3.79906 | 0.00015 | 0.01979 |
| MYL3 | 19.28 | 2.18 | 0.52367 | 4.15702 | 3.22E-05 | 0.00654 |
| TRPV6 | 3.72212 | 2.17 | 0.64894 | 3.34992 | 0.00081 | 0.0628 |
| HSD11B2 | 85.1563 | 2.15 | 0.63543 | 3.38898 | 0.0007 | 0.05794 |
| VTN | 146.854 | 2.15 | 0.63449 | 3.39251 | 0.00069 | 0.05757 |
| LOC729966 | 13.9552 | 2.15 | 0.5718 | 3.75924 | 0.00017 | 0.0221 |
| HOXC5 | 3.95556 | 2.14 | 0.65469 | 3.2738 | 0.00106 | 0.07464 |
| ALPI | 176.058 | 2.12 | 0.57324 | 3.70611 | 0.00021 | 0.02554 |
| SLC22A11 | 13.4794 | 2.12 | 0.55407 | 3.82812 | 0.00013 | 0.01835 |
| AATK | 1030.59 | 2.11 | 0.4455 | 4.7402 | 2.14E-06 | 0.00091 |
| UNC5B | 107.536 | 2.11 | 0.60957 | 3.46285 | 0.00053 | 0.04768 |
| KLK1 | 16.1974 | 2.10 | 0.64766 | 3.2469 | 0.00117 | 0.07862 |
| F7 | 56.1572 | 2.10 | 0.62969 | 3.3357 | 0.00085 | 0.06489 |
| BDKRB2 | 359.913 | 2.09 | 0.4448 | 4.69791 | 2.63E-06 | 0.00106 |
| PPP1R16B | 9.96029 | 2.07 | 0.60514 | 3.42362 | 0.00062 | 0.05323 |
| ZNF703 | 213.247 | 2.07 | 0.59906 | 3.45779 | 0.00054 | 0.04816 |
| ANPEP | 1111.38 | 2.07 | 0.577 | 3.58396 | 0.00034 | 0.03607 |
| TM4SF5 | 324.055 | 2.07 | 0.60124 | 3.4392 | 0.00058 | 0.05128 |
| CATIP | 7.06976 | 2.07 | 0.64107 | 3.22239 | 0.00127 | 0.08329 |
| GLTPD2 | 162.511 | 2.05 | 0.57905 | 3.5463 | 0.00039 | 0.0396 |
| DEGS2 | 260.54 | 2.05 | 0.61293 | 3.34465 | 0.00082 | 0.06371 |
| MUC20 | 198.091 | 2.05 | 0.55019 | 3.71866 | 0.0002 | 0.02466 |
| PRR22 | 21.8207 | 2.03 | 0.58032 | 3.50629 | 0.00045 | 0.04392 |
| SFTA2 | 111.223 | 2.03 | 0.56561 | 3.59269 | 0.00033 | 0.0356 |
| LOC100133669 | 7.88463 | 2.02 | 0.63936 | 3.16286 | 0.00156 | 0.09566 |
| GJB1 | 469.189 | 2.02 | 0.63439 | 3.18559 | 0.00144 | 0.09097 |
| CD82 | 322.684 | 2.01 | 0.4878 | 4.12708 | 3.67E-05 | 0.0071 |
| ARX | 7.37667 | 2.01 | 0.61936 | 3.24572 | 0.00117 | 0.07862 |
| CCDC108 | 8.67598 | 2.01 | 0.5961 | 3.36896 | 0.00075 | 0.06031 |
| HMCN2 | 28.4787 | 2.01 | 0.5407 | 3.7098 | 0.00021 | 0.02536 |
| SLCO4A1-AS1 | 35.6121 | 1.99 | 0.58545 | 3.39436 | 0.00069 | 0.05747 |
| LCN12 | 13.3836 | 1.99 | 0.61181 | 3.24704 | 0.00117 | 0.07862 |
| SSPO | 25.8128 | 1.98 | 0.61929 | 3.2029 | 0.00136 | 0.08666 |
| TMPRSS6 | 85.5405 | 1.98 | 0.54267 | 3.65353 | 0.00026 | 0.02986 |
| ERN2 | 310.934 | 1.98 | 0.5952 | 3.32191 | 0.00089 | 0.06726 |
| MGAT3 | 208.309 | 1.97 | 0.47825 | 4.12755 | 3.67E-05 | 0.0071 |
| RCN3 | 43.7653 | 1.97 | 0.5668 | 3.47137 | 0.00052 | 0.04704 |
| INHBB | 411.021 | 1.97 | 0.55919 | 3.51535 | 0.00044 | 0.04346 |
| FAM83E | 454.448 | 1.96 | 0.54283 | 3.61986 | 0.00029 | 0.03267 |
| MSMB | 27.476 | 1.96 | 0.55002 | 3.56006 | 0.00037 | 0.03814 |
| STARD8 | 146.36 | 1.96 | 0.41766 | 4.68103 | 2.85E-06 | 0.00106 |
| HPN | 1040.55 | 1.95 | 0.6057 | 3.22209 | 0.00127 | 0.08329 |
| IHH | 10.858 | 1.95 | 0.58658 | 3.32495 | 0.00088 | 0.06683 |
| LOC115110 | 70.7351 | 1.95 | 0.54854 | 3.5538 | 0.00038 | 0.03873 |
| GDPD2 | 38.115 | 1.94 | 0.45181 | 4.29556 | 1.74E-05 | 0.00402 |
| NPDC1 | 236.336 | 1.94 | 0.58469 | 3.30999 | 0.00093 | 0.06925 |
| SCN8A | 159.769 | 1.94 | 0.54082 | 3.57839 | 0.00035 | 0.03658 |
| SHH | 45.6782 | 1.93 | 0.52877 | 3.65689 | 0.00026 | 0.02986 |
| MYO1A | 580.96 | 1.93 | 0.52328 | 3.68639 | 0.00023 | 0.0272 |
| GPR55 | 8.35386 | 1.92 | 0.58548 | 3.28278 | 0.00103 | 0.07334 |
| GDF15 | 337.81 | 1.92 | 0.57973 | 3.30661 | 0.00094 | 0.06947 |
| PTPRN2 | 81.9467 | 1.91 | 0.58881 | 3.25094 | 0.00115 | 0.07862 |
| ADGRD1 | 28.5683 | 1.91 | 0.56268 | 3.38804 | 0.0007 | 0.05794 |
| CYP2S1 | 477.848 | 1.91 | 0.48156 | 3.95877 | 7.53E-05 | 0.01198 |
| GPT | 61.6269 | 1.89 | 0.50388 | 3.75847 | 0.00017 | 0.0221 |
| SLCO4A1 | 151.706 | 1.89 | 0.55258 | 3.42515 | 0.00061 | 0.0532 |
| BMP2 | 19.2111 | 1.89 | 0.55508 | 3.4088 | 0.00065 | 0.05479 |
| PDZD3 | 191.802 | 1.89 | 0.51465 | 3.67122 | 0.00024 | 0.02867 |
| SCIN | 50.6416 | 1.88 | 0.52008 | 3.61325 | 0.0003 | 0.03329 |
| BEGAIN | 37.2528 | 1.88 | 0.55417 | 3.38367 | 0.00072 | 0.05858 |
| ADGRG5 | 67.087 | 1.87 | 0.54675 | 3.41636 | 0.00063 | 0.05411 |
| CAPN9 | 473.173 | 1.86 | 0.4127 | 4.49478 | 6.96E-06 | 0.00207 |
| CA9 | 195.804 | 1.85 | 0.52851 | 3.49189 | 0.00048 | 0.04563 |
| SLC47A2 | 111.668 | 1.85 | 0.52936 | 3.48621 | 0.00049 | 0.04628 |
| DOK7 | 98.9645 | 1.84 | 0.44533 | 4.14058 | 3.46E-05 | 0.00686 |
| SLC39A5 | 527.675 | 1.84 | 0.55203 | 3.33956 | 0.00084 | 0.06429 |
| ATP2A3 | 1693.46 | 1.83 | 0.53477 | 3.41931 | 0.00063 | 0.0538 |
| ENTPD8 | 205.102 | 1.83 | 0.46691 | 3.90934 | 9.25E-05 | 0.01399 |
| NPAS1 | 44.3054 | 1.81 | 0.55918 | 3.23668 | 0.00121 | 0.08059 |
| SLC17A9 | 613.088 | 1.81 | 0.56308 | 3.2122 | 0.00132 | 0.08455 |
| CLDN15 | 147.389 | 1.80 | 0.44302 | 4.07423 | 4.62E-05 | 0.00847 |
| PRR15L | 651.91 | 1.77 | 0.45225 | 3.91349 | 9.10E-05 | 0.01388 |
| SPOCD1 | 18.0005 | 1.76 | 0.53664 | 3.28385 | 0.00102 | 0.07334 |
| KCP | 678.484 | 1.76 | 0.45147 | 3.88899 | 0.0001 | 0.0147 |
| LOC100130705 | 220.531 | 1.75 | 0.43406 | 4.03598 | 5.44E-05 | 0.00952 |
| ZMYND15 | 58.2539 | 1.75 | 0.51129 | 3.42624 | 0.00061 | 0.0532 |
| TST | 409.058 | 1.75 | 0.55394 | 3.15022 | 0.00163 | 0.09775 |
| HABP2 | 11.6084 | 1.74 | 0.52727 | 3.30033 | 0.00097 | 0.07011 |
| ADAP1 | 775.039 | 1.71 | 0.454 | 3.77388 | 0.00016 | 0.02145 |
| SLCO2B1 | 772.372 | 1.70 | 0.4108 | 4.14214 | 3.44E-05 | 0.00686 |
| SMIM5 | 94.5575 | 1.68 | 0.5168 | 3.2454 | 0.00117 | 0.07862 |
| RAB40B | 66.3347 | 1.67 | 0.50773 | 3.29334 | 0.00099 | 0.07125 |
| ACSS1 | 288.094 | 1.66 | 0.51464 | 3.23425 | 0.00122 | 0.08078 |
| IRX4 | 58.0754 | 1.65 | 0.52604 | 3.14571 | 0.00166 | 0.09837 |
| SPIRE2 | 201.987 | 1.64 | 0.52185 | 3.14787 | 0.00164 | 0.098 |
| CACNA1H | 559.604 | 1.63 | 0.50689 | 3.22036 | 0.00128 | 0.08333 |
| F10 | 109.379 | 1.63 | 0.49378 | 3.30517 | 0.00095 | 0.06952 |
| CEACAM5 | 624.968 | 1.63 | 0.50247 | 3.24729 | 0.00117 | 0.07862 |
| SFI1 | 330.863 | 1.60 | 0.43001 | 3.73083 | 0.00019 | 0.02386 |
| VWA7 | 159.732 | 1.60 | 0.42801 | 3.73951 | 0.00018 | 0.02322 |
| VILL | 736.855 | 1.59 | 0.47201 | 3.36982 | 0.00075 | 0.06031 |
| ZNF497 | 46.0895 | 1.59 | 0.4859 | 3.27344 | 0.00106 | 0.07464 |
| LOC101928738 | 78.181 | 1.57 | 0.48335 | 3.25532 | 0.00113 | 0.07862 |
| KCNK5 | 582.139 | 1.57 | 0.47094 | 3.34053 | 0.00084 | 0.06429 |
| TRIM15 | 75.4641 | 1.57 | 0.49418 | 3.16995 | 0.00152 | 0.09517 |
| SMPD3 | 203.981 | 1.57 | 0.49454 | 3.16539 | 0.00155 | 0.09566 |
| PROC | 56.3492 | 1.56 | 0.46442 | 3.35967 | 0.00078 | 0.06119 |
| KLK11 | 1123.32 | 1.56 | 0.47471 | 3.27884 | 0.00104 | 0.07406 |
| UPK1B | 1390.69 | 1.53 | 0.47997 | 3.19468 | 0.0014 | 0.08883 |
| PRSS22 | 94.2426 | 1.51 | 0.38212 | 3.96375 | 7.38E-05 | 0.01198 |
| SPEG | 206.967 | 1.49 | 0.46403 | 3.20715 | 0.00134 | 0.08572 |
| CAPN8 | 839.88 | 1.45 | 0.44604 | 3.24712 | 0.00117 | 0.07862 |
| SH3PXD2A | 101.265 | 1.44 | 0.41147 | 3.5072 | 0.00045 | 0.04392 |
| ADORA2B | 147.523 | 1.43 | 0.4547 | 3.14967 | 0.00163 | 0.09775 |
| NPB | 34.0368 | 1.43 | 0.45085 | 3.16176 | 0.00157 | 0.09566 |
| PNPLA1 | 49.6723 | 1.38 | 0.42594 | 3.23605 | 0.00121 | 0.08059 |
| MRGPRX3 | 102.509 | 1.29 | 0.40162 | 3.21954 | 0.00128 | 0.08333 |
| AKR7L | 132.085 | 1.25 | 0.37065 | 3.38089 | 0.00072 | 0.05888 |
| B3GNT3 | 661.405 | 1.25 | 0.35049 | 3.55941 | 0.00037 | 0.03814 |
| SLC25A25 | 609.245 | 1.20 | 0.35025 | 3.41268 | 0.00064 | 0.05448 |
| ERBB3 | 1098.94 | 1.15 | 0.33125 | 3.47733 | 0.00051 | 0.04689 |
| TACC2 | 684.39 | 0.94 | 0.2992 | 3.15318 | 0.00162 | 0.09775 |
| NREP | 76.3816 | -1.25 | 0.37258 | -3.3663 | 0.00076 | 0.0606 |
| MSN | 1365.31 | -1.28 | 0.40256 | -3.1795 | 0.00148 | 0.09255 |
| PRSS23 | 2036.54 | -1.28 | 0.39956 | -3.2139 | 0.00131 | 0.08437 |
| KIRREL | 184.983 | -1.30 | 0.3928 | -3.3113 | 0.00093 | 0.06922 |
| MSRB3 | 162.863 | -1.32 | 0.41082 | -3.2246 | 0.00126 | 0.08322 |
| CHST3 | 1118.15 | -1.35 | 0.39952 | -3.3695 | 0.00075 | 0.06031 |
| FLNA | 14960.2 | -1.42 | 0.42932 | -3.3136 | 0.00092 | 0.06898 |
| TUFT1 | 863.094 | -1.42 | 0.40355 | -3.5274 | 0.00042 | 0.04202 |
| GPR1 | 35.0638 | -1.43 | 0.45375 | -3.1497 | 0.00163 | 0.09775 |
| CAP1 | 1292.1 | -1.48 | 0.38919 | -3.813 | 0.00014 | 0.01902 |
| OSMR | 624.611 | -1.50 | 0.46535 | -3.2186 | 0.00129 | 0.08333 |
| NEDD9 | 314.447 | -1.51 | 0.4598 | -3.2934 | 0.00099 | 0.07125 |
| IFFO2 | 91.1083 | -1.52 | 0.45317 | -3.3634 | 0.00077 | 0.06084 |
| NUAK1 | 177.76 | -1.53 | 0.42486 | -3.5916 | 0.00033 | 0.0356 |
| SCPEP1 | 59.0404 | -1.55 | 0.40844 | -3.7952 | 0.00015 | 0.01994 |
| BHLHE40 | 540.183 | -1.57 | 0.3632 | -4.3279 | 1.51E-05 | 0.00363 |
| PTRF | 839.47 | -1.58 | 0.33697 | -4.681 | 2.85E-06 | 0.00106 |
| FAM171A1 | 93.9657 | -1.58 | 0.46056 | -3.438 | 0.00059 | 0.05128 |
| PDGFB | 125.527 | -1.61 | 0.44131 | -3.6378 | 0.00027 | 0.03089 |
| TPM1 | 1483.46 | -1.61 | 0.38199 | -4.2145 | 2.50E-05 | 0.0054 |
| MCAM | 206.463 | -1.61 | 0.4812 | -3.3543 | 0.0008 | 0.06209 |
| LHFP | 33.7713 | -1.61 | 0.51221 | -3.1528 | 0.00162 | 0.09775 |
| CTGF | 405.275 | -1.62 | 0.4511 | -3.5855 | 0.00034 | 0.03607 |
| TUBB3 | 694.942 | -1.64 | 0.5035 | -3.2668 | 0.00109 | 0.07599 |
| ADAMTS1 | 254.717 | -1.67 | 0.50674 | -3.3004 | 0.00097 | 0.07011 |
| CTSV | 74.9074 | -1.70 | 0.40072 | -4.2376 | 2.26E-05 | 0.00494 |
| ZBED2 | 275.414 | -1.71 | 0.44848 | -3.8052 | 0.00014 | 0.01947 |
| CYR61 | 159.438 | -1.72 | 0.49447 | -3.4737 | 0.00051 | 0.04689 |
| CALD1 | 867.453 | -1.73 | 0.41613 | -4.1576 | 3.22E-05 | 0.00654 |
| CHST11 | 208.952 | -1.73 | 0.47458 | -3.655 | 0.00026 | 0.02986 |
| AMIGO2 | 1336.46 | -1.77 | 0.44148 | -4.0067 | 6.16E-05 | 0.01055 |
| MLLT11 | 157.053 | -1.80 | 0.47921 | -3.762 | 0.00017 | 0.0221 |
| RAB31 | 93.5456 | -1.81 | 0.52214 | -3.4689 | 0.00052 | 0.04722 |
| PYGL | 701.131 | -1.82 | 0.39212 | -4.6291 | 3.67E-06 | 0.00127 |
| DKK3 | 120.758 | -1.83 | 0.355 | -5.1615 | 2.45E-07 | 0.00019 |
| TNFRSF19 | 20.6076 | -1.83 | 0.56029 | -3.273 | 0.00106 | 0.07464 |
| COL4A2 | 4385.01 | -1.84 | 0.52512 | -3.5108 | 0.00045 | 0.04392 |
| NNMT | 527.935 | -1.85 | 0.56 | -3.3067 | 0.00094 | 0.06947 |
| LCP1 | 27.205 | -1.86 | 0.54501 | -3.4117 | 0.00065 | 0.05448 |
| DSE | 60.7451 | -1.89 | 0.54437 | -3.4806 | 0.0005 | 0.04683 |
| NKILA | 27.6171 | -1.91 | 0.54424 | -3.5073 | 0.00045 | 0.04392 |
| PMEPA1 | 2173.88 | -1.91 | 0.4687 | -4.0732 | 4.64E-05 | 0.00847 |
| LTBP1 | 119.614 | -1.91 | 0.5096 | -3.7576 | 0.00017 | 0.0221 |
| CTPS1 | 275.654 | -1.92 | 0.47602 | -4.0411 | 5.32E-05 | 0.00944 |
| LBH | 681.008 | -1.94 | 0.5075 | -3.8257 | 0.00013 | 0.01837 |
| ADAMTS15 | 54.3377 | -1.95 | 0.61781 | -3.1629 | 0.00156 | 0.09566 |
| TUBA1A | 71.0137 | -1.97 | 0.52231 | -3.773 | 0.00016 | 0.02145 |
| TRIML2 | 66.9824 | -1.97 | 0.52963 | -3.7222 | 0.0002 | 0.0245 |
| C1S | 135.685 | -1.97 | 0.51615 | -3.8237 | 0.00013 | 0.01837 |
| AOX1 | 23.7613 | -1.98 | 0.58598 | -3.3734 | 0.00074 | 0.0602 |
| GLIPR1 | 37.6468 | -1.98 | 0.58814 | -3.3626 | 0.00077 | 0.06084 |
| CASC15 | 58.8501 | -1.98 | 0.52733 | -3.7534 | 0.00017 | 0.02231 |
| ROCK1P1 | 6.78857 | -1.99 | 0.6223 | -3.1899 | 0.00142 | 0.08995 |
| COL5A1 | 521.908 | -1.99 | 0.62863 | -3.1654 | 0.00155 | 0.09566 |
| IGFBP7 | 45.8496 | -2.00 | 0.63196 | -3.1613 | 0.00157 | 0.09566 |
| ANKRD1 | 101.228 | -2.00 | 0.37164 | -5.3876 | 7.14E-08 | 7.86E-05 |
| PHLDB2 | 200.68 | -2.04 | 0.57195 | -3.5753 | 0.00035 | 0.03658 |
| DPYSL3 | 161.587 | -2.05 | 0.64549 | -3.1692 | 0.00153 | 0.09517 |
| FERMT2 | 208.581 | -2.06 | 0.48407 | -4.2579 | 2.06E-05 | 0.00457 |
| TFPI2 | 88.2583 | -2.07 | 0.39496 | -5.2378 | 1.63E-07 | 0.00014 |
| SERPINE1 | 1022.1 | -2.07 | 0.53699 | -3.8637 | 0.00011 | 0.016 |
| ETS1 | 212.368 | -2.08 | 0.49653 | -4.1893 | 2.80E-05 | 0.00597 |
| SEMA7A | 208.858 | -2.09 | 0.60092 | -3.4752 | 0.00051 | 0.04689 |
| PXDNL | 20.6173 | -2.09 | 0.52347 | -3.9922 | 6.55E-05 | 0.01088 |
| BEAN1 | 25.0933 | -2.09 | 0.55945 | -3.7418 | 0.00018 | 0.02319 |
| NIPAL4 | 4.50927 | -2.11 | 0.64911 | -3.2498 | 0.00115 | 0.07862 |
| LOXL2 | 121.294 | -2.11 | 0.57801 | -3.6499 | 0.00026 | 0.03008 |
| COL4A1 | 2054.49 | -2.12 | 0.59813 | -3.5425 | 0.0004 | 0.03994 |
| BST1 | 12.0756 | -2.12 | 0.60728 | -3.4915 | 0.00048 | 0.04563 |
| SLCO2A1 | 37.3946 | -2.13 | 0.65572 | -3.2489 | 0.00116 | 0.07862 |
| LINC00862 | 15.3481 | -2.15 | 0.59082 | -3.6435 | 0.00027 | 0.03041 |
| ZEB2 | 7.18906 | -2.17 | 0.65004 | -3.3311 | 0.00087 | 0.06568 |
| MAFB | 14.8497 | -2.18 | 0.59305 | -3.6684 | 0.00024 | 0.02878 |
| BDNF | 53.3058 | -2.19 | 0.55402 | -3.9604 | 7.48E-05 | 0.01198 |
| INHBA | 41.5954 | -2.20 | 0.56809 | -3.8641 | 0.00011 | 0.016 |
| CCL2 | 18.1801 | -2.22 | 0.63852 | -3.4752 | 0.00051 | 0.04689 |
| CDH19 | 8.5672 | -2.27 | 0.6555 | -3.4619 | 0.00054 | 0.04768 |
| QPCT | 11.7012 | -2.27 | 0.63541 | -3.5754 | 0.00035 | 0.03658 |
| COL8A1 | 19.2051 | -2.27 | 0.64498 | -3.5224 | 0.00043 | 0.04258 |
| ARHGAP28 | 38.9458 | -2.32 | 0.62783 | -3.6967 | 0.00022 | 0.02631 |
| CAV1 | 470.068 | -2.33 | 0.43154 | -5.3918 | 6.98E-08 | 7.86E-05 |
| RBP1 | 391.701 | -2.35 | 0.60546 | -3.8886 | 0.0001 | 0.0147 |
| LTBP2 | 470.533 | -2.37 | 0.52634 | -4.5056 | 6.62E-06 | 0.002 |
| GFRA1 | 30.0334 | -2.43 | 0.62171 | -3.9151 | 9.04E-05 | 0.01388 |
| HEG1 | 99.838 | -2.46 | 0.51428 | -4.7895 | 1.67E-06 | 0.00081 |
| VIM | 1795.62 | -2.47 | 0.57078 | -4.3247 | 1.53E-05 | 0.00363 |
| CDH2 | 47.6039 | -2.49 | 0.56449 | -4.4063 | 1.05E-05 | 0.00275 |
| KRT6A | 2291.55 | -2.50 | 0.58103 | -4.3025 | 1.69E-05 | 0.00395 |
| THBS1 | 1876.77 | -2.55 | 0.58042 | -4.3997 | 1.08E-05 | 0.00277 |
| SLC2A3 | 514.795 | -2.56 | 0.35419 | -7.2209 | 5.16E-13 | 8.59E-09 |
| GFPT2 | 18.7275 | -2.56 | 0.58153 | -4.4047 | 1.06E-05 | 0.00275 |
| MMP2 | 104.745 | -2.56 | 0.57485 | -4.4609 | 8.16E-06 | 0.00222 |
| MARCH4        . | 44.2447 | -2.57 | 0.57919 | -4.4392 | 9.03E-06 | 0.00242 |
| TNC | 33.4172 | -2.59 | 0.62795 | -4.1177 | 3.83E-05 | 0.00723 |
| FHOD3 | 27.7108 | -2.59 | 0.57784 | -4.4788 | 7.51E-06 | 0.00213 |
| CACNG4 | 57.6615 | -2.60 | 0.5006 | -5.1865 | 2.14E-07 | 0.00017 |
| TAGLN | 1608.48 | -2.61 | 0.54325 | -4.797 | 1.61E-06 | 0.00081 |
| DAW1 | 15.0873 | -2.61 | 0.55856 | -4.6806 | 2.86E-06 | 0.00106 |
| CCDC80 | 91.415 | -2.65 | 0.54099 | -4.8997 | 9.60E-07 | 0.00053 |
| EPHB2 | 218.377 | -2.70 | 0.47997 | -5.6196 | 1.91E-08 | 3.40E-05 |
| CNN3 | 129.67 | -2.70 | 0.48304 | -5.5918 | 2.25E-08 | 3.40E-05 |
| ADAM19 | 28.3651 | -2.71 | 0.57352 | -4.7315 | 2.23E-06 | 0.00093 |
| KRT14 | 37.6445 | -2.72 | 0.63768 | -4.2713 | 1.94E-05 | 0.00437 |
| WNT11 | 202.116 | -2.73 | 0.49393 | -5.5215 | 3.36E-08 | 4.30E-05 |
| SPARC | 49.0717 | -2.73 | 0.58796 | -4.6447 | 3.41E-06 | 0.00123 |
| PLXDC2 | 148.669 | -2.73 | 0.5174 | -5.2845 | 1.26E-07 | 0.00012 |
| NRCAM | 12.0799 | -2.75 | 0.60012 | -4.5897 | 4.44E-06 | 0.00145 |
| CD44 | 153.664 | -2.80 | 0.55888 | -5.004 | 5.62E-07 | 0.00037 |
| KRT17 | 11953.2 | -2.81 | 0.52268 | -5.3772 | 7.57E-08 | 7.86E-05 |
| TSPAN2 | 10.3837 | -2.86 | 0.6223 | -4.6003 | 4.22E-06 | 0.0014 |
| SERPINA3 | 463.783 | -2.93 | 0.55517 | -5.2698 | 1.37E-07 | 0.00012 |
| CYP24A1 | 507.651 | -2.93 | 0.49282 | -5.9432 | 2.80E-09 | 6.64E-06 |
| TGFBI | 2314.92 | -2.97 | 0.49339 | -6.0101 | 1.85E-09 | 5.14E-06 |
| ANTXR1 | 32.7332 | -3.17 | 0.51824 | -6.1072 | 1.01E-09 | 4.21E-06 |
| FSTL1 | 304.507 | -3.21 | 0.53165 | -6.0462 | 1.48E-09 | 4.93E-06 |
| TGFB2 | 100.438 | -3.33 | 0.52442 | -6.3445 | 2.23E-10 | 1.24E-06 |
| C5orf46 | 15.4214 | -3.47 | 0.61952 | -5.6049 | 2.08E-08 | 3.40E-05 |
|  |  |  |  |  |  |  |
| **P2 lung metastasis signature** | | |  |  |  |  |
| **Gene** | **baseMean** | **log2 Fold Change** | **lfcSE** | **stat** | **pvalue** |  |
| PSMB9 | 811.761 | 2.80 | 0.26017 | 10.7741 | 4.56E-27 |  |
| UNC5B | 222.82 | 2.67 | 0.2749 | 9.69792 | 3.08E-22 |  |
| IFITM1 | 339.355 | 2.62 | 0.36469 | 7.17776 | 7.09E-13 |  |
| TNFRSF1B | 416.271 | 2.58 | 0.17716 | 14.5442 | 6.35E-48 |  |
| DHRS2 | 251.629 | 2.52 | 0.24495 | 10.2817 | 8.52E-25 |  |
| IFITM3 | 1614.96 | 2.46 | 0.27747 | 8.86953 | 7.35E-19 |  |
| IFI44 | 519.232 | 2.41 | 0.3128 | 7.69463 | 1.42E-14 |  |
| RTP4 | 47.9591 | 2.21 | 0.34683 | 6.36095 | 2.01E-10 |  |
| CMPK2 | 872.635 | 2.20 | 0.31643 | 6.96321 | 3.33E-12 |  |
| SPTSSB | 41.3424 | 2.16 | 0.34726 | 6.2079 | 5.37E-10 |  |
| UBE2L6 | 547.503 | 2.12 | 0.22236 | 9.51612 | 1.80E-21 |  |
| IFIT3 | 1331.94 | 2.09 | 0.27621 | 7.56423 | 3.90E-14 |  |
| BST2 | 2316.01 | 2.04 | 0.16563 | 12.31 | 8.00E-35 |  |
| HERC5 | 220.642 | 2.02 | 0.33808 | 5.97982 | 2.23E-09 |  |
| HLA-B | 15913.9 | 1.99 | 0.23438 | 8.48266 | 2.20E-17 |  |
| EPSTI1 | 827.535 | 1.98 | 0.328 | 6.04269 | 1.52E-09 |  |
| OASL | 578.039 | 1.91 | 0.33898 | 5.63142 | 1.79E-08 |  |
| PSMB8-AS1 | 220.868 | 1.90 | 0.23332 | 8.16031 | 3.34E-16 |  |
| GBGT1 | 339.192 | 1.88 | 0.29313 | 6.41398 | 1.42E-10 |  |
| HERC6 | 984.777 | 1.86 | 0.21784 | 8.54721 | 1.26E-17 |  |
| NCCRP1 | 1003.42 | 1.85 | 0.23225 | 7.95279 | 1.82E-15 |  |
| HCP5 | 1198.55 | 1.83 | 0.19613 | 9.33304 | 1.03E-20 |  |
| UBA7 | 99.0567 | 1.82 | 0.30517 | 5.96628 | 2.43E-09 |  |
| RSAD2 | 458.181 | 1.79 | 0.30936 | 5.77201 | 7.83E-09 |  |
| IFI44L | 956.091 | 1.74 | 0.39366 | 4.4275 | 9.53E-06 |  |
| ETV7 | 81.1211 | 1.73 | 0.37839 | 4.57006 | 4.88E-06 |  |
| HCG26 | 154.151 | 1.72 | 0.29507 | 5.83248 | 5.46E-09 |  |
| APOC3 | 20.8569 | 1.71 | 0.38664 | 4.43426 | 9.24E-06 |  |
| BTN3A3 | 337.959 | 1.71 | 0.23021 | 7.4147 | 1.22E-13 |  |
| KLK13 | 67.9636 | 1.69 | 0.2935 | 5.76938 | 7.96E-09 |  |
| IFI16 | 1584.55 | 1.69 | 0.29117 | 5.79504 | 6.83E-09 |  |
| HLA-F | 1715.88 | 1.67 | 0.24352 | 6.85432 | 7.17E-12 |  |
| LAMP3 | 902.081 | 1.66 | 0.31407 | 5.28124 | 1.28E-07 |  |
| C9orf152 | 1228.82 | 1.65 | 0.23589 | 6.99647 | 2.62E-12 |  |
| IFIT2 | 280.24 | 1.65 | 0.24413 | 6.75876 | 1.39E-11 |  |
| GLI3 | 42.2435 | 1.64 | 0.35689 | 4.60067 | 4.21E-06 |  |
| TEX19 | 76.0468 | 1.62 | 0.27118 | 5.98846 | 2.12E-09 |  |
| SLC22A11 | 164.726 | 1.62 | 0.21955 | 7.39229 | 1.44E-13 |  |
| BEST1 | 48.9914 | 1.60 | 0.32783 | 4.88148 | 1.05E-06 |  |
| BATF2 | 727.329 | 1.60 | 0.21768 | 7.34572 | 2.05E-13 |  |
| CKMT1B | 243.876 | 1.59 | 0.25083 | 6.35531 | 2.08E-10 |  |
| ANKRD20A19P | 36.0299 | 1.57 | 0.373 | 4.21061 | 2.55E-05 |  |
| MTRNR2L3 | 33.8876 | 1.56 | 0.39013 | 3.9939 | 6.50E-05 |  |
| TKTL1 | 11.0641 | 1.54 | 0.39361 | 3.91484 | 9.05E-05 |  |
| MX2 | 1727.99 | 1.53 | 0.39253 | 3.90214 | 9.53E-05 |  |
| LGALS9 | 1894.21 | 1.53 | 0.19642 | 7.78706 | 6.86E-15 |  |
| SAMHD1 | 428.821 | 1.53 | 0.24349 | 6.27761 | 3.44E-10 |  |
| MSMB | 148.405 | 1.53 | 0.37644 | 4.05774 | 4.96E-05 |  |
| IFI35 | 1371.24 | 1.51 | 0.22647 | 6.67183 | 2.53E-11 |  |
| ZMYND15 | 208.244 | 1.50 | 0.25998 | 5.78109 | 7.42E-09 |  |
| MPZ | 89.0583 | 1.50 | 0.31883 | 4.71291 | 2.44E-06 |  |
| IFNL1 | 10.0005 | 1.48 | 0.38153 | 3.87273 | 0.00011 |  |
| BRSK2 | 99.7874 | 1.48 | 0.24644 | 5.99217 | 2.07E-09 |  |
| APCDD1 | 88.4617 | 1.46 | 0.36778 | 3.97296 | 7.10E-05 |  |
| SBSPON | 209.5 | 1.45 | 0.31255 | 4.63357 | 3.59E-06 |  |
| DEFB1 | 775.954 | 1.44 | 0.21763 | 6.60796 | 3.90E-11 |  |
| KL | 21.0677 | 1.43 | 0.39293 | 3.64673 | 0.00027 |  |
| NLRC5 | 838.758 | 1.43 | 0.26143 | 5.45097 | 5.01E-08 |  |
| KIT | 153.087 | 1.41 | 0.29922 | 4.71678 | 2.40E-06 |  |
| CPS1 | 15979.7 | 1.41 | 0.21887 | 6.4224 | 1.34E-10 |  |
| CETP | 13.7699 | 1.40 | 0.39383 | 3.5508 | 0.00038 |  |
| XAF1 | 1823.61 | 1.40 | 0.39034 | 3.57769 | 0.00035 |  |
| SLC16A11 | 52.2819 | 1.39 | 0.3182 | 4.37607 | 1.21E-05 |  |
| OAS1 | 3269.5 | 1.38 | 0.20147 | 6.8359 | 8.15E-12 |  |
| CD22 | 187.967 | 1.37 | 0.23899 | 5.74735 | 9.07E-09 |  |
| HMCN2 | 69.9313 | 1.37 | 0.30425 | 4.50341 | 6.69E-06 |  |
| TM4SF20 | 5038.39 | 1.36 | 0.28603 | 4.75761 | 1.96E-06 |  |
| MX1 | 5381.77 | 1.36 | 0.37983 | 3.58187 | 0.00034 |  |
| C11orf53 | 189.411 | 1.36 | 0.34384 | 3.95627 | 7.61E-05 |  |
| TRIM22 | 71.2347 | 1.36 | 0.37498 | 3.62743 | 0.00029 |  |
| STAT1 | 6179.03 | 1.35 | 0.14436 | 9.38279 | 6.43E-21 |  |
| CELF4 | 88.6766 | 1.34 | 0.31451 | 4.27448 | 1.92E-05 |  |
| LINC01146 | 46.3766 | 1.34 | 0.34042 | 3.93649 | 8.27E-05 |  |
| KAZALD1 | 323.531 | 1.31 | 0.19386 | 6.78279 | 1.18E-11 |  |
| RARRES3 | 285.861 | 1.31 | 0.24702 | 5.31578 | 1.06E-07 |  |
| LINC00570 | 70.4492 | 1.31 | 0.37208 | 3.52593 | 0.00042 |  |
| C8G | 598.628 | 1.30 | 0.25177 | 5.17938 | 2.23E-07 |  |
| PLSCR1 | 1655.85 | 1.30 | 0.21055 | 6.19005 | 6.01E-10 |  |
| IFI27 | 13348.4 | 1.29 | 0.25223 | 5.1269 | 2.95E-07 |  |
| MESP2 | 31.7043 | 1.29 | 0.33784 | 3.82767 | 0.00013 |  |
| PSMB8 | 2987.16 | 1.28 | 0.12332 | 10.4054 | 2.34E-25 |  |
| DHX58 | 883.709 | 1.27 | 0.20982 | 6.07232 | 1.26E-09 |  |
| SLC7A2 | 719.793 | 1.27 | 0.30185 | 4.21761 | 2.47E-05 |  |
| USP18 | 869.874 | 1.27 | 0.23803 | 5.32171 | 1.03E-07 |  |
| CNTD2 | 29.2165 | 1.26 | 0.39022 | 3.21991 | 0.00128 |  |
| BTN3A1 | 589.721 | 1.26 | 0.18098 | 6.94042 | 3.91E-12 |  |
| BTN3A2 | 1368.05 | 1.25 | 0.15887 | 7.85424 | 4.02E-15 |  |
| AQP3 | 4168.78 | 1.25 | 0.1559 | 7.99454 | 1.30E-15 |  |
| B2M | 23870.6 | 1.24 | 0.20694 | 6.01227 | 1.83E-09 |  |
| ARRB2 | 2806.67 | 1.24 | 0.22672 | 5.48517 | 4.13E-08 |  |
| RPRM | 13.3515 | 1.24 | 0.3896 | 3.1916 | 0.00141 |  |
| LOC100419583 | 754.04 | 1.24 | 0.22703 | 5.47343 | 4.41E-08 |  |
| NEIL3 | 457.846 | 1.24 | 0.29169 | 4.25825 | 2.06E-05 |  |
| CDX2 | 1668.12 | 1.23 | 0.20242 | 6.06178 | 1.35E-09 |  |
| LY6E | 10213 | 1.23 | 0.29957 | 4.09008 | 4.31E-05 |  |
| LOC101928994 | 748.577 | 1.22 | 0.32753 | 3.73724 | 0.00019 |  |
| C19orf66 | 452.125 | 1.21 | 0.2384 | 5.06205 | 4.15E-07 |  |
| GDF15 | 1368.48 | 1.21 | 0.18481 | 6.52898 | 6.62E-11 |  |
| STOX1 | 69.044 | 1.20 | 0.27864 | 4.31162 | 1.62E-05 |  |
| ODF3B | 116.88 | 1.20 | 0.34501 | 3.47538 | 0.00051 |  |
| NAT8 | 44.4331 | 1.20 | 0.36921 | 3.23946 | 0.0012 |  |
| IFI6 | 8490.71 | 1.19 | 0.39383 | 3.03363 | 0.00242 |  |
| TAP1 | 2352.56 | 1.19 | 0.26522 | 4.50411 | 6.67E-06 |  |
| PARP9 | 3595.54 | 1.19 | 0.22708 | 5.25801 | 1.46E-07 |  |
| IFIT5 | 1043.48 | 1.19 | 0.26884 | 4.4361 | 9.16E-06 |  |
| IGFALS | 658.681 | 1.19 | 0.23764 | 5.00764 | 5.51E-07 |  |
| APOL6 | 536.736 | 1.19 | 0.19252 | 6.16556 | 7.02E-10 |  |
| PSCA | 2112.78 | 1.19 | 0.26831 | 4.42125 | 9.81E-06 |  |
| ITPKA | 363.681 | 1.18 | 0.24229 | 4.86338 | 1.15E-06 |  |
| KITLG | 107.466 | 1.18 | 0.25989 | 4.52604 | 6.01E-06 |  |
| RUFY4 | 17.6127 | 1.17 | 0.39282 | 2.9836 | 0.00285 |  |
| CCL15 | 29.2884 | 1.17 | 0.38087 | 3.0711 | 0.00213 |  |
| FAM83F | 10.5133 | 1.16 | 0.38888 | 2.9906 | 0.00278 |  |
| BISPR | 256.18 | 1.16 | 0.21179 | 5.48526 | 4.13E-08 |  |
| DOC2A | 165.758 | 1.16 | 0.34164 | 3.39999 | 0.00067 |  |
| IFIT1 | 1518.7 | 1.15 | 0.39298 | 2.93807 | 0.0033 |  |
| ST6GALNAC4 | 938.498 | 1.15 | 0.17926 | 6.42978 | 1.28E-10 |  |
| CYP2B6 | 362.858 | 1.15 | 0.32535 | 3.54115 | 0.0004 |  |
| TGM3 | 22.9515 | 1.15 | 0.38816 | 2.95018 | 0.00318 |  |
| RFTN1 | 104.659 | 1.14 | 0.36483 | 3.1312 | 0.00174 |  |
| LDLRAD1 | 686.228 | 1.14 | 0.17962 | 6.35744 | 2.05E-10 |  |
| CFB | 2024.08 | 1.14 | 0.30138 | 3.7888 | 0.00015 |  |
| LOC100506125 | 14.4817 | 1.14 | 0.39333 | 2.88663 | 0.00389 |  |
| ST6GAL1 | 2491.4 | 1.13 | 0.16853 | 6.72975 | 1.70E-11 |  |
| OAS3 | 7691.76 | 1.13 | 0.2637 | 4.29593 | 1.74E-05 |  |
| HSH2D | 960.19 | 1.13 | 0.19036 | 5.94977 | 2.69E-09 |  |
| CDC42P3 | 11.155 | 1.13 | 0.394 | 2.87234 | 0.00407 |  |
| ATP8A1 | 530.32 | 1.13 | 0.24974 | 4.52279 | 6.10E-06 |  |
| PRR15L | 2434.82 | 1.12 | 0.21164 | 5.30691 | 1.11E-07 |  |
| IL1RN | 38.089 | 1.12 | 0.37033 | 3.02711 | 0.00247 |  |
| FXYD6 | 142.472 | 1.11 | 0.32483 | 3.42915 | 0.00061 |  |
| ASPHD1 | 432.334 | 1.11 | 0.20208 | 5.50145 | 3.77E-08 |  |
| VSIG1 | 2403.19 | 1.11 | 0.25008 | 4.44371 | 8.84E-06 |  |
| ISG15 | 2003.9 | 1.11 | 0.38496 | 2.88445 | 0.00392 |  |
| ACSS1 | 1080.03 | 1.10 | 0.2066 | 5.34219 | 9.18E-08 |  |
| EHF | 2442.11 | 1.10 | 0.26192 | 4.2108 | 2.54E-05 |  |
| SLC43A1 | 187.848 | 1.10 | 0.28323 | 3.89269 | 9.91E-05 |  |
| SLCO4A1 | 475.441 | 1.10 | 0.25752 | 4.26612 | 1.99E-05 |  |
| CKMT1A | 251.697 | 1.10 | 0.21823 | 5.0298 | 4.91E-07 |  |
| CORO2A | 2872.45 | 1.09 | 0.1629 | 6.70264 | 2.05E-11 |  |
| HLA-C | 14918.6 | 1.09 | 0.18311 | 5.96009 | 2.52E-09 |  |
| SEMA6A | 81.9129 | 1.09 | 0.30433 | 3.58352 | 0.00034 |  |
| ASRGL1 | 238.648 | 1.09 | 0.25868 | 4.20127 | 2.65E-05 |  |
| CCDC42B | 28.0211 | 1.09 | 0.37555 | 2.88997 | 0.00385 |  |
| SLC51B | 1350.41 | 1.08 | 0.31808 | 3.41048 | 0.00065 |  |
| SYP | 79.629 | 1.08 | 0.29561 | 3.6661 | 0.00025 |  |
| DEGS2 | 547.738 | 1.08 | 0.20122 | 5.38538 | 7.23E-08 |  |
| CD38 | 19.6465 | 1.08 | 0.39304 | 2.75347 | 0.0059 |  |
| PLA2G4C | 202.845 | 1.08 | 0.26403 | 4.09756 | 4.18E-05 |  |
| RENBP | 178.932 | 1.08 | 0.28092 | 3.84881 | 0.00012 |  |
| CCDC34 | 524.19 | 1.08 | 0.21297 | 5.05878 | 4.22E-07 |  |
| TMPRSS3 | 106.555 | 1.08 | 0.29021 | 3.70843 | 0.00021 |  |
| SPRY1 | 332.166 | 1.07 | 0.21384 | 5.00207 | 5.67E-07 |  |
| SP5 | 123.552 | 1.07 | 0.33659 | 3.17574 | 0.00149 |  |
| MECOM | 489.026 | 1.06 | 0.27101 | 3.91125 | 9.18E-05 |  |
| KCNV1 | 91.794 | 1.06 | 0.28285 | 3.74302 | 0.00018 |  |
| CEBPA | 452.63 | 1.06 | 0.2487 | 4.25648 | 2.08E-05 |  |
| KIAA1211L | 280.899 | 1.06 | 0.27028 | 3.91519 | 9.03E-05 |  |
| PDK4 | 200.593 | 1.06 | 0.26785 | 3.94448 | 8.00E-05 |  |
| TMEM82 | 166.787 | 1.06 | 0.28323 | 3.72545 | 0.00019 |  |
| CA11 | 354.426 | 1.05 | 0.28149 | 3.7392 | 0.00018 |  |
| CATIP | 22.3093 | 1.05 | 0.38054 | 2.76262 | 0.00573 |  |
| SYBU | 408.726 | 1.05 | 0.32678 | 3.20763 | 0.00134 |  |
| NTHL1 | 698.962 | 1.05 | 0.23726 | 4.40645 | 1.05E-05 |  |
| EPB41L4B | 1073.22 | 1.04 | 0.25133 | 4.15018 | 3.32E-05 |  |
| MAP1A | 401.88 | 1.04 | 0.25989 | 4.01068 | 6.05E-05 |  |
| HLA-A | 22986.2 | 1.04 | 0.16543 | 6.28854 | 3.20E-10 |  |
| LOC100133985 | 16.228 | 1.04 | 0.39005 | 2.66447 | 0.00771 |  |
| FAM198B | 63.0685 | 1.04 | 0.30964 | 3.3551 | 0.00079 |  |
| FKBP5 | 1031.44 | 1.04 | 0.27315 | 3.7999 | 0.00014 |  |
| PALD1 | 108.404 | 1.04 | 0.28465 | 3.64445 | 0.00027 |  |
| ITGAM | 127.441 | 1.04 | 0.33048 | 3.13406 | 0.00172 |  |
| ASS1 | 5865.46 | 1.03 | 0.18937 | 5.46359 | 4.67E-08 |  |
| OTUB2 | 81.7673 | 1.03 | 0.28281 | 3.64965 | 0.00026 |  |
| LOC285740 | 36.843 | 1.03 | 0.36639 | 2.81549 | 0.00487 |  |
| ANPEP | 5045.61 | 1.03 | 0.14392 | 7.16566 | 7.74E-13 |  |
| PSME2 | 2328.07 | 1.03 | 0.11829 | 8.71362 | 2.94E-18 |  |
| LINC00565 | 18.0711 | 1.03 | 0.39397 | 2.60852 | 0.00909 |  |
| LRRC73 | 55.2322 | 1.03 | 0.30431 | 3.37185 | 0.00075 |  |
| C17orf82 | 10.8999 | 1.03 | 0.39398 | 2.60201 | 0.00927 |  |
| ARMC12 | 17.8867 | 1.02 | 0.39148 | 2.61334 | 0.00897 |  |
| DDX58 | 1966.58 | 1.02 | 0.28322 | 3.6088 | 0.00031 |  |
| CREB3L1 | 6605.35 | 1.02 | 0.28905 | 3.52781 | 0.00042 |  |
| SMOC1 | 1599.49 | 1.02 | 0.24984 | 4.08105 | 4.48E-05 |  |
| PPP1R14D | 270.181 | 1.01 | 0.27977 | 3.62071 | 0.00029 |  |
| RTN4RL2 | 277.328 | 1.01 | 0.27489 | 3.66555 | 0.00025 |  |
| PGR | 24.2497 | 1.00 | 0.39138 | 2.56144 | 0.01042 |  |
| LAP3 | 2329.26 | 1.00 | 0.15182 | 6.59643 | 4.21E-11 |  |
| ELN | 156.553 | 1.00 | 0.36833 | 2.71656 | 0.0066 |  |
| SPATA18 | 66.4004 | 1.00 | 0.3804 | 2.62947 | 0.00855 |  |
| KIAA1147 | 3702.8 | 1.00 | 0.20137 | 4.96651 | 6.82E-07 |  |
| MSX2 | 300.62 | 1.00 | 0.20364 | 4.90454 | 9.36E-07 |  |
| GPR37 | 202.446 | 1.00 | 0.21332 | 4.68172 | 2.84E-06 |  |
| OAS2 | 1960.9 | 1.00 | 0.37459 | 2.66315 | 0.00774 |  |
| CRB2 | 31.4101 | 1.00 | 0.34813 | 2.86232 | 0.00421 |  |
| ADGRG5 | 433.25 | 1.00 | 0.22585 | 4.41156 | 1.03E-05 |  |
| FAM46A | 377.184 | 0.99 | 0.24957 | 3.98174 | 6.84E-05 |  |
| LINC01485 | 30.8023 | 0.99 | 0.36911 | 2.68316 | 0.00729 |  |
| TYMSOS | 23.5377 | 0.99 | 0.36675 | 2.69801 | 0.00698 |  |
| SLC5A11 | 1417.75 | 0.99 | 0.21364 | 4.61375 | 3.95E-06 |  |
| RASSF9 | 123.482 | 0.98 | 0.26724 | 3.67557 | 0.00024 |  |
| GRHL3 | 127.018 | 0.98 | 0.32562 | 3.01186 | 0.0026 |  |
| GSDMB | 188.972 | 0.98 | 0.3244 | 3.01988 | 0.00253 |  |
| CEL | 61.244 | 0.98 | 0.32863 | 2.96867 | 0.00299 |  |
| MNX1-AS1 | 108.867 | 0.97 | 0.24704 | 3.94464 | 7.99E-05 |  |
| HSPA4L | 120.203 | 0.97 | 0.23243 | 4.17662 | 2.96E-05 |  |
| CYP2J2 | 135.789 | 0.97 | 0.27373 | 3.54504 | 0.00039 |  |
| MNS1 | 200.289 | 0.97 | 0.26474 | 3.65168 | 0.00026 |  |
| RASGEF1B | 84.4123 | 0.97 | 0.29485 | 3.27311 | 0.00106 |  |
| TMEM151A | 878.669 | 0.96 | 0.31659 | 3.04495 | 0.00233 |  |
| C4orf33 | 493.365 | 0.96 | 0.20776 | 4.63646 | 3.54E-06 |  |
| CCDC78 | 149.412 | 0.96 | 0.22867 | 4.2102 | 2.55E-05 |  |
| SLC27A2 | 341.376 | 0.96 | 0.19076 | 5.03353 | 4.82E-07 |  |
| NHSL1 | 611.746 | 0.96 | 0.19702 | 4.87278 | 1.10E-06 |  |
| GULP1 | 853.58 | 0.96 | 0.19756 | 4.85517 | 1.20E-06 |  |
| NMI | 877.428 | 0.96 | 0.20819 | 4.6001 | 4.22E-06 |  |
| EOMES | 18.5895 | 0.96 | 0.39337 | 2.43421 | 0.01492 |  |
| CCDC144NL-AS1 | 9.39307 | 0.96 | 0.39276 | 2.43743 | 0.01479 |  |
| TMED6 | 115.824 | 0.96 | 0.3006 | 3.18442 | 0.00145 |  |
| SMOC2 | 2523.73 | 0.95 | 0.29919 | 3.18858 | 0.00143 |  |
| TRPV2 | 10.7579 | 0.95 | 0.39371 | 2.42253 | 0.01541 |  |
| RAPGEF3 | 216.82 | 0.95 | 0.19861 | 4.79609 | 1.62E-06 |  |
| SGK2 | 866.979 | 0.95 | 0.19102 | 4.97521 | 6.52E-07 |  |
| EGFR-AS1 | 398.034 | 0.95 | 0.19318 | 4.89486 | 9.84E-07 |  |
| GAS6-AS1 | 55.4633 | 0.94 | 0.30539 | 3.08793 | 0.00202 |  |
| PAPSS2 | 690.696 | 0.94 | 0.29855 | 3.1541 | 0.00161 |  |
| LRRC66 | 507.431 | 0.94 | 0.21893 | 4.29732 | 1.73E-05 |  |
| TESC | 1298.95 | 0.94 | 0.26901 | 3.49563 | 0.00047 |  |
| VSTM2L | 305.878 | 0.94 | 0.23724 | 3.95837 | 7.55E-05 |  |
| ADGRD1 | 411.484 | 0.94 | 0.22802 | 4.11762 | 3.83E-05 |  |
| HOXD4 | 17.2168 | 0.94 | 0.3832 | 2.44999 | 0.01429 |  |
| PHACTR1 | 30.1925 | 0.94 | 0.35245 | 2.66203 | 0.00777 |  |
| MTRNR2L10 | 2097.67 | 0.94 | 0.22565 | 4.15608 | 3.24E-05 |  |
| CA8 | 32.0127 | 0.94 | 0.37865 | 2.47649 | 0.01327 |  |
| MUC2 | 123.628 | 0.94 | 0.37394 | 2.50709 | 0.01217 |  |
| TRIM14 | 4758.35 | 0.94 | 0.14346 | 6.52437 | 6.83E-11 |  |
| RBM11 | 64.1022 | 0.94 | 0.27272 | 3.43022 | 0.0006 |  |
| IFIH1 | 1622.49 | 0.93 | 0.27582 | 3.38992 | 0.0007 |  |
| RORC | 594.956 | 0.93 | 0.19401 | 4.81103 | 1.50E-06 |  |
| THEMIS2 | 120.077 | 0.93 | 0.25023 | 3.72291 | 0.0002 |  |
| SGPP1 | 1236.88 | 0.93 | 0.17762 | 5.2416 | 1.59E-07 |  |
| PNPLA1 | 146.74 | 0.93 | 0.22024 | 4.22628 | 2.38E-05 |  |
| EVA1C | 1390.38 | 0.93 | 0.25201 | 3.68784 | 0.00023 |  |
| LINC01410 | 33.6483 | 0.93 | 0.32845 | 2.82655 | 0.00471 |  |
| P2RX5 | 31.4852 | 0.93 | 0.34432 | 2.69287 | 0.00708 |  |
| B3GNT7 | 2024.67 | 0.93 | 0.224 | 4.13767 | 3.51E-05 |  |
| RDH10 | 938.604 | 0.93 | 0.31551 | 2.93273 | 0.00336 |  |
| NRGN | 25.8992 | 0.92 | 0.36053 | 2.56129 | 0.01043 |  |
| HMGN5 | 408.897 | 0.92 | 0.30362 | 3.04098 | 0.00236 |  |
| BIRC3 | 1244.13 | 0.92 | 0.20021 | 4.58754 | 4.48E-06 |  |
| WNT4 | 136.353 | 0.92 | 0.22524 | 4.07116 | 4.68E-05 |  |
| COLCA2 | 524.77 | 0.92 | 0.33548 | 2.73208 | 0.00629 |  |
| LOC643201 | 129.183 | 0.92 | 0.33107 | 2.76502 | 0.00569 |  |
| PIP5K1B | 275.086 | 0.91 | 0.32081 | 2.85076 | 0.00436 |  |
| GATA2-AS1 | 174.012 | 0.91 | 0.33378 | 2.73577 | 0.00622 |  |
| GRAMD1C | 125.011 | 0.91 | 0.2692 | 3.38986 | 0.0007 |  |
| GPR27 | 22.6461 | 0.91 | 0.36581 | 2.49064 | 0.01275 |  |
| RSPH1 | 55.1832 | 0.91 | 0.30801 | 2.9473 | 0.00321 |  |
| TLR3 | 183.489 | 0.91 | 0.25464 | 3.56321 | 0.00037 |  |
| TGFBR3 | 163.796 | 0.91 | 0.25033 | 3.6242 | 0.00029 |  |
| SLCO3A1 | 990.193 | 0.91 | 0.25455 | 3.55823 | 0.00037 |  |
| VAV3 | 243.295 | 0.91 | 0.31546 | 2.87052 | 0.0041 |  |
| HOXC5 | 35.7246 | 0.91 | 0.35773 | 2.5303 | 0.0114 |  |
| MUC5B | 34097.2 | 0.90 | 0.22136 | 4.07205 | 4.66E-05 |  |
| SLC45A4 | 5170.82 | 0.90 | 0.11806 | 7.62543 | 2.43E-14 |  |
| LRIG1 | 361.576 | 0.90 | 0.22026 | 4.08322 | 4.44E-05 |  |
| ERN2 | 1681.4 | 0.90 | 0.26139 | 3.43937 | 0.00058 |  |
| SLC15A3 | 21.9953 | 0.90 | 0.37468 | 2.39758 | 0.0165 |  |
| CXCL1 | 510.566 | 0.90 | 0.31832 | 2.82038 | 0.0048 |  |
| ATP6V0E2-AS1 | 26.1765 | 0.90 | 0.36012 | 2.48923 | 0.0128 |  |
| KCNK3 | 95.5755 | 0.90 | 0.32654 | 2.74246 | 0.0061 |  |
| WNK4 | 192.872 | 0.90 | 0.21378 | 4.18669 | 2.83E-05 |  |
| ST3GAL4-AS1 | 108.239 | 0.89 | 0.27048 | 3.30426 | 0.00095 |  |
| PRR22 | 69.8854 | 0.89 | 0.303 | 2.94733 | 0.00321 |  |
| ADAMTS14 | 78.3564 | 0.89 | 0.3022 | 2.95424 | 0.00313 |  |
| SCN8A | 1494.57 | 0.89 | 0.19942 | 4.46969 | 7.83E-06 |  |
| APIP | 295.808 | 0.89 | 0.21174 | 4.20862 | 2.57E-05 |  |
| MYB | 87.1103 | 0.89 | 0.32031 | 2.77955 | 0.00544 |  |
| HOXA10 | 155.39 | 0.89 | 0.23665 | 3.754 | 0.00017 |  |
| LINC01124 | 55.7618 | 0.89 | 0.30186 | 2.94285 | 0.00325 |  |
| PLEKHB1 | 530.141 | 0.89 | 0.18184 | 4.87859 | 1.07E-06 |  |
| CNNM1 | 660.779 | 0.89 | 0.25885 | 3.42643 | 0.00061 |  |
| CTSS | 361.639 | 0.89 | 0.31278 | 2.83279 | 0.00461 |  |
| SAMD9L | 851.909 | 0.89 | 0.27335 | 3.24071 | 0.00119 |  |
| ACTL8 | 64.838 | 0.89 | 0.32827 | 2.69598 | 0.00702 |  |
| IFI30 | 238.555 | 0.88 | 0.21285 | 4.13478 | 3.55E-05 |  |
| TMPRSS2 | 356.876 | 0.88 | 0.30793 | 2.858 | 0.00426 |  |
| BRCA2 | 738.304 | 0.88 | 0.14613 | 6.01978 | 1.75E-09 |  |
| TNFSF10 | 293.478 | 0.88 | 0.33861 | 2.58615 | 0.00971 |  |
| TAP2 | 1846.25 | 0.87 | 0.17495 | 4.99856 | 5.78E-07 |  |
| CCDC64 | 519.011 | 0.87 | 0.16268 | 5.37554 | 7.64E-08 |  |
| CDR1 | 161.042 | 0.87 | 0.32225 | 2.71004 | 0.00673 |  |
| PRKCQ-AS1 | 393.463 | 0.87 | 0.16256 | 5.3611 | 8.27E-08 |  |
| FAM81A | 23.0568 | 0.87 | 0.36353 | 2.3928 | 0.01672 |  |
| ADH6 | 656.458 | 0.87 | 0.25795 | 3.36375 | 0.00077 |  |
| RASL11A | 110.886 | 0.87 | 0.27601 | 3.14236 | 0.00168 |  |
| CEACAM5 | 5742.32 | 0.87 | 0.21449 | 4.0395 | 5.36E-05 |  |
| GNAI1 | 577.629 | 0.86 | 0.14772 | 5.85271 | 4.84E-09 |  |
| CYSTM1 | 3259.79 | 0.86 | 0.22524 | 3.83712 | 0.00012 |  |
| LOC115110 | 245.946 | 0.86 | 0.21265 | 4.05954 | 4.92E-05 |  |
| GAL3ST2 | 436.66 | 0.86 | 0.23266 | 3.70377 | 0.00021 |  |
| SLC1A7 | 169.815 | 0.86 | 0.30428 | 2.83095 | 0.00464 |  |
| SORBS1 | 241.044 | 0.86 | 0.21818 | 3.93865 | 8.19E-05 |  |
| CGREF1 | 524.305 | 0.86 | 0.18423 | 4.66269 | 3.12E-06 |  |
| HOXD13 | 69.5165 | 0.86 | 0.31879 | 2.69331 | 0.00707 |  |
| DOK7 | 433.365 | 0.86 | 0.21322 | 4.01991 | 5.82E-05 |  |
| GPR19 | 29.2528 | 0.86 | 0.35566 | 2.40929 | 0.01598 |  |
| MUC5AC | 70701.6 | 0.86 | 0.25246 | 3.39175 | 0.00069 |  |
| SLC12A2 | 1631.88 | 0.85 | 0.2114 | 4.04391 | 5.26E-05 |  |
| PEX5L | 107.795 | 0.85 | 0.30571 | 2.79512 | 0.00519 |  |
| IRX3 | 217.992 | 0.85 | 0.21921 | 3.89249 | 9.92E-05 |  |
| CD27-AS1 | 106.205 | 0.85 | 0.32201 | 2.64929 | 0.00807 |  |
| LINC01342 | 32.5629 | 0.85 | 0.34316 | 2.4818 | 0.01307 |  |
| RELL2 | 34.044 | 0.85 | 0.33819 | 2.51648 | 0.01185 |  |
| CLPSL1 | 57.448 | 0.85 | 0.33043 | 2.57486 | 0.01003 |  |
| EXOC3L4 | 166.559 | 0.85 | 0.28483 | 2.98558 | 0.00283 |  |
| PHACTR3 | 292.181 | 0.85 | 0.34782 | 2.44074 | 0.01466 |  |
| ARL11 | 261.415 | 0.85 | 0.23786 | 3.56457 | 0.00036 |  |
| PDE10A | 721.819 | 0.85 | 0.16986 | 4.9884 | 6.09E-07 |  |
| TFF1 | 10905 | 0.85 | 0.18966 | 4.45936 | 8.22E-06 |  |
| COL16A1 | 147.723 | 0.85 | 0.33416 | 2.52963 | 0.01142 |  |
| GJB1 | 685.312 | 0.84 | 0.26159 | 3.22789 | 0.00125 |  |
| ESCO2 | 301.343 | 0.84 | 0.21871 | 3.8498 | 0.00012 |  |
| XXYLT1-AS2 | 250.885 | 0.84 | 0.32994 | 2.54299 | 0.01099 |  |
| CCDC24 | 200.98 | 0.84 | 0.24754 | 3.38773 | 0.0007 |  |
| GLYATL1 | 537.153 | 0.84 | 0.23077 | 3.63319 | 0.00028 |  |
| MESP1 | 54.6113 | 0.84 | 0.33075 | 2.53425 | 0.01127 |  |
| NAP1L2 | 138.808 | 0.84 | 0.26568 | 3.15473 | 0.00161 |  |
| RPARP-AS1 | 262.639 | 0.84 | 0.20405 | 4.10569 | 4.03E-05 |  |
| CYBRD1 | 483.393 | 0.84 | 0.30174 | 2.772 | 0.00557 |  |
| HOXC6 | 170.997 | 0.83 | 0.2353 | 3.5479 | 0.00039 |  |
| SNORA24 | 30.6738 | 0.83 | 0.33441 | 2.48995 | 0.01278 |  |
| LRRCC1 | 405.685 | 0.83 | 0.18563 | 4.47464 | 7.65E-06 |  |
| HLA-H | 525.298 | 0.83 | 0.21268 | 3.90495 | 9.42E-05 |  |
| PARP10 | 2269.25 | 0.83 | 0.23681 | 3.50386 | 0.00046 |  |
| LYSMD2 | 158.44 | 0.83 | 0.20727 | 3.99966 | 6.34E-05 |  |
| EIF2AK2 | 2393.92 | 0.83 | 0.21347 | 3.88169 | 0.0001 |  |
| CD47 | 632.961 | 0.83 | 0.20294 | 4.07793 | 4.54E-05 |  |
| AIM1 | 383.548 | 0.83 | 0.33449 | 2.47313 | 0.01339 |  |
| ANK3 | 756.136 | 0.83 | 0.19936 | 4.14585 | 3.39E-05 |  |
| COL9A2 | 41.2331 | 0.83 | 0.33008 | 2.50053 | 0.0124 |  |
| TMEM198 | 149.184 | 0.82 | 0.30154 | 2.73468 | 0.00624 |  |
| PNPT1 | 1533.06 | 0.82 | 0.16091 | 5.1247 | 2.98E-07 |  |
| ARHGEF38 | 526.541 | 0.82 | 0.25798 | 3.19275 | 0.00141 |  |
| TM4SF4 | 18900.4 | 0.82 | 0.16794 | 4.90303 | 9.44E-07 |  |
| COMTD1 | 1477.55 | 0.82 | 0.14972 | 5.49522 | 3.90E-08 |  |
| KIF20B | 1293.18 | 0.82 | 0.17785 | 4.62488 | 3.75E-06 |  |
| LOC101929412 | 326.077 | 0.82 | 0.3256 | 2.52487 | 0.01157 |  |
| RNF43 | 1522.03 | 0.82 | 0.15186 | 5.41142 | 6.25E-08 |  |
| TST | 1091.52 | 0.82 | 0.14327 | 5.7188 | 1.07E-08 |  |
| HPDL | 156.025 | 0.82 | 0.24144 | 3.38299 | 0.00072 |  |
| ST8SIA4 | 355.382 | 0.82 | 0.19596 | 4.16388 | 3.13E-05 |  |
| LBX2-AS1 | 705.017 | 0.82 | 0.16812 | 4.85057 | 1.23E-06 |  |
| ETFDH | 693.842 | 0.81 | 0.14631 | 5.56714 | 2.59E-08 |  |
| NSMCE4A | 672.646 | 0.81 | 0.1301 | 6.24935 | 4.12E-10 |  |
| ENPP1 | 101.917 | 0.81 | 0.29593 | 2.7322 | 0.00629 |  |
| PPM1H | 541.813 | 0.81 | 0.16179 | 4.99624 | 5.85E-07 |  |
| NKX2-8 | 58.779 | 0.81 | 0.29786 | 2.70695 | 0.00679 |  |
| TNFSF13 | 544.242 | 0.81 | 0.18232 | 4.42041 | 9.85E-06 |  |
| COQ2 | 570.583 | 0.81 | 0.1451 | 5.55339 | 2.80E-08 |  |
| CHCHD10 | 1160.47 | 0.81 | 0.14248 | 5.6545 | 1.56E-08 |  |
| MND1 | 162.895 | 0.80 | 0.24718 | 3.2492 | 0.00116 |  |
| COA6 | 392.68 | 0.80 | 0.16154 | 4.96792 | 6.77E-07 |  |
| BCCIP | 1841.59 | 0.80 | 0.19595 | 4.07777 | 4.55E-05 |  |
| BCO1 | 220.387 | 0.80 | 0.24196 | 3.29716 | 0.00098 |  |
| SULT1A2 | 615.608 | 0.80 | 0.18277 | 4.3604 | 1.30E-05 |  |
| PARP14 | 5524.99 | 0.80 | 0.14519 | 5.48824 | 4.06E-08 |  |
| TMEM253 | 480.304 | 0.80 | 0.21149 | 3.76596 | 0.00017 |  |
| PYROXD2 | 295.543 | 0.80 | 0.21417 | 3.71503 | 0.0002 |  |
| SDSL | 487.094 | 0.79 | 0.18528 | 4.28429 | 1.83E-05 |  |
| FBXO43 | 88.1398 | 0.79 | 0.2952 | 2.68894 | 0.00717 |  |
| TSPAN8 | 8402.97 | 0.79 | 0.25316 | 3.13226 | 0.00173 |  |
| IFT27 | 316.97 | 0.79 | 0.18306 | 4.32559 | 1.52E-05 |  |
| LOC101927630 | 75.5984 | 0.79 | 0.31303 | 2.52635 | 0.01153 |  |
| ARL14 | 552.035 | 0.79 | 0.2348 | 3.36412 | 0.00077 |  |
| RAPGEFL1 | 3043.85 | 0.79 | 0.144 | 5.47596 | 4.35E-08 |  |
| NAP1L5 | 66.407 | 0.79 | 0.30757 | 2.5553 | 0.01061 |  |
| ISOC2 | 1710.89 | 0.78 | 0.18687 | 4.19636 | 2.71E-05 |  |
| NPM3 | 696.054 | 0.78 | 0.17273 | 4.53439 | 5.78E-06 |  |
| MPC2 | 2129.83 | 0.78 | 0.11768 | 6.65253 | 2.88E-11 |  |
| RFX5 | 1198.03 | 0.78 | 0.13341 | 5.86821 | 4.41E-09 |  |
| OAT | 3568.8 | 0.78 | 0.16583 | 4.7205 | 2.35E-06 |  |
| CADPS2 | 341.896 | 0.78 | 0.15534 | 5.03593 | 4.76E-07 |  |
| CDK1 | 1087.08 | 0.78 | 0.16694 | 4.66856 | 3.03E-06 |  |
| ZWINT | 1538.02 | 0.78 | 0.16 | 4.86538 | 1.14E-06 |  |
| HMGB2 | 2792.51 | 0.78 | 0.1724 | 4.50192 | 6.73E-06 |  |
| PIR | 398.456 | 0.78 | 0.15638 | 4.95907 | 7.08E-07 |  |
| SFTA2 | 394.649 | 0.77 | 0.26159 | 2.96225 | 0.00305 |  |
| TRANK1 | 417.159 | 0.77 | 0.21932 | 3.52999 | 0.00042 |  |
| CA2 | 514.736 | 0.77 | 0.16404 | 4.71319 | 2.44E-06 |  |
| HOXB5 | 296.584 | 0.77 | 0.2161 | 3.57749 | 0.00035 |  |
| WBSCR27 | 317.258 | 0.77 | 0.177 | 4.36546 | 1.27E-05 |  |
| KLK10 | 3789.7 | 0.77 | 0.27062 | 2.85483 | 0.00431 |  |
| MFSD6L | 140.752 | 0.77 | 0.32336 | 2.38672 | 0.017 |  |
| BAX | 1239.35 | 0.77 | 0.12985 | 5.94243 | 2.81E-09 |  |
| DDX60L | 1520.43 | 0.77 | 0.28509 | 2.6853 | 0.00725 |  |
| PSAPL1 | 110.38 | 0.77 | 0.28847 | 2.65323 | 0.00797 |  |
| LOC728743 | 202.484 | 0.76 | 0.22228 | 3.44151 | 0.00058 |  |
| F7 | 277.694 | 0.76 | 0.2979 | 2.56625 | 0.01028 |  |
| PHF11 | 856.06 | 0.76 | 0.17862 | 4.27195 | 1.94E-05 |  |
| MUC13 | 23098.8 | 0.76 | 0.22394 | 3.40458 | 0.00066 |  |
| C21orf58 | 369.343 | 0.76 | 0.20589 | 3.70214 | 0.00021 |  |
| DCLRE1C | 1056.23 | 0.76 | 0.17458 | 4.36447 | 1.27E-05 |  |
| FOXJ1 | 407.925 | 0.76 | 0.26316 | 2.89115 | 0.00384 |  |
| DGAT2 | 53.0075 | 0.76 | 0.31347 | 2.42604 | 0.01526 |  |
| SKA3 | 725.745 | 0.76 | 0.15483 | 4.90567 | 9.31E-07 |  |
| NPDC1 | 1266.57 | 0.76 | 0.13363 | 5.6828 | 1.33E-08 |  |
| RPUSD1 | 1981.08 | 0.76 | 0.14918 | 5.0894 | 3.59E-07 |  |
| SH3PXD2A | 792.355 | 0.76 | 0.21168 | 3.58651 | 0.00034 |  |
| RBBP8NL | 411.254 | 0.76 | 0.16424 | 4.61778 | 3.88E-06 |  |
| PSMB10 | 783.714 | 0.76 | 0.1734 | 4.37008 | 1.24E-05 |  |
| ABCG2 | 265.038 | 0.75 | 0.21522 | 3.50273 | 0.00046 |  |
| RIBC2 | 73.4421 | 0.75 | 0.28163 | 2.67374 | 0.0075 |  |
| CPLX2 | 15905 | 0.75 | 0.26267 | 2.85958 | 0.00424 |  |
| EDAR | 261.289 | 0.75 | 0.30709 | 2.44198 | 0.01461 |  |
| LOC101927027 | 406.682 | 0.75 | 0.18151 | 4.1293 | 3.64E-05 |  |
| IL7 | 65.5943 | 0.75 | 0.28017 | 2.67273 | 0.00752 |  |
| ZNF703 | 1723.39 | 0.75 | 0.19101 | 3.91973 | 8.86E-05 |  |
| PHGR1 | 2180.2 | 0.75 | 0.29606 | 2.51756 | 0.01182 |  |
| ESPN | 528.683 | 0.74 | 0.16163 | 4.59081 | 4.42E-06 |  |
| ATP5G1 | 1129.6 | 0.74 | 0.14779 | 5.01803 | 5.22E-07 |  |
| IL15RA | 264.101 | 0.74 | 0.22289 | 3.32177 | 0.00089 |  |
| PCLO | 682.721 | 0.74 | 0.20227 | 3.6548 | 0.00026 |  |
| KIF24 | 370.755 | 0.74 | 0.21386 | 3.4473 | 0.00057 |  |
| CENPK | 353.319 | 0.74 | 0.20066 | 3.67007 | 0.00024 |  |
| CREG1 | 3091.59 | 0.74 | 0.17779 | 4.13622 | 3.53E-05 |  |
| MAGEF1 | 2685.34 | 0.73 | 0.19969 | 3.67154 | 0.00024 |  |
| NAA38 | 1001.32 | 0.73 | 0.17003 | 4.30944 | 1.64E-05 |  |
| TACC2 | 5425.38 | 0.73 | 0.14032 | 5.22144 | 1.78E-07 |  |
| NMU | 90.649 | 0.73 | 0.2907 | 2.51173 | 0.01201 |  |
| TRIM38 | 1175.84 | 0.73 | 0.18093 | 4.03475 | 5.47E-05 |  |
| SMC4 | 3125.98 | 0.73 | 0.19187 | 3.80428 | 0.00014 |  |
| ADGRG2 | 72.8746 | 0.73 | 0.30171 | 2.41748 | 0.01563 |  |
| DPY19L2 | 83.2129 | 0.73 | 0.27275 | 2.67273 | 0.00752 |  |
| FTCDNL1 | 67.4055 | 0.73 | 0.27547 | 2.64339 | 0.00821 |  |
| HMMR | 562.975 | 0.73 | 0.15368 | 4.73578 | 2.18E-06 |  |
| CAPN9 | 2395.01 | 0.73 | 0.16176 | 4.49708 | 6.89E-06 |  |
| KIAA0101 | 690.843 | 0.73 | 0.13796 | 5.26388 | 1.41E-07 |  |
| DNAJC12 | 64.4691 | 0.72 | 0.28035 | 2.58514 | 0.00973 |  |
| CACNB2 | 181.437 | 0.72 | 0.24883 | 2.91056 | 0.00361 |  |
| ZNF367 | 388.471 | 0.72 | 0.15419 | 4.68829 | 2.76E-06 |  |
| NMB | 765.399 | 0.72 | 0.24673 | 2.9247 | 0.00345 |  |
| SEPHS2 | 2878.08 | 0.72 | 0.12205 | 5.91089 | 3.40E-09 |  |
| OAF | 167.385 | 0.72 | 0.28556 | 2.5257 | 0.01155 |  |
| TAPBPL | 220.763 | 0.72 | 0.17897 | 4.02631 | 5.67E-05 |  |
| TREX1 | 329.759 | 0.72 | 0.19564 | 3.6819 | 0.00023 |  |
| SLC45A3 | 1179.1 | 0.72 | 0.15838 | 4.54654 | 5.45E-06 |  |
| XK | 383.607 | 0.72 | 0.25647 | 2.80738 | 0.00499 |  |
| VSIG10L | 609.01 | 0.72 | 0.17562 | 4.09541 | 4.21E-05 |  |
| GUCY1A3 | 131.168 | 0.72 | 0.24765 | 2.90359 | 0.00369 |  |
| CISD1 | 703.105 | 0.72 | 0.15799 | 4.5425 | 5.56E-06 |  |
| PABPC1L | 1022.48 | 0.72 | 0.1715 | 4.17999 | 2.92E-05 |  |
| GPT2 | 1506.49 | 0.72 | 0.10477 | 6.82818 | 8.60E-12 |  |
| TDRD7 | 816.397 | 0.71 | 0.16622 | 4.29348 | 1.76E-05 |  |
| BDKRB2 | 1042.44 | 0.71 | 0.2703 | 2.64005 | 0.00829 |  |
| DUSP16 | 1451.2 | 0.71 | 0.1235 | 5.77568 | 7.66E-09 |  |
| RAD51C | 252.723 | 0.71 | 0.18889 | 3.77609 | 0.00016 |  |
| DSCC1 | 203.184 | 0.71 | 0.22193 | 3.21292 | 0.00131 |  |
| TYMP | 1490.99 | 0.71 | 0.19199 | 3.71278 | 0.0002 |  |
| CHTF18 | 1256.07 | 0.71 | 0.2427 | 2.93548 | 0.00333 |  |
| BDH1 | 478.903 | 0.71 | 0.23107 | 3.07665 | 0.00209 |  |
| RBM47 | 5711.82 | 0.71 | 0.09461 | 7.51394 | 5.74E-14 |  |
| HAGHL | 366.15 | 0.71 | 0.23028 | 3.06782 | 0.00216 |  |
| SH3BGRL2 | 1072.02 | 0.71 | 0.20452 | 3.4526 | 0.00056 |  |
| IRF9 | 2371.16 | 0.71 | 0.19032 | 3.70945 | 0.00021 |  |
| HSDL2 | 912.112 | 0.70 | 0.16844 | 4.18354 | 2.87E-05 |  |
| ELFN1-AS1 | 326.789 | 0.70 | 0.29161 | 2.41312 | 0.01582 |  |
| STS | 549.758 | 0.70 | 0.29507 | 2.38172 | 0.01723 |  |
| OMA1 | 137.125 | 0.70 | 0.28192 | 2.48625 | 0.01291 |  |
| FOXL2 | 174.498 | 0.70 | 0.24058 | 2.91323 | 0.00358 |  |
| SLPI | 2727.5 | 0.70 | 0.20494 | 3.41616 | 0.00064 |  |
| B4GALT6 | 114.902 | 0.70 | 0.23092 | 3.02972 | 0.00245 |  |
| MTRNR2L1 | 14265.9 | 0.70 | 0.09856 | 7.09491 | 1.29E-12 |  |
| MASTL | 1016.67 | 0.70 | 0.12938 | 5.40227 | 6.58E-08 |  |
| MLKL | 1028.34 | 0.70 | 0.22047 | 3.16662 | 0.00154 |  |
| ISCU | 1510.85 | 0.70 | 0.13339 | 5.21945 | 1.79E-07 |  |
| GSTM4 | 503.293 | 0.70 | 0.23581 | 2.95033 | 0.00317 |  |
| ANKRD18B | 266.014 | 0.69 | 0.21595 | 3.20945 | 0.00133 |  |
| DEPDC1 | 360.231 | 0.69 | 0.15998 | 4.32756 | 1.51E-05 |  |
| AQP7 | 94.9295 | 0.69 | 0.264 | 2.62035 | 0.00878 |  |
| TBXAS1 | 187.622 | 0.69 | 0.26315 | 2.62875 | 0.00857 |  |
| KRT15 | 1432.84 | 0.69 | 0.2015 | 3.43023 | 0.0006 |  |
| WDR89 | 453.686 | 0.69 | 0.1452 | 4.75811 | 1.95E-06 |  |
| LOC100506100 | 58.434 | 0.69 | 0.28887 | 2.39057 | 0.01682 |  |
| TTC39A | 654.811 | 0.69 | 0.27129 | 2.54289 | 0.01099 |  |
| DNPH1 | 2368.4 | 0.69 | 0.14104 | 4.8902 | 1.01E-06 |  |
| ADCK3 | 819.277 | 0.69 | 0.21556 | 3.1993 | 0.00138 |  |
| FBXL19-AS1 | 502.841 | 0.69 | 0.18876 | 3.65299 | 0.00026 |  |
| F2 | 925.855 | 0.69 | 0.21443 | 3.20773 | 0.00134 |  |
| RAB40B | 644.388 | 0.69 | 0.15585 | 4.40964 | 1.04E-05 |  |
| SPINK5 | 1530.81 | 0.69 | 0.22587 | 3.03639 | 0.00239 |  |
| PHEX | 130.46 | 0.69 | 0.27804 | 2.46596 | 0.01366 |  |
| AKR7A3 | 2722.1 | 0.69 | 0.20615 | 3.32568 | 0.00088 |  |
| FRAT2 | 151.103 | 0.69 | 0.26053 | 2.63074 | 0.00852 |  |
| KRTCAP3 | 1299.99 | 0.68 | 0.24637 | 2.77957 | 0.00544 |  |
| DTX3L | 4163.67 | 0.68 | 0.18535 | 3.69435 | 0.00022 |  |
| EXO1 | 624.981 | 0.68 | 0.24804 | 2.75691 | 0.00584 |  |
| SASS6 | 215.742 | 0.68 | 0.23699 | 2.88444 | 0.00392 |  |
| MAD2L1 | 879.267 | 0.68 | 0.14074 | 4.84722 | 1.25E-06 |  |
| HELZ2 | 4878.42 | 0.68 | 0.28362 | 2.40462 | 0.01619 |  |
| PBK | 607.746 | 0.68 | 0.15363 | 4.43056 | 9.40E-06 |  |
| SEMA3F | 638.948 | 0.68 | 0.23288 | 2.92004 | 0.0035 |  |
| FABP5 | 909.251 | 0.68 | 0.20527 | 3.30786 | 0.00094 |  |
| ZC3HAV1 | 3868.44 | 0.68 | 0.13299 | 5.09404 | 3.51E-07 |  |
| SPDEF | 785.021 | 0.68 | 0.23689 | 2.85886 | 0.00425 |  |
| C2orf72 | 2067.9 | 0.68 | 0.18831 | 3.59173 | 0.00033 |  |
| CENPE | 944.946 | 0.68 | 0.18536 | 3.64234 | 0.00027 |  |
| CASP9 | 415.536 | 0.67 | 0.18375 | 3.67228 | 0.00024 |  |
| PDCD4 | 910.063 | 0.67 | 0.13343 | 5.05319 | 4.35E-07 |  |
| UBXN8 | 364.459 | 0.67 | 0.16269 | 4.14029 | 3.47E-05 |  |
| CDKN3 | 820.611 | 0.67 | 0.20629 | 3.26456 | 0.0011 |  |
| BAHCC1 | 823.075 | 0.67 | 0.21998 | 3.06061 | 0.00221 |  |
| SP140L | 641.666 | 0.67 | 0.17392 | 3.86665 | 0.00011 |  |
| TFF3 | 5333.8 | 0.67 | 0.23383 | 2.87315 | 0.00406 |  |
| DPP4 | 1204.49 | 0.67 | 0.24405 | 2.75192 | 0.00592 |  |
| SP110 | 1798.91 | 0.67 | 0.19662 | 3.40993 | 0.00065 |  |
| FAM96A | 839.018 | 0.67 | 0.17839 | 3.75476 | 0.00017 |  |
| SULT1A1 | 1825.62 | 0.67 | 0.18967 | 3.52314 | 0.00043 |  |
| HLA-E | 10436.1 | 0.67 | 0.17438 | 3.82743 | 0.00013 |  |
| RFC3 | 621.755 | 0.67 | 0.178 | 3.74857 | 0.00018 |  |
| GGCT | 720.103 | 0.67 | 0.17482 | 3.81596 | 0.00014 |  |
| SLC17A9 | 3226.96 | 0.67 | 0.1338 | 4.97497 | 6.53E-07 |  |
| HNF4G | 707.272 | 0.67 | 0.18759 | 3.54701 | 0.00039 |  |
| LGALS3BP | 23691.6 | 0.66 | 0.17806 | 3.7339 | 0.00019 |  |
| ZDHHC11 | 347.792 | 0.66 | 0.24789 | 2.68041 | 0.00735 |  |
| MGST2 | 512.976 | 0.66 | 0.17576 | 3.77979 | 0.00016 |  |
| DAPP1 | 129.734 | 0.66 | 0.25901 | 2.56281 | 0.01038 |  |
| ASF1B | 722.581 | 0.66 | 0.15521 | 4.25803 | 2.06E-05 |  |
| CLDN3 | 5107.29 | 0.66 | 0.12727 | 5.18284 | 2.19E-07 |  |
| TSPO2 | 92.4778 | 0.66 | 0.2403 | 2.74028 | 0.00614 |  |
| ZNF485 | 89.0672 | 0.66 | 0.25957 | 2.53498 | 0.01125 |  |
| TC2N | 1141.15 | 0.66 | 0.21616 | 3.04161 | 0.00235 |  |
| GSTO2 | 527.619 | 0.66 | 0.26959 | 2.4335 | 0.01495 |  |
| WASF1 | 177.561 | 0.65 | 0.23795 | 2.75011 | 0.00596 |  |
| MAPK12 | 525.693 | 0.65 | 0.17734 | 3.68688 | 0.00023 |  |
| PARM1 | 1185.13 | 0.65 | 0.19694 | 3.31727 | 0.00091 |  |
| RPGR | 261.984 | 0.65 | 0.21422 | 3.0472 | 0.00231 |  |
| NT5C3A | 1173.44 | 0.65 | 0.19876 | 3.28266 | 0.00103 |  |
| CHEK2 | 169.78 | 0.65 | 0.19984 | 3.25882 | 0.00112 |  |
| MIS18A | 465.496 | 0.65 | 0.19237 | 3.38119 | 0.00072 |  |
| STAT2 | 2349.61 | 0.65 | 0.14855 | 4.37781 | 1.20E-05 |  |
| FAM111A | 2452.92 | 0.65 | 0.15472 | 4.19816 | 2.69E-05 |  |
| C2orf74 | 387.276 | 0.65 | 0.18159 | 3.57573 | 0.00035 |  |
| BMP7 | 939.049 | 0.65 | 0.25256 | 2.56391 | 0.01035 |  |
| POLR2K | 1154.46 | 0.65 | 0.13199 | 4.89742 | 9.71E-07 |  |
| ZMAT1 | 348.297 | 0.65 | 0.23689 | 2.72862 | 0.00636 |  |
| MTRNR2L6 | 1365.56 | 0.65 | 0.13687 | 4.71912 | 2.37E-06 |  |
| PRR26 | 1565.2 | 0.65 | 0.12999 | 4.96731 | 6.79E-07 |  |
| CCDC159 | 176.65 | 0.65 | 0.22109 | 2.91948 | 0.00351 |  |
| PCNA | 1010.76 | 0.65 | 0.16664 | 3.87318 | 0.00011 |  |
| ZDHHC14 | 217.59 | 0.64 | 0.26375 | 2.44326 | 0.01456 |  |
| CD82 | 1474.15 | 0.64 | 0.2617 | 2.462 | 0.01382 |  |
| RPS6KL1 | 219.404 | 0.64 | 0.26884 | 2.39384 | 0.01667 |  |
| SSTR5 | 521.47 | 0.64 | 0.20763 | 3.09842 | 0.00195 |  |
| ARRDC1-AS1 | 319.351 | 0.64 | 0.18012 | 3.57023 | 0.00036 |  |
| LACTB2 | 531.94 | 0.64 | 0.20558 | 3.12721 | 0.00176 |  |
| FGFBP3 | 80.6555 | 0.64 | 0.25377 | 2.53297 | 0.01131 |  |
| GDAP1 | 218.9 | 0.64 | 0.25172 | 2.55259 | 0.01069 |  |
| SAMD12 | 348.081 | 0.64 | 0.25348 | 2.53186 | 0.01135 |  |
| TACC1 | 2688.96 | 0.64 | 0.21402 | 2.99761 | 0.00272 |  |
| NANS | 1587.15 | 0.64 | 0.18425 | 3.48029 | 0.0005 |  |
| TMEM62 | 1015.17 | 0.64 | 0.11428 | 5.6016 | 2.12E-08 |  |
| MNX1 | 408.531 | 0.64 | 0.18175 | 3.52151 | 0.00043 |  |
| RIMKLB | 1788.32 | 0.64 | 0.20917 | 3.05878 | 0.00222 |  |
| FDXR | 297.798 | 0.64 | 0.18674 | 3.42122 | 0.00062 |  |
| RBM38 | 1301.21 | 0.64 | 0.2056 | 3.10649 | 0.00189 |  |
| HS6ST1 | 2056.9 | 0.64 | 0.24367 | 2.6201 | 0.00879 |  |
| SNHG8 | 1980.23 | 0.64 | 0.16181 | 3.93942 | 8.17E-05 |  |
| SPIRE2 | 478.068 | 0.64 | 0.24115 | 2.64304 | 0.00822 |  |
| PPFIA3 | 273.803 | 0.64 | 0.17651 | 3.60844 | 0.00031 |  |
| SLC18B1 | 310.153 | 0.64 | 0.19127 | 3.32823 | 0.00087 |  |
| BAG2 | 1014.5 | 0.64 | 0.13316 | 4.779 | 1.76E-06 |  |
| SLC43A2 | 520.016 | 0.64 | 0.20928 | 3.04075 | 0.00236 |  |
| CDC7 | 284.557 | 0.64 | 0.18654 | 3.40924 | 0.00065 |  |
| ABCC5 | 1506.1 | 0.63 | 0.14727 | 4.30899 | 1.64E-05 |  |
| LOC440028 | 118.846 | 0.63 | 0.25958 | 2.43881 | 0.01474 |  |
| A1CF | 403.468 | 0.63 | 0.23011 | 2.74958 | 0.00597 |  |
| SFXN5 | 890.076 | 0.63 | 0.17713 | 3.57107 | 0.00036 |  |
| PDZD3 | 543.561 | 0.63 | 0.21021 | 3.0083 | 0.00263 |  |
| SPATA13 | 955.709 | 0.63 | 0.15088 | 4.18881 | 2.80E-05 |  |
| FAM195A | 699.251 | 0.63 | 0.13277 | 4.75769 | 1.96E-06 |  |
| HNF1A-AS1 | 1340.66 | 0.63 | 0.2354 | 2.68155 | 0.00733 |  |
| TINAG | 454.763 | 0.63 | 0.2351 | 2.67874 | 0.00739 |  |
| PGBD2 | 154.44 | 0.63 | 0.23515 | 2.67699 | 0.00743 |  |
| ACVR1B | 1247.2 | 0.63 | 0.19098 | 3.29492 | 0.00098 |  |
| FOPNL | 1514.41 | 0.63 | 0.17998 | 3.48027 | 0.0005 |  |
| PXMP2 | 356.089 | 0.63 | 0.18607 | 3.36152 | 0.00078 |  |
| CXCL16 | 3825.82 | 0.62 | 0.12604 | 4.94813 | 7.49E-07 |  |
| FN3K | 1481.92 | 0.62 | 0.16534 | 3.77182 | 0.00016 |  |
| RBBP8 | 642.69 | 0.62 | 0.17468 | 3.56413 | 0.00037 |  |
| LARP7 | 2160.72 | 0.62 | 0.2192 | 2.82767 | 0.00469 |  |
| PSME1 | 2516.63 | 0.62 | 0.14156 | 4.37596 | 1.21E-05 |  |
| PIGP | 566.718 | 0.62 | 0.15295 | 4.04732 | 5.18E-05 |  |
| LHPP | 1407.63 | 0.62 | 0.12085 | 5.12223 | 3.02E-07 |  |
| PNPO | 1481.69 | 0.62 | 0.11615 | 5.32538 | 1.01E-07 |  |
| HOXA5 | 95.7397 | 0.62 | 0.23829 | 2.59299 | 0.00951 |  |
| STAT5A | 200.698 | 0.62 | 0.21725 | 2.83743 | 0.00455 |  |
| LHX4-AS1 | 163.42 | 0.62 | 0.23676 | 2.60218 | 0.00926 |  |
| PCBD1 | 3683.62 | 0.62 | 0.11964 | 5.14888 | 2.62E-07 |  |
| SOWAHB | 171.151 | 0.62 | 0.23465 | 2.62244 | 0.00873 |  |
| PABPN1 | 348.066 | 0.61 | 0.17708 | 3.47157 | 0.00052 |  |
| C17orf97 | 254.12 | 0.61 | 0.18183 | 3.38058 | 0.00072 |  |
| ALDH16A1 | 1275.29 | 0.61 | 0.15667 | 3.91948 | 8.87E-05 |  |
| PPARGC1B | 354.389 | 0.61 | 0.20461 | 2.99993 | 0.0027 |  |
| MDK | 12741.2 | 0.61 | 0.16481 | 3.7234 | 0.0002 |  |
| ZDHHC23 | 435.125 | 0.61 | 0.15142 | 4.05132 | 5.09E-05 |  |
| GPD1L | 718.404 | 0.61 | 0.20365 | 3.00908 | 0.00262 |  |
| C10orf32 | 492.894 | 0.61 | 0.21876 | 2.79875 | 0.00513 |  |
| CEP126 | 342.454 | 0.61 | 0.25163 | 2.43202 | 0.01501 |  |
| THTPA | 299.952 | 0.61 | 0.18364 | 3.33186 | 0.00086 |  |
| FIGNL1 | 510.978 | 0.61 | 0.18042 | 3.38924 | 0.0007 |  |
| SPSB2 | 823.554 | 0.61 | 0.19473 | 3.1199 | 0.00181 |  |
| FAM107B | 3146.06 | 0.61 | 0.18433 | 3.29548 | 0.00098 |  |
| KCNK5 | 2245.1 | 0.61 | 0.15337 | 3.94545 | 7.96E-05 |  |
| PBLD | 884.286 | 0.61 | 0.1964 | 3.08073 | 0.00206 |  |
| SFXN4 | 775.634 | 0.60 | 0.13043 | 4.63698 | 3.54E-06 |  |
| RNASEH2C | 546.058 | 0.60 | 0.16286 | 3.71251 | 0.00021 |  |
| NUF2 | 335.28 | 0.60 | 0.19407 | 3.11017 | 0.00187 |  |
| DLGAP1-AS1 | 234.588 | 0.60 | 0.18991 | 3.1781 | 0.00148 |  |
| GMNN | 643.73 | 0.60 | 0.15848 | 3.80456 | 0.00014 |  |
| NUCB2 | 627.938 | 0.60 | 0.22789 | 2.64278 | 0.00822 |  |
| ORMDL1 | 917.708 | 0.60 | 0.20924 | 2.87566 | 0.00403 |  |
| BSPRY | 871.132 | 0.60 | 0.14466 | 4.15924 | 3.19E-05 |  |
| NDUFAF4 | 257.953 | 0.60 | 0.17667 | 3.40546 | 0.00066 |  |
| SHROOM1 | 753.778 | 0.60 | 0.21766 | 2.76271 | 0.00573 |  |
| C5orf34 | 124.625 | 0.60 | 0.25195 | 2.38476 | 0.01709 |  |
| METRNL | 1106.86 | 0.60 | 0.19744 | 3.04287 | 0.00234 |  |
| TMEM51 | 628.947 | 0.60 | 0.1926 | 3.11592 | 0.00183 |  |
| FOXRED2 | 486.131 | 0.60 | 0.22717 | 2.64159 | 0.00825 |  |
| ATP10D | 292.011 | 0.60 | 0.2384 | 2.51423 | 0.01193 |  |
| PLK4 | 608.231 | 0.60 | 0.15629 | 3.82887 | 0.00013 |  |
| LOC100507437 | 114.528 | 0.60 | 0.23611 | 2.53222 | 0.01133 |  |
| HIBCH | 795.211 | 0.60 | 0.20069 | 2.97788 | 0.0029 |  |
| RMI2 | 624.585 | 0.60 | 0.14912 | 4.00644 | 6.16E-05 |  |
| PNKD | 10418 | 0.60 | 0.12215 | 4.88696 | 1.02E-06 |  |
| TOE1 | 322.867 | 0.60 | 0.18214 | 3.27725 | 0.00105 |  |
| LOC101928738 | 187.88 | 0.60 | 0.24788 | 2.40655 | 0.0161 |  |
| ADAP1 | 4002.37 | 0.60 | 0.16395 | 3.63495 | 0.00028 |  |
| BRCA1 | 887.263 | 0.60 | 0.17508 | 3.40303 | 0.00067 |  |
| LOH12CR1 | 295.801 | 0.59 | 0.15806 | 3.76096 | 0.00017 |  |
| MRPS26 | 570.985 | 0.59 | 0.152 | 3.89899 | 9.66E-05 |  |
| ALDH18A1 | 2502.26 | 0.59 | 0.141 | 4.19611 | 2.72E-05 |  |
| FKBP11 | 798.395 | 0.59 | 0.13515 | 4.37323 | 1.22E-05 |  |
| TPSG1 | 704.789 | 0.59 | 0.23091 | 2.55616 | 0.01058 |  |
| COA3 | 1595.73 | 0.59 | 0.17195 | 3.43072 | 0.0006 |  |
| NDUFB5 | 1007.81 | 0.59 | 0.15642 | 3.76954 | 0.00016 |  |
| FMO5 | 560.929 | 0.59 | 0.23701 | 2.48427 | 0.01298 |  |
| HYLS1 | 518.368 | 0.59 | 0.13961 | 4.21261 | 2.52E-05 |  |
| CFDP1 | 1581.97 | 0.59 | 0.1333 | 4.41026 | 1.03E-05 |  |
| LSM3 | 696.563 | 0.59 | 0.17408 | 3.37711 | 0.00073 |  |
| AGR3 | 643.426 | 0.59 | 0.15561 | 3.77522 | 0.00016 |  |
| EPHA10 | 349.307 | 0.59 | 0.22527 | 2.60742 | 0.00912 |  |
| ZNF688 | 266.772 | 0.59 | 0.21217 | 2.76734 | 0.00565 |  |
| ATP5J | 1817.98 | 0.59 | 0.13759 | 4.26487 | 2.00E-05 |  |
| VWA7 | 496.826 | 0.59 | 0.20302 | 2.88981 | 0.00385 |  |
| AGPAT9 | 1204.55 | 0.59 | 0.23231 | 2.52312 | 0.01163 |  |
| NSUN6 | 336.6 | 0.59 | 0.18251 | 3.20938 | 0.00133 |  |
| LSM4 | 2278.92 | 0.59 | 0.0965 | 6.06894 | 1.29E-09 |  |
| SPAG1 | 1110.42 | 0.59 | 0.17862 | 3.27828 | 0.00104 |  |
| RTKN2 | 278.806 | 0.59 | 0.21996 | 2.66181 | 0.00777 |  |
| NADK | 2834.42 | 0.59 | 0.1047 | 5.58846 | 2.29E-08 |  |
| BACE2 | 1178.18 | 0.58 | 0.20142 | 2.90301 | 0.0037 |  |
| SUOX | 1027.25 | 0.58 | 0.1486 | 3.92886 | 8.54E-05 |  |
| PARP12 | 1619.2 | 0.58 | 0.21724 | 2.68511 | 0.00725 |  |
| 1-Mar | 790.897 | 0.58 | 0.23297 | 2.50129 | 0.01237 |  |
| SLC39A8 | 516.705 | 0.58 | 0.16933 | 3.4409 | 0.00058 |  |
| LGR4 | 6096.11 | 0.58 | 0.16614 | 3.50594 | 0.00045 |  |
| CD9 | 8847.07 | 0.58 | 0.20215 | 2.86768 | 0.00413 |  |
| AKR7L | 977.558 | 0.58 | 0.19081 | 3.0328 | 0.00242 |  |
| ZSCAN12P1 | 156.478 | 0.58 | 0.23753 | 2.43569 | 0.01486 |  |
| C6orf136 | 571.4 | 0.58 | 0.14611 | 3.95792 | 7.56E-05 |  |
| ANO5 | 260.887 | 0.58 | 0.23355 | 2.47513 | 0.01332 |  |
| KBTBD6 | 380.278 | 0.58 | 0.1745 | 3.30757 | 0.00094 |  |
| RHNO1 | 1067.55 | 0.58 | 0.13366 | 4.31267 | 1.61E-05 |  |
| C9orf142 | 667.149 | 0.58 | 0.16332 | 3.5292 | 0.00042 |  |
| SLC25A45 | 482.288 | 0.58 | 0.20283 | 2.84138 | 0.00449 |  |
| PMS1 | 468.967 | 0.58 | 0.21421 | 2.69003 | 0.00714 |  |
| ABHD3 | 367.993 | 0.58 | 0.1944 | 2.9625 | 0.00305 |  |
| C15orf62 | 211.717 | 0.58 | 0.23358 | 2.46492 | 0.0137 |  |
| PSIP1 | 949.954 | 0.58 | 0.14646 | 3.9271 | 8.60E-05 |  |
| DDC | 1506.31 | 0.57 | 0.20536 | 2.79193 | 0.00524 |  |
| EME1 | 292.977 | 0.57 | 0.19777 | 2.89448 | 0.0038 |  |
| HRSP12 | 222.108 | 0.57 | 0.19657 | 2.91201 | 0.00359 |  |
| DEPDC1B | 407.563 | 0.57 | 0.21638 | 2.63426 | 0.00843 |  |
| UBE2C | 1666.97 | 0.57 | 0.13891 | 4.0912 | 4.29E-05 |  |
| RPGRIP1L | 218.096 | 0.57 | 0.22136 | 2.56277 | 0.01038 |  |
| LOC101928766 | 104.234 | 0.57 | 0.2382 | 2.38111 | 0.01726 |  |
| CCNG1 | 840.668 | 0.57 | 0.12883 | 4.39816 | 1.09E-05 |  |
| UBE2S | 2861.81 | 0.57 | 0.13913 | 4.07179 | 4.67E-05 |  |
| H2AFX | 3672.97 | 0.57 | 0.09913 | 5.71374 | 1.11E-08 |  |
| HMGN3 | 427.055 | 0.57 | 0.18273 | 3.09621 | 0.00196 |  |
| TMEM180 | 159.882 | 0.56 | 0.21 | 2.68673 | 0.00722 |  |
| FLJ32255 | 116.575 | 0.56 | 0.23212 | 2.42592 | 0.01527 |  |
| STAU2 | 968.01 | 0.56 | 0.22318 | 2.52296 | 0.01164 |  |
| PI4K2B | 715.363 | 0.56 | 0.17168 | 3.27842 | 0.00104 |  |
| EXOC6 | 415.793 | 0.56 | 0.16274 | 3.45821 | 0.00054 |  |
| CISD2 | 1152.46 | 0.56 | 0.13872 | 4.05672 | 4.98E-05 |  |
| CDC25A | 199.099 | 0.56 | 0.21435 | 2.62495 | 0.00867 |  |
| NR2F2 | 2465.69 | 0.56 | 0.1473 | 3.81871 | 0.00013 |  |
| SERINC4 | 141.807 | 0.56 | 0.21015 | 2.67546 | 0.00746 |  |
| TAF6L | 407.156 | 0.56 | 0.18872 | 2.97364 | 0.00294 |  |
| IL6R | 958.63 | 0.56 | 0.17106 | 3.27961 | 0.00104 |  |
| CDCA5 | 1428.23 | 0.56 | 0.16652 | 3.36781 | 0.00076 |  |
| DCAKD | 930.471 | 0.56 | 0.13136 | 4.26581 | 1.99E-05 |  |
| SGOL2 | 574.887 | 0.56 | 0.16279 | 3.44151 | 0.00058 |  |
| SLFN5 | 865.381 | 0.56 | 0.21537 | 2.59863 | 0.00936 |  |
| C9orf40 | 384.198 | 0.56 | 0.16333 | 3.42571 | 0.00061 |  |
| DANCR | 602.932 | 0.56 | 0.18719 | 2.98431 | 0.00284 |  |
| TMEM263 | 1382.95 | 0.56 | 0.20318 | 2.74894 | 0.00598 |  |
| GSTA4 | 470.153 | 0.56 | 0.1947 | 2.86268 | 0.0042 |  |
| CAMKMT | 118.459 | 0.56 | 0.22443 | 2.4828 | 0.01304 |  |
| NAT1 | 237.98 | 0.56 | 0.19556 | 2.84906 | 0.00438 |  |
| CEBPD | 862.959 | 0.56 | 0.19544 | 2.85046 | 0.00437 |  |
| CMBL | 5267.69 | 0.56 | 0.23388 | 2.3792 | 0.01735 |  |
| MYO1A | 2730.28 | 0.56 | 0.19485 | 2.8539 | 0.00432 |  |
| CYCS | 3487.38 | 0.55 | 0.11647 | 4.76451 | 1.89E-06 |  |
| MANSC1 | 800.508 | 0.55 | 0.1735 | 3.19551 | 0.0014 |  |
| MAP9 | 900.609 | 0.55 | 0.23027 | 2.40691 | 0.01609 |  |
| NEU4 | 2920.67 | 0.55 | 0.14552 | 3.80837 | 0.00014 |  |
| HJURP | 2751.47 | 0.55 | 0.20491 | 2.70111 | 0.00691 |  |
| MUTYH | 252.705 | 0.55 | 0.21447 | 2.57621 | 0.00999 |  |
| FAM178A | 1126.05 | 0.55 | 0.15651 | 3.52835 | 0.00042 |  |
| POLQ | 616.442 | 0.55 | 0.2022 | 2.72833 | 0.00637 |  |
| SLC39A5 | 2216.84 | 0.55 | 0.12914 | 4.27168 | 1.94E-05 |  |
| MAP3K10 | 265.82 | 0.55 | 0.18835 | 2.92068 | 0.00349 |  |
| GOT1 | 1227.9 | 0.55 | 0.15264 | 3.60309 | 0.00031 |  |
| DNA2 | 362.501 | 0.55 | 0.20183 | 2.72477 | 0.00643 |  |
| NDUFA7 | 595.92 | 0.55 | 0.14987 | 3.66893 | 0.00024 |  |
| SMIM22 | 1393.27 | 0.55 | 0.11538 | 4.76229 | 1.91E-06 |  |
| LYSMD1 | 511.604 | 0.55 | 0.15475 | 3.55018 | 0.00038 |  |
| CLSPN | 687.484 | 0.55 | 0.19889 | 2.75724 | 0.00583 |  |
| GRB14 | 441.01 | 0.55 | 0.16118 | 3.40106 | 0.00067 |  |
| FEN1 | 1948.42 | 0.55 | 0.15006 | 3.65165 | 0.00026 |  |
| KIAA1524 | 440.898 | 0.55 | 0.17415 | 3.13974 | 0.00169 |  |
| POC1A | 281.201 | 0.55 | 0.19104 | 2.86049 | 0.00423 |  |
| EFHD2 | 4399.48 | 0.55 | 0.12796 | 4.27004 | 1.95E-05 |  |
| GOLGA2P5 | 304.003 | 0.55 | 0.19654 | 2.77914 | 0.00545 |  |
| CEP55 | 1832.75 | 0.55 | 0.13172 | 4.14297 | 3.43E-05 |  |
| CENPA | 316.261 | 0.55 | 0.15557 | 3.50607 | 0.00045 |  |
| POR | 4413.02 | 0.55 | 0.1879 | 2.9014 | 0.00371 |  |
| EPHX2 | 416.474 | 0.54 | 0.1652 | 3.29801 | 0.00097 |  |
| KIF12 | 1442.59 | 0.54 | 0.17022 | 3.20052 | 0.00137 |  |
| ING3 | 280.589 | 0.54 | 0.19725 | 2.76138 | 0.00576 |  |
| DDX10 | 939.573 | 0.54 | 0.12864 | 4.23063 | 2.33E-05 |  |
| LOC100130238 | 524.81 | 0.54 | 0.1652 | 3.29406 | 0.00099 |  |
| PRSS16 | 1056.17 | 0.54 | 0.11241 | 4.82774 | 1.38E-06 |  |
| RAB3IP | 1899.17 | 0.54 | 0.15643 | 3.46661 | 0.00053 |  |
| RARG | 334.376 | 0.54 | 0.18686 | 2.90075 | 0.00372 |  |
| KIF11 | 1456.73 | 0.54 | 0.20342 | 2.6646 | 0.00771 |  |
| DNAJC30 | 588.467 | 0.54 | 0.14367 | 3.77216 | 0.00016 |  |
| HELLS | 474.901 | 0.54 | 0.19644 | 2.75529 | 0.00586 |  |
| NAPA | 2793.62 | 0.54 | 0.12864 | 4.20688 | 2.59E-05 |  |
| AP1S1 | 2733.26 | 0.54 | 0.1151 | 4.69392 | 2.68E-06 |  |
| HDHD2 | 555.879 | 0.54 | 0.15121 | 3.56472 | 0.00036 |  |
| NFIC | 2062.08 | 0.54 | 0.19804 | 2.71953 | 0.00654 |  |
| ASPM | 1841.5 | 0.54 | 0.17387 | 3.09606 | 0.00196 |  |
| GAL3ST1 | 2555.08 | 0.54 | 0.17528 | 3.06988 | 0.00214 |  |
| LYAR | 878.032 | 0.54 | 0.17354 | 3.09939 | 0.00194 |  |
| STRADB | 572.154 | 0.54 | 0.20359 | 2.63935 | 0.00831 |  |
| DHFR | 2388.48 | 0.54 | 0.13556 | 3.96352 | 7.39E-05 |  |
| ACBD4 | 553.641 | 0.54 | 0.14765 | 3.63743 | 0.00028 |  |
| SPTSSA | 548.056 | 0.54 | 0.14405 | 3.72754 | 0.00019 |  |
| TMEM70 | 642.517 | 0.54 | 0.15254 | 3.51588 | 0.00044 |  |
| XBP1 | 3454.11 | 0.54 | 0.10032 | 5.34191 | 9.20E-08 |  |
| PTTG1 | 803.454 | 0.54 | 0.16079 | 3.32862 | 0.00087 |  |
| CALML4 | 3307.25 | 0.53 | 0.17736 | 3.0161 | 0.00256 |  |
| PWWP2B | 835.569 | 0.53 | 0.18776 | 2.8482 | 0.0044 |  |
| LYRM5 | 215.957 | 0.53 | 0.21009 | 2.54434 | 0.01095 |  |
| SMIM11 | 449.843 | 0.53 | 0.17228 | 3.10191 | 0.00192 |  |
| MIEN1 | 855.735 | 0.53 | 0.12265 | 4.34259 | 1.41E-05 |  |
| ANAPC13 | 1504.65 | 0.53 | 0.10472 | 5.08071 | 3.76E-07 |  |
| F8 | 190.156 | 0.53 | 0.20324 | 2.61606 | 0.0089 |  |
| PPARA | 2175.28 | 0.53 | 0.10844 | 4.90263 | 9.46E-07 |  |
| MRPL4 | 1382.49 | 0.53 | 0.14576 | 3.6475 | 0.00026 |  |
| AGMAT | 298.013 | 0.53 | 0.213 | 2.49504 | 0.01259 |  |
| HPN | 2527.11 | 0.53 | 0.16538 | 3.19875 | 0.00138 |  |
| ARHGEF2 | 6997.8 | 0.53 | 0.1417 | 3.73192 | 0.00019 |  |
| NDC80 | 222.603 | 0.53 | 0.20722 | 2.5509 | 0.01074 |  |
| ACADM | 379.395 | 0.53 | 0.16157 | 3.2699 | 0.00108 |  |
| CAMK1D | 1203.42 | 0.53 | 0.17417 | 3.03253 | 0.00243 |  |
| LINC01184 | 346.981 | 0.53 | 0.15863 | 3.32404 | 0.00089 |  |
| NCAPG | 762.855 | 0.53 | 0.15753 | 3.33615 | 0.00085 |  |
| TMEM125 | 758.553 | 0.53 | 0.18522 | 2.83493 | 0.00458 |  |
| LRIG3 | 150.902 | 0.52 | 0.21862 | 2.39799 | 0.01649 |  |
| FOXD2 | 128.119 | 0.52 | 0.21412 | 2.44748 | 0.01439 |  |
| ZWILCH | 491.962 | 0.52 | 0.17335 | 3.01926 | 0.00253 |  |
| NBN | 1378.02 | 0.52 | 0.1239 | 4.22236 | 2.42E-05 |  |
| DUT | 1695.45 | 0.52 | 0.12989 | 4.01964 | 5.83E-05 |  |
| HAGLR | 734.191 | 0.52 | 0.21197 | 2.46225 | 0.01381 |  |
| C1orf122 | 410.18 | 0.52 | 0.18675 | 2.79257 | 0.00523 |  |
| DHTKD1 | 1443.83 | 0.52 | 0.10937 | 4.76496 | 1.89E-06 |  |
| TMEM106B | 1743.41 | 0.52 | 0.13316 | 3.91035 | 9.22E-05 |  |
| DSN1 | 717.845 | 0.52 | 0.13151 | 3.956 | 7.62E-05 |  |
| PTP4A1 | 9250.39 | 0.52 | 0.13197 | 3.9413 | 8.10E-05 |  |
| FNIP2 | 688.092 | 0.52 | 0.14635 | 3.55258 | 0.00038 |  |
| TMPO | 4148.01 | 0.52 | 0.13037 | 3.98766 | 6.67E-05 |  |
| SLC18A2 | 671.23 | 0.52 | 0.12969 | 4.0056 | 6.19E-05 |  |
| EEF1A2 | 4804.52 | 0.52 | 0.20957 | 2.47394 | 0.01336 |  |
| SLC43A3 | 739.047 | 0.52 | 0.12554 | 4.12621 | 3.69E-05 |  |
| CBR4 | 582.103 | 0.52 | 0.16037 | 3.22235 | 0.00127 |  |
| FDX1 | 805.678 | 0.52 | 0.16815 | 3.07216 | 0.00213 |  |
| C11orf54 | 963.218 | 0.52 | 0.16438 | 3.14221 | 0.00168 |  |
| RAD51AP1 | 399.53 | 0.52 | 0.17258 | 2.99031 | 0.00279 |  |
| TMEM38B | 248.4 | 0.52 | 0.17833 | 2.89253 | 0.00382 |  |
| HINT2 | 813.909 | 0.52 | 0.17519 | 2.94435 | 0.00324 |  |
| SFMBT2 | 327.066 | 0.52 | 0.18418 | 2.79957 | 0.00512 |  |
| CEP290 | 940.786 | 0.52 | 0.18381 | 2.80256 | 0.00507 |  |
| CAT | 1241.83 | 0.51 | 0.17946 | 2.86622 | 0.00415 |  |
| PANK1 | 167.945 | 0.51 | 0.20563 | 2.48932 | 0.0128 |  |
| OCIAD1 | 2457.99 | 0.51 | 0.1557 | 3.28559 | 0.00102 |  |
| MPHOSPH9 | 531.663 | 0.51 | 0.16988 | 3.00664 | 0.00264 |  |
| ACTR6 | 304.323 | 0.51 | 0.20899 | 2.44318 | 0.01456 |  |
| MYO3A | 131.869 | 0.51 | 0.21311 | 2.39504 | 0.01662 |  |
| ETFB | 6386.57 | 0.51 | 0.1908 | 2.67114 | 0.00756 |  |
| CHAF1B | 665.515 | 0.51 | 0.18438 | 2.76295 | 0.00573 |  |
| TCF19 | 1082.95 | 0.51 | 0.1305 | 3.90344 | 9.48E-05 |  |
| CKAP4 | 5312.36 | 0.51 | 0.12105 | 4.20716 | 2.59E-05 |  |
| FAM104B | 383.306 | 0.51 | 0.15526 | 3.27843 | 0.00104 |  |
| GPR180 | 426.19 | 0.51 | 0.17387 | 2.92585 | 0.00344 |  |
| COMMD8 | 231.123 | 0.51 | 0.18925 | 2.68743 | 0.0072 |  |
| FAS | 305.959 | 0.51 | 0.20551 | 2.47385 | 0.01337 |  |
| TOR3A | 806.238 | 0.51 | 0.11292 | 4.50029 | 6.79E-06 |  |
| CENPU | 845.295 | 0.51 | 0.16682 | 3.04563 | 0.00232 |  |
| RNASEH2B | 500.708 | 0.51 | 0.15008 | 3.382 | 0.00072 |  |
| APOL1 | 1000.22 | 0.51 | 0.14378 | 3.52927 | 0.00042 |  |
| ADAL | 280.139 | 0.51 | 0.15983 | 3.17224 | 0.00151 |  |
| SLC9A3R1 | 4821.85 | 0.51 | 0.10484 | 4.83027 | 1.36E-06 |  |
| MAGOHB | 516.042 | 0.51 | 0.13559 | 3.72948 | 0.00019 |  |
| IMPA2 | 1826.23 | 0.51 | 0.12436 | 4.06293 | 4.85E-05 |  |
| CDCA3 | 554.145 | 0.50 | 0.20643 | 2.44514 | 0.01448 |  |
| SPAG16 | 584.421 | 0.50 | 0.16339 | 3.08761 | 0.00202 |  |
| ATPIF1 | 1687.39 | 0.50 | 0.11956 | 4.21889 | 2.46E-05 |  |
| CCDC88B | 318.465 | 0.50 | 0.19382 | 2.60146 | 0.00928 |  |
| CDH17 | 22736.5 | 0.50 | 0.18631 | 2.7061 | 0.00681 |  |
| BAIAP2L2 | 2706.54 | 0.50 | 0.14168 | 3.55716 | 0.00037 |  |
| C1GALT1C1 | 546.047 | 0.50 | 0.18411 | 2.73584 | 0.00622 |  |
| IRF2BP1 | 1138.97 | 0.50 | 0.14431 | 3.48892 | 0.00048 |  |
| S100P | 18218.7 | 0.50 | 0.15129 | 3.32154 | 0.0009 |  |
| MDM2 | 2182.12 | 0.50 | 0.14701 | 3.41757 | 0.00063 |  |
| BLVRA | 1215.14 | 0.50 | 0.12268 | 4.09341 | 4.25E-05 |  |
| C2CD5 | 915.572 | 0.50 | 0.15299 | 3.28197 | 0.00103 |  |
| TDP2 | 5772.18 | 0.50 | 0.11667 | 4.30246 | 1.69E-05 |  |
| HEXDC | 688.111 | 0.50 | 0.13296 | 3.7714 | 0.00016 |  |
| KCNK1 | 453.57 | 0.50 | 0.17604 | 2.8418 | 0.00449 |  |
| RFC4 | 850.832 | 0.50 | 0.17213 | 2.90492 | 0.00367 |  |
| SLCO1B3 | 959.981 | -0.50 | 0.14668 | -3.4116 | 0.00065 |  |
| STXBP1 | 863.714 | -0.50 | 0.1905 | -2.6272 | 0.00861 |  |
| SPTAN1 | 14678.8 | -0.50 | 0.11028 | -4.5394 | 5.64E-06 |  |
| TULP4 | 523.688 | -0.50 | 0.13621 | -3.6775 | 0.00024 |  |
| FBXO32 | 214.268 | -0.50 | 0.2085 | -2.4077 | 0.01605 |  |
| TRIM47 | 3469.33 | -0.50 | 0.17093 | -2.9423 | 0.00326 |  |
| RTN4 | 8644.86 | -0.50 | 0.14297 | -3.5189 | 0.00043 |  |
| ATL1 | 372.289 | -0.50 | 0.18936 | -2.6596 | 0.00782 |  |
| ZNF417 | 223.168 | -0.50 | 0.19914 | -2.5296 | 0.01142 |  |
| LAMB2 | 4058.89 | -0.50 | 0.19737 | -2.5577 | 0.01054 |  |
| RBFOX2 | 1451.77 | -0.51 | 0.13588 | -3.7191 | 0.0002 |  |
| KRT7 | 32710.5 | -0.51 | 0.17206 | -2.9372 | 0.00331 |  |
| COBL | 3043.35 | -0.51 | 0.20917 | -2.4185 | 0.01558 |  |
| PEAK1 | 299.892 | -0.51 | 0.18226 | -2.7758 | 0.00551 |  |
| FARP2 | 572.883 | -0.51 | 0.21086 | -2.3997 | 0.01641 |  |
| ACOX3 | 359.569 | -0.51 | 0.17478 | -2.8991 | 0.00374 |  |
| CELSR1 | 6477.97 | -0.51 | 0.19393 | -2.6138 | 0.00895 |  |
| APP | 15415.6 | -0.51 | 0.15931 | -3.1855 | 0.00144 |  |
| TMEM63B | 1098.37 | -0.51 | 0.19949 | -2.5447 | 0.01094 |  |
| PLXNA1 | 3615.45 | -0.51 | 0.18686 | -2.7192 | 0.00654 |  |
| NOCT | 402.292 | -0.51 | 0.17248 | -2.9464 | 0.00322 |  |
| TLCD2 | 2734.38 | -0.51 | 0.16396 | -3.1017 | 0.00192 |  |
| ARHGEF17 | 1736.2 | -0.51 | 0.15405 | -3.3069 | 0.00094 |  |
| SLC3A2 | 12918.8 | -0.51 | 0.16009 | -3.1881 | 0.00143 |  |
| SGPL1 | 2403.66 | -0.51 | 0.1577 | -3.2376 | 0.00121 |  |
| APH1B | 189.372 | -0.51 | 0.20435 | -2.4986 | 0.01247 |  |
| PPM1M | 282.205 | -0.51 | 0.21087 | -2.422 | 0.01544 |  |
| HES6 | 235.422 | -0.51 | 0.17708 | -2.8844 | 0.00392 |  |
| TBC1D4 | 450.149 | -0.51 | 0.20993 | -2.4337 | 0.01494 |  |
| ACAT2 | 563.732 | -0.51 | 0.213 | -2.4002 | 0.01639 |  |
| CBL | 1030.04 | -0.51 | 0.16628 | -3.0757 | 0.0021 |  |
| WASF2 | 4837.77 | -0.51 | 0.12821 | -3.9918 | 6.56E-05 |  |
| ACACA | 2449.6 | -0.51 | 0.16777 | -3.0563 | 0.00224 |  |
| CSRNP2 | 638.734 | -0.51 | 0.16822 | -3.0482 | 0.0023 |  |
| MEF2D | 3902.77 | -0.51 | 0.12365 | -4.1476 | 3.36E-05 |  |
| HHLA2 | 1549.16 | -0.51 | 0.1535 | -3.3418 | 0.00083 |  |
| CFLAR | 11718 | -0.51 | 0.19505 | -2.6306 | 0.00852 |  |
| TNIP1 | 12019.5 | -0.51 | 0.11701 | -4.3861 | 1.15E-05 |  |
| TMOD3 | 8311.51 | -0.51 | 0.14829 | -3.4641 | 0.00053 |  |
| CEP112 | 290.214 | -0.51 | 0.1779 | -2.8881 | 0.00388 |  |
| IRF6 | 2725.57 | -0.51 | 0.18063 | -2.845 | 0.00444 |  |
| CD109 | 1051.47 | -0.52 | 0.12905 | -4.001 | 6.31E-05 |  |
| FEM1C | 933.495 | -0.52 | 0.15161 | -3.4081 | 0.00065 |  |
| PPP2R3A | 227.185 | -0.52 | 0.19515 | -2.6479 | 0.0081 |  |
| PTMS | 8170.97 | -0.52 | 0.16773 | -3.0895 | 0.00201 |  |
| IVNS1ABP | 2380.46 | -0.52 | 0.12936 | -4.0072 | 6.14E-05 |  |
| MYO1C | 14092.3 | -0.52 | 0.14325 | -3.619 | 0.0003 |  |
| NEK6 | 4413.3 | -0.52 | 0.2052 | -2.5281 | 0.01147 |  |
| SACS | 995.959 | -0.52 | 0.16841 | -3.0852 | 0.00203 |  |
| TP53I3 | 2767.11 | -0.52 | 0.19011 | -2.7334 | 0.00627 |  |
| PVR | 4777.84 | -0.52 | 0.21527 | -2.4171 | 0.01565 |  |
| SYNE1 | 1374.97 | -0.52 | 0.15797 | -3.2944 | 0.00099 |  |
| PTPN12 | 2558.45 | -0.52 | 0.11596 | -4.5009 | 6.77E-06 |  |
| MIR22HG | 1500.82 | -0.52 | 0.21163 | -2.4664 | 0.01365 |  |
| SHB | 1893.57 | -0.52 | 0.15013 | -3.4769 | 0.00051 |  |
| NRBP2 | 913.419 | -0.52 | 0.19664 | -2.6547 | 0.00794 |  |
| CCSER2 | 902.642 | -0.52 | 0.19687 | -2.6523 | 0.00799 |  |
| FDPS | 4929.18 | -0.52 | 0.13275 | -3.9416 | 8.09E-05 |  |
| SLC7A7 | 4562.62 | -0.52 | 0.18629 | -2.81 | 0.00495 |  |
| DAG1 | 3510.54 | -0.52 | 0.17314 | -3.0317 | 0.00243 |  |
| DCTN2 | 3023.25 | -0.53 | 0.14461 | -3.6383 | 0.00027 |  |
| IQSEC1 | 907.785 | -0.53 | 0.18501 | -2.8443 | 0.00445 |  |
| DENND5B | 1123.59 | -0.53 | 0.18683 | -2.8259 | 0.00472 |  |
| NKX2-5 | 133.665 | -0.53 | 0.21658 | -2.4404 | 0.01467 |  |
| ZNF250 | 363.59 | -0.53 | 0.2036 | -2.5961 | 0.00943 |  |
| C18orf25 | 463.356 | -0.53 | 0.15032 | -3.52 | 0.00043 |  |
| TRIM3 | 201.246 | -0.53 | 0.21682 | -2.4407 | 0.01466 |  |
| WFS1 | 693.451 | -0.53 | 0.21068 | -2.5156 | 0.01188 |  |
| MPP1 | 438.427 | -0.53 | 0.18685 | -2.837 | 0.00455 |  |
| TRIM65 | 1362.55 | -0.53 | 0.13927 | -3.8081 | 0.00014 |  |
| IBA57 | 803.489 | -0.53 | 0.21517 | -2.4668 | 0.01363 |  |
| KLF6 | 8205.21 | -0.53 | 0.19365 | -2.741 | 0.00613 |  |
| RLF | 381.089 | -0.53 | 0.18217 | -2.9143 | 0.00356 |  |
| LINC00641 | 361.874 | -0.53 | 0.21464 | -2.477 | 0.01325 |  |
| MSMO1 | 2506.92 | -0.53 | 0.20472 | -2.6006 | 0.00931 |  |
| PTPN21 | 1761.28 | -0.53 | 0.16588 | -3.2115 | 0.00132 |  |
| PDLIM2 | 2120.73 | -0.53 | 0.16589 | -3.2126 | 0.00132 |  |
| NOD1 | 529.626 | -0.53 | 0.15596 | -3.4217 | 0.00062 |  |
| SNX29 | 388.391 | -0.53 | 0.17499 | -3.0542 | 0.00226 |  |
| RSU1 | 1351.66 | -0.54 | 0.16434 | -3.2573 | 0.00112 |  |
| CSNK2A2 | 465.665 | -0.54 | 0.21948 | -2.4395 | 0.01471 |  |
| ZNF776 | 549.337 | -0.54 | 0.13715 | -3.9099 | 9.24E-05 |  |
| PABPC1 | 18781.6 | -0.54 | 0.12125 | -4.4256 | 9.62E-06 |  |
| IL11RA | 137.768 | -0.54 | 0.21279 | -2.5219 | 0.01167 |  |
| GAREML | 390.155 | -0.54 | 0.19268 | -2.789 | 0.00529 |  |
| IQGAP1 | 7197.41 | -0.54 | 0.1383 | -3.8862 | 0.0001 |  |
| FDFT1 | 4247.55 | -0.54 | 0.16264 | -3.3069 | 0.00094 |  |
| BCAM | 3953.06 | -0.54 | 0.16654 | -3.2535 | 0.00114 |  |
| RNF39 | 135.045 | -0.54 | 0.21922 | -2.4733 | 0.01339 |  |
| FLII | 11360.9 | -0.54 | 0.17171 | -3.1578 | 0.00159 |  |
| MYOM3 | 4358.13 | -0.54 | 0.11096 | -4.8907 | 1.00E-06 |  |
| CLN8 | 1680.98 | -0.54 | 0.11602 | -4.6789 | 2.88E-06 |  |
| FLCN | 1277.55 | -0.54 | 0.14039 | -3.8671 | 0.00011 |  |
| GALNT10 | 3146.84 | -0.54 | 0.2048 | -2.6536 | 0.00796 |  |
| AMFR | 3695.75 | -0.54 | 0.15744 | -3.4526 | 0.00056 |  |
| TPM4 | 18331 | -0.54 | 0.13 | -4.1823 | 2.89E-05 |  |
| CRHR1-IT1 | 149.405 | -0.54 | 0.21774 | -2.5016 | 0.01236 |  |
| GADD45A | 493.813 | -0.55 | 0.14956 | -3.649 | 0.00026 |  |
| SCARNA17 | 279.444 | -0.55 | 0.22451 | -2.4349 | 0.01489 |  |
| MEX3D | 1889.45 | -0.55 | 0.17411 | -3.1505 | 0.00163 |  |
| EEF2 | 33868.8 | -0.55 | 0.16724 | -3.2873 | 0.00101 |  |
| ACVR1 | 1535.19 | -0.55 | 0.13253 | -4.1741 | 2.99E-05 |  |
| DIAPH1 | 9460.07 | -0.55 | 0.15183 | -3.6499 | 0.00026 |  |
| MYO15B | 6893.46 | -0.56 | 0.19284 | -2.884 | 0.00393 |  |
| PLEKHG3 | 3850.49 | -0.56 | 0.18322 | -3.0401 | 0.00237 |  |
| SYT17 | 1880.08 | -0.56 | 0.16095 | -3.4615 | 0.00054 |  |
| STK10 | 633.339 | -0.56 | 0.15076 | -3.7156 | 0.0002 |  |
| ATP7B | 273.179 | -0.56 | 0.20085 | -2.7922 | 0.00524 |  |
| RRAS | 1921.78 | -0.56 | 0.20879 | -2.6894 | 0.00716 |  |
| CARD10 | 2887.2 | -0.56 | 0.17195 | -3.2677 | 0.00108 |  |
| FOS | 1543.23 | -0.56 | 0.2246 | -2.5036 | 0.0123 |  |
| DLG5 | 2367.19 | -0.56 | 0.22563 | -2.4967 | 0.01253 |  |
| KCNN4 | 2635.65 | -0.56 | 0.19931 | -2.8273 | 0.00469 |  |
| LRRFIP1 | 9349.7 | -0.56 | 0.11332 | -4.9846 | 6.21E-07 |  |
| CYFIP1 | 4795.56 | -0.57 | 0.19238 | -2.9429 | 0.00325 |  |
| MUC1 | 9090.51 | -0.57 | 0.21976 | -2.5772 | 0.00996 |  |
| AIM1L | 1372.17 | -0.57 | 0.21112 | -2.6901 | 0.00714 |  |
| SNX33 | 1937.27 | -0.57 | 0.22868 | -2.4842 | 0.01299 |  |
| ENO1 | 28522 | -0.57 | 0.18996 | -3.0017 | 0.00269 |  |
| FGFR4 | 2186.3 | -0.57 | 0.19245 | -2.9637 | 0.00304 |  |
| NMT2 | 541.556 | -0.57 | 0.20939 | -2.7242 | 0.00645 |  |
| CASK | 554.583 | -0.57 | 0.15686 | -3.64 | 0.00027 |  |
| NIN | 815.915 | -0.57 | 0.18685 | -3.0589 | 0.00222 |  |
| LMNA | 22716.5 | -0.57 | 0.15565 | -3.6755 | 0.00024 |  |
| ACSL3 | 3196.9 | -0.57 | 0.13537 | -4.2267 | 2.37E-05 |  |
| FKBP9 | 2900.25 | -0.57 | 0.13233 | -4.3262 | 1.52E-05 |  |
| GNS | 3095.9 | -0.57 | 0.12497 | -4.5848 | 4.54E-06 |  |
| KRT18 | 81821.8 | -0.57 | 0.14647 | -3.916 | 9.00E-05 |  |
| DISP2 | 262.339 | -0.57 | 0.23613 | -2.4321 | 0.01501 |  |
| RARA | 1217.68 | -0.57 | 0.19309 | -2.9775 | 0.00291 |  |
| UBC | 23134.8 | -0.58 | 0.19562 | -2.9422 | 0.00326 |  |
| NCS1 | 569.764 | -0.58 | 0.19113 | -3.0118 | 0.0026 |  |
| TRAF6 | 907.028 | -0.58 | 0.15185 | -3.7923 | 0.00015 |  |
| SLC16A3 | 8231.25 | -0.58 | 0.20853 | -2.7653 | 0.00569 |  |
| ACTB | 171953 | -0.58 | 0.14685 | -3.9375 | 8.23E-05 |  |
| CCDC50 | 3523.33 | -0.58 | 0.16293 | -3.5491 | 0.00039 |  |
| FHL2 | 3873.94 | -0.58 | 0.13932 | -4.1559 | 3.24E-05 |  |
| CDC42EP3 | 770.096 | -0.58 | 0.16823 | -3.4425 | 0.00058 |  |
| ACSL1 | 129.377 | -0.58 | 0.23457 | -2.4695 | 0.01353 |  |
| RND1 | 1407.71 | -0.58 | 0.19914 | -2.9093 | 0.00362 |  |
| DOCK7 | 449.451 | -0.58 | 0.16131 | -3.5977 | 0.00032 |  |
| FAM129B | 13665 | -0.58 | 0.13118 | -4.4342 | 9.24E-06 |  |
| NARF | 1641.71 | -0.58 | 0.17165 | -3.3893 | 0.0007 |  |
| CDC42SE1 | 3856.4 | -0.58 | 0.12833 | -4.5358 | 5.74E-06 |  |
| SQLE | 1989.09 | -0.58 | 0.18248 | -3.1915 | 0.00142 |  |
| SNX25 | 280.615 | -0.58 | 0.19167 | -3.0405 | 0.00236 |  |
| FBXL2 | 269.767 | -0.58 | 0.2294 | -2.5431 | 0.01099 |  |
| BCL7A | 603.711 | -0.58 | 0.24161 | -2.4196 | 0.01554 |  |
| DIP2B | 1671.65 | -0.59 | 0.14718 | -3.9799 | 6.89E-05 |  |
| MVK | 774.558 | -0.59 | 0.17629 | -3.3281 | 0.00087 |  |
| H6PD | 1963.05 | -0.59 | 0.16651 | -3.5334 | 0.00041 |  |
| ACTN4 | 27585 | -0.59 | 0.1124 | -5.2362 | 1.64E-07 |  |
| GPC1 | 1510.13 | -0.59 | 0.15412 | -3.8264 | 0.00013 |  |
| PCDHGA1 | 138.737 | -0.59 | 0.21843 | -2.7004 | 0.00692 |  |
| SCRN1 | 3764.45 | -0.59 | 0.18778 | -3.1412 | 0.00168 |  |
| CNN2 | 12116.4 | -0.59 | 0.1236 | -4.7767 | 1.78E-06 |  |
| SRF | 1226.02 | -0.59 | 0.12974 | -4.5556 | 5.22E-06 |  |
| ANKRD44 | 162.36 | -0.59 | 0.21276 | -2.7842 | 0.00537 |  |
| INPP4B | 453.583 | -0.59 | 0.14549 | -4.0751 | 4.60E-05 |  |
| ARHGAP21 | 4280.78 | -0.59 | 0.17173 | -3.4636 | 0.00053 |  |
| LEPROT | 2279.17 | -0.60 | 0.15917 | -3.7384 | 0.00019 |  |
| CMTM4 | 2295.06 | -0.60 | 0.17174 | -3.4702 | 0.00052 |  |
| DGKA | 2206.92 | -0.60 | 0.22409 | -2.6671 | 0.00765 |  |
| ITGA3 | 14595.9 | -0.60 | 0.18866 | -3.1711 | 0.00152 |  |
| WDR1 | 12204.8 | -0.60 | 0.14254 | -4.1992 | 2.68E-05 |  |
| GRAMD1A | 3122.02 | -0.60 | 0.1181 | -5.0751 | 3.87E-07 |  |
| MAPK7 | 979.717 | -0.60 | 0.15856 | -3.7851 | 0.00015 |  |
| IGSF3 | 1854.2 | -0.60 | 0.22849 | -2.6267 | 0.00862 |  |
| KIFC2 | 1187.78 | -0.60 | 0.21924 | -2.7379 | 0.00618 |  |
| KDM4B | 595.353 | -0.60 | 0.1724 | -3.4904 | 0.00048 |  |
| SPAG9 | 2691.11 | -0.60 | 0.11877 | -5.0683 | 4.01E-07 |  |
| PIEZO1 | 25726.2 | -0.60 | 0.21188 | -2.8444 | 0.00445 |  |
| PLCG1 | 1469.74 | -0.60 | 0.16666 | -3.6175 | 0.0003 |  |
| PCNXL4 | 1455.9 | -0.60 | 0.17595 | -3.4282 | 0.00061 |  |
| C1QTNF6 | 1251.57 | -0.60 | 0.13085 | -4.6113 | 4.00E-06 |  |
| FERMT1 | 442.865 | -0.60 | 0.19119 | -3.1571 | 0.00159 |  |
| DUSP1 | 5842.72 | -0.60 | 0.20209 | -2.9883 | 0.00281 |  |
| ZNF134 | 349.114 | -0.60 | 0.20459 | -2.9527 | 0.00315 |  |
| ITGB1 | 19208.5 | -0.61 | 0.22325 | -2.7103 | 0.00672 |  |
| TPT1-AS1 | 227.512 | -0.61 | 0.22925 | -2.6403 | 0.00828 |  |
| JAK1 | 1911.41 | -0.61 | 0.13713 | -4.419 | 9.91E-06 |  |
| P2RY2 | 496.208 | -0.61 | 0.23781 | -2.5493 | 0.0108 |  |
| AGPAT4 | 261.716 | -0.61 | 0.19044 | -3.1872 | 0.00144 |  |
| VIL1 | 12011.2 | -0.61 | 0.22999 | -2.6402 | 0.00829 |  |
| OPTN | 6024.11 | -0.61 | 0.1691 | -3.5913 | 0.00033 |  |
| JUP | 30848.9 | -0.61 | 0.15209 | -3.9933 | 6.52E-05 |  |
| SUN1 | 3272.6 | -0.61 | 0.14301 | -4.2496 | 2.14E-05 |  |
| DHX32 | 1055.87 | -0.61 | 0.20266 | -3.0049 | 0.00266 |  |
| C20orf194 | 193.954 | -0.61 | 0.18641 | -3.2682 | 0.00108 |  |
| PLEKHO2 | 184.195 | -0.61 | 0.21099 | -2.8893 | 0.00386 |  |
| DDAH2 | 1573.19 | -0.61 | 0.15225 | -4.0079 | 6.13E-05 |  |
| TSPAN9 | 702.547 | -0.61 | 0.13773 | -4.4373 | 9.11E-06 |  |
| IL18 | 1253.24 | -0.61 | 0.16625 | -3.6823 | 0.00023 |  |
| RNF215 | 173.422 | -0.61 | 0.18926 | -3.2376 | 0.00121 |  |
| TES | 6593.98 | -0.62 | 0.12373 | -4.9733 | 6.58E-07 |  |
| B3GNT5 | 888.875 | -0.62 | 0.17707 | -3.4789 | 0.0005 |  |
| NEBL | 3148.88 | -0.62 | 0.25128 | -2.4543 | 0.01411 |  |
| BCAS4 | 121.411 | -0.62 | 0.2367 | -2.6056 | 0.00917 |  |
| RPS6KA4 | 2269.97 | -0.62 | 0.17537 | -3.5217 | 0.00043 |  |
| ALDOA | 38066.1 | -0.62 | 0.14452 | -4.2865 | 1.82E-05 |  |
| BTBD10 | 664.351 | -0.62 | 0.18339 | -3.3901 | 0.0007 |  |
| PDXK | 13026.3 | -0.62 | 0.1768 | -3.518 | 0.00043 |  |
| FNBP1L | 2021.04 | -0.62 | 0.23212 | -2.6807 | 0.00735 |  |
| ANXA1 | 10542.9 | -0.62 | 0.19492 | -3.1931 | 0.00141 |  |
| PAX8 | 460.757 | -0.62 | 0.24671 | -2.5237 | 0.01161 |  |
| WWC1 | 4742.57 | -0.62 | 0.16846 | -3.6961 | 0.00022 |  |
| TRIM29 | 14648.6 | -0.62 | 0.20772 | -3.0059 | 0.00265 |  |
| PTPRK | 1114.94 | -0.62 | 0.16259 | -3.8416 | 0.00012 |  |
| GAPDH | 94549.6 | -0.62 | 0.17591 | -3.5526 | 0.00038 |  |
| PRICKLE4 | 258.988 | -0.63 | 0.19951 | -3.1388 | 0.0017 |  |
| SLC7A6 | 1298.85 | -0.63 | 0.11958 | -5.2447 | 1.57E-07 |  |
| C11orf95 | 322.921 | -0.63 | 0.26021 | -2.4197 | 0.01553 |  |
| NOTCH2 | 1170.31 | -0.63 | 0.15941 | -3.9533 | 7.71E-05 |  |
| CHDH | 732.638 | -0.63 | 0.20303 | -3.1085 | 0.00188 |  |
| EZR | 20721.7 | -0.63 | 0.16169 | -3.9076 | 9.32E-05 |  |
| SNAPC2 | 256.971 | -0.63 | 0.18968 | -3.331 | 0.00087 |  |
| SLC35F2 | 1952.63 | -0.63 | 0.16564 | -3.8148 | 0.00014 |  |
| HERPUD2 | 1255.85 | -0.64 | 0.15339 | -4.141 | 3.46E-05 |  |
| PAWR | 1061.62 | -0.64 | 0.19961 | -3.1831 | 0.00146 |  |
| IDS | 2851.59 | -0.64 | 0.13589 | -4.6772 | 2.91E-06 |  |
| SHANK2 | 738.622 | -0.64 | 0.19956 | -3.1855 | 0.00144 |  |
| RAB11FIP4 | 1683.72 | -0.64 | 0.13023 | -4.8825 | 1.05E-06 |  |
| CD276 | 3394.53 | -0.64 | 0.15254 | -4.173 | 3.01E-05 |  |
| ARHGEF10 | 3411.81 | -0.64 | 0.17285 | -3.6827 | 0.00023 |  |
| PRKCA | 1692.42 | -0.64 | 0.11966 | -5.3334 | 9.64E-08 |  |
| FAM89B | 829.04 | -0.64 | 0.16547 | -3.8654 | 0.00011 |  |
| LMCD1 | 244.241 | -0.64 | 0.22258 | -2.8761 | 0.00403 |  |
| IL32 | 8106.17 | -0.64 | 0.20293 | -3.1669 | 0.00154 |  |
| ZNF185 | 1339.32 | -0.64 | 0.19172 | -3.3559 | 0.00079 |  |
| CTNNA1 | 17833.1 | -0.64 | 0.14841 | -4.3448 | 1.39E-05 |  |
| BMP8B | 169.654 | -0.65 | 0.23705 | -2.7235 | 0.00646 |  |
| ZNF512B | 1524.41 | -0.65 | 0.1339 | -4.8253 | 1.40E-06 |  |
| PCSK4 | 234.968 | -0.65 | 0.18703 | -3.4585 | 0.00054 |  |
| IL22RA1 | 1953.75 | -0.65 | 0.15656 | -4.1331 | 3.58E-05 |  |
| PLBD2 | 1150.85 | -0.65 | 0.12947 | -5.0033 | 5.63E-07 |  |
| DAB2 | 630.668 | -0.65 | 0.26411 | -2.4541 | 0.01413 |  |
| DOCK9 | 866.571 | -0.65 | 0.19303 | -3.3633 | 0.00077 |  |
| SWAP70 | 860.281 | -0.65 | 0.17219 | -3.7743 | 0.00016 |  |
| APLP2 | 12723.6 | -0.65 | 0.21209 | -3.0648 | 0.00218 |  |
| MAMLD1 | 462.456 | -0.65 | 0.14627 | -4.4487 | 8.64E-06 |  |
| PLXNA3 | 1560.32 | -0.65 | 0.16131 | -4.0427 | 5.28E-05 |  |
| KATNAL1 | 584.807 | -0.65 | 0.16223 | -4.023 | 5.74E-05 |  |
| SKIL | 3009.73 | -0.65 | 0.20573 | -3.1748 | 0.0015 |  |
| MGLL | 11494.3 | -0.65 | 0.17084 | -3.8293 | 0.00013 |  |
| CCND1 | 21066.7 | -0.65 | 0.22181 | -2.951 | 0.00317 |  |
| RHBDF2 | 885.234 | -0.66 | 0.27452 | -2.3868 | 0.01699 |  |
| PCYOX1 | 2170.45 | -0.66 | 0.1914 | -3.4244 | 0.00062 |  |
| SUN2 | 1889.43 | -0.66 | 0.12903 | -5.0824 | 3.73E-07 |  |
| CDSN | 563.46 | -0.66 | 0.21445 | -3.0609 | 0.00221 |  |
| IGF1R | 12450.1 | -0.66 | 0.23136 | -2.858 | 0.00426 |  |
| LTBP4 | 4105.82 | -0.66 | 0.14623 | -4.5373 | 5.70E-06 |  |
| TMEM159 | 495.149 | -0.66 | 0.20421 | -3.2507 | 0.00115 |  |
| HTRA1 | 352.069 | -0.66 | 0.26699 | -2.4888 | 0.01282 |  |
| TLE1 | 843.796 | -0.67 | 0.19243 | -3.4659 | 0.00053 |  |
| CTTNBP2NL | 884.25 | -0.67 | 0.18235 | -3.66 | 0.00025 |  |
| CEP170 | 727.356 | -0.67 | 0.20385 | -3.2805 | 0.00104 |  |
| ETHE1 | 1259.08 | -0.67 | 0.16828 | -3.9816 | 6.85E-05 |  |
| CYTH1 | 2736.7 | -0.67 | 0.15591 | -4.2989 | 1.72E-05 |  |
| TSHZ1 | 609.58 | -0.67 | 0.1393 | -4.8125 | 1.49E-06 |  |
| DUSP14 | 647.73 | -0.67 | 0.18654 | -3.5959 | 0.00032 |  |
| LINC01322 | 154.161 | -0.67 | 0.27112 | -2.4742 | 0.01336 |  |
| KREMEN1 | 409.935 | -0.67 | 0.19297 | -3.4819 | 0.0005 |  |
| C14orf132 | 147.04 | -0.67 | 0.23625 | -2.8519 | 0.00435 |  |
| MED15 | 3042.41 | -0.67 | 0.13452 | -5.013 | 5.36E-07 |  |
| AGRN | 12240.6 | -0.67 | 0.15918 | -4.2394 | 2.24E-05 |  |
| DYNC1H1 | 17557.8 | -0.67 | 0.19415 | -3.4759 | 0.00051 |  |
| MICA | 835.431 | -0.68 | 0.14315 | -4.7232 | 2.32E-06 |  |
| RIOK3 | 1703.1 | -0.68 | 0.10863 | -6.2255 | 4.80E-10 |  |
| GAS5 | 3415.18 | -0.68 | 0.1937 | -3.4973 | 0.00047 |  |
| PLCE1 | 119.789 | -0.68 | 0.23694 | -2.8592 | 0.00425 |  |
| HMGCR | 1833.55 | -0.68 | 0.16178 | -4.1957 | 2.72E-05 |  |
| KIF1C | 5159.54 | -0.68 | 0.13553 | -5.0104 | 5.43E-07 |  |
| COTL1 | 886.242 | -0.68 | 0.19464 | -3.4913 | 0.00048 |  |
| PACS1 | 4335.63 | -0.68 | 0.16712 | -4.074 | 4.62E-05 |  |
| STEAP3 | 2074.25 | -0.68 | 0.18749 | -3.6317 | 0.00028 |  |
| ACAA2 | 2580.16 | -0.68 | 0.22091 | -3.0855 | 0.00203 |  |
| RAB30 | 245.8 | -0.68 | 0.20891 | -3.2716 | 0.00107 |  |
| CTNNBIP1 | 355.321 | -0.68 | 0.15763 | -4.3399 | 1.43E-05 |  |
| DSG2 | 6068.58 | -0.68 | 0.13796 | -4.9595 | 7.07E-07 |  |
| GADD45B | 898.539 | -0.68 | 0.26395 | -2.5946 | 0.00947 |  |
| DUSP6 | 519.694 | -0.68 | 0.26029 | -2.6313 | 0.00851 |  |
| MIR614 | 305.14 | -0.68 | 0.21658 | -3.1625 | 0.00156 |  |
| MKL1 | 1782.27 | -0.69 | 0.17674 | -3.8775 | 0.00011 |  |
| RND3 | 1716.15 | -0.69 | 0.17599 | -3.8946 | 9.84E-05 |  |
| FLJ20021 | 127.532 | -0.69 | 0.27043 | -2.5352 | 0.01124 |  |
| ZFHX3 | 3988.24 | -0.69 | 0.14366 | -4.774 | 1.81E-06 |  |
| FXYD5 | 2771.22 | -0.69 | 0.19627 | -3.4947 | 0.00047 |  |
| TNFRSF12A | 4343.48 | -0.69 | 0.21572 | -3.1839 | 0.00145 |  |
| PINK1 | 351.142 | -0.69 | 0.19553 | -3.5142 | 0.00044 |  |
| PAM | 1611.96 | -0.69 | 0.22177 | -3.0992 | 0.00194 |  |
| GPI | 12807.7 | -0.69 | 0.19057 | -3.6072 | 0.00031 |  |
| CAPRIN2 | 556.265 | -0.69 | 0.15728 | -4.371 | 1.24E-05 |  |
| ZNF71 | 356.401 | -0.69 | 0.23363 | -2.944 | 0.00324 |  |
| PHYHIP | 392.786 | -0.69 | 0.18174 | -3.791 | 0.00015 |  |
| LIMK2 | 946.287 | -0.69 | 0.21645 | -3.1856 | 0.00144 |  |
| ILK | 3153.85 | -0.69 | 0.169 | -4.0832 | 4.44E-05 |  |
| SLC12A4 | 2156.7 | -0.69 | 0.16656 | -4.1436 | 3.42E-05 |  |
| PARVA | 2064.76 | -0.69 | 0.14138 | -4.8881 | 1.02E-06 |  |
| PTPRE | 324.829 | -0.69 | 0.15111 | -4.5839 | 4.56E-06 |  |
| LRRC3 | 137.486 | -0.69 | 0.2606 | -2.6583 | 0.00785 |  |
| ABHD11-AS1 | 556.892 | -0.69 | 0.23063 | -3.0064 | 0.00264 |  |
| AHDC1 | 850.951 | -0.69 | 0.23595 | -2.94 | 0.00328 |  |
| TAGLN2 | 32345.7 | -0.69 | 0.16667 | -4.1627 | 3.14E-05 |  |
| IL13RA1 | 3823.54 | -0.69 | 0.20318 | -3.417 | 0.00063 |  |
| CATSPER1 | 166.429 | -0.69 | 0.28179 | -2.4649 | 0.01371 |  |
| RTN4R | 245.796 | -0.69 | 0.25282 | -2.7474 | 0.00601 |  |
| YPEL1 | 207.983 | -0.69 | 0.23022 | -3.0181 | 0.00254 |  |
| ATP8B2 | 131.263 | -0.69 | 0.26396 | -2.6327 | 0.00847 |  |
| CLSTN1 | 3635.52 | -0.70 | 0.14125 | -4.9229 | 8.53E-07 |  |
| HABP4 | 213.156 | -0.70 | 0.24557 | -2.8386 | 0.00453 |  |
| APBA2 | 195.167 | -0.70 | 0.24679 | -2.8248 | 0.00473 |  |
| MAP4 | 5168.95 | -0.70 | 0.15156 | -4.6002 | 4.22E-06 |  |
| AK4 | 518.894 | -0.70 | 0.17858 | -3.913 | 9.12E-05 |  |
| RGS10 | 250.282 | -0.70 | 0.27198 | -2.5721 | 0.01011 |  |
| YPEL2 | 480.05 | -0.70 | 0.23642 | -2.959 | 0.00309 |  |
| NCOR2 | 15726.9 | -0.70 | 0.20983 | -3.3342 | 0.00086 |  |
| AMOT | 165.884 | -0.70 | 0.25532 | -2.7403 | 0.00614 |  |
| COL18A1 | 4135.71 | -0.70 | 0.20809 | -3.3641 | 0.00077 |  |
| NFATC2 | 604.506 | -0.70 | 0.2201 | -3.1815 | 0.00147 |  |
| SPECC1 | 1366.63 | -0.70 | 0.1462 | -4.7942 | 1.63E-06 |  |
| DSP | 24409 | -0.70 | 0.16223 | -4.3225 | 1.54E-05 |  |
| RUNX1 | 2230.15 | -0.70 | 0.10992 | -6.3879 | 1.68E-10 |  |
| SMAD7 | 854.325 | -0.71 | 0.21202 | -3.3264 | 0.00088 |  |
| AFAP1 | 1541.69 | -0.71 | 0.21659 | -3.2577 | 0.00112 |  |
| SOX9 | 1996.94 | -0.71 | 0.16346 | -4.3167 | 1.58E-05 |  |
| PACSIN3 | 1547.88 | -0.71 | 0.23433 | -3.0144 | 0.00257 |  |
| UNC13D | 7607.79 | -0.71 | 0.23028 | -3.0729 | 0.00212 |  |
| B3GLCT | 373.464 | -0.71 | 0.19585 | -3.6193 | 0.0003 |  |
| KLHL25 | 299.219 | -0.71 | 0.19941 | -3.5597 | 0.00037 |  |
| PLEKHA8P1 | 126.14 | -0.71 | 0.23764 | -2.9963 | 0.00273 |  |
| TNFAIP3 | 296.955 | -0.71 | 0.2351 | -3.0341 | 0.00241 |  |
| MARK4 | 727.154 | -0.71 | 0.18611 | -3.8378 | 0.00012 |  |
| CORO1C | 5865.68 | -0.72 | 0.16002 | -4.4757 | 7.62E-06 |  |
| ZNF778 | 163.106 | -0.72 | 0.24352 | -2.9466 | 0.00321 |  |
| LDHA | 31642.6 | -0.72 | 0.12677 | -5.6698 | 1.43E-08 |  |
| KDM7A | 456.572 | -0.72 | 0.156 | -4.6133 | 3.96E-06 |  |
| PTPN14 | 2841.95 | -0.72 | 0.19291 | -3.7403 | 0.00018 |  |
| SFXN3 | 5134.19 | -0.72 | 0.20552 | -3.5136 | 0.00044 |  |
| STARD13 | 304.656 | -0.72 | 0.22797 | -3.1703 | 0.00152 |  |
| FASN | 12904.6 | -0.72 | 0.17422 | -4.1502 | 3.32E-05 |  |
| FAM46B | 238.631 | -0.72 | 0.2066 | -3.5006 | 0.00046 |  |
| KLF12 | 238.946 | -0.72 | 0.24248 | -2.9838 | 0.00285 |  |
| LIMS1 | 1134.82 | -0.73 | 0.17557 | -4.1379 | 3.51E-05 |  |
| PCDHGB2 | 323.627 | -0.73 | 0.24344 | -2.9847 | 0.00284 |  |
| ZNF496 | 596.9 | -0.73 | 0.19113 | -3.8104 | 0.00014 |  |
| LSS | 3181.78 | -0.73 | 0.15385 | -4.7351 | 2.19E-06 |  |
| EVA1B | 289.548 | -0.73 | 0.21517 | -3.3901 | 0.0007 |  |
| TRIP10 | 2094.67 | -0.73 | 0.16848 | -4.3447 | 1.39E-05 |  |
| LIMCH1 | 3633.58 | -0.73 | 0.24424 | -3.0011 | 0.00269 |  |
| KDM6B | 2148.65 | -0.73 | 0.18099 | -4.051 | 5.10E-05 |  |
| AMOTL2 | 8287.23 | -0.73 | 0.22136 | -3.313 | 0.00092 |  |
| PSORS1C3 | 87.5117 | -0.74 | 0.30406 | -2.4213 | 0.01546 |  |
| PKM | 40993.7 | -0.74 | 0.15323 | -4.8304 | 1.36E-06 |  |
| CRIP2 | 808.104 | -0.74 | 0.30358 | -2.4416 | 0.01462 |  |
| CD40 | 223.272 | -0.74 | 0.23031 | -3.2219 | 0.00127 |  |
| FKBP1B | 128.521 | -0.74 | 0.24812 | -2.9916 | 0.00278 |  |
| PXDC1 | 1364.69 | -0.74 | 0.18503 | -4.0142 | 5.96E-05 |  |
| SLC9A2 | 584.904 | -0.74 | 0.21233 | -3.5034 | 0.00046 |  |
| TUBB2A | 488.064 | -0.74 | 0.2148 | -3.468 | 0.00052 |  |
| ID1 | 1272.91 | -0.74 | 0.14115 | -5.2775 | 1.31E-07 |  |
| GDPD5 | 280.386 | -0.75 | 0.17757 | -4.1998 | 2.67E-05 |  |
| FAM109A | 1961.82 | -0.75 | 0.25604 | -2.9169 | 0.00353 |  |
| SAT1 | 3456.98 | -0.75 | 0.18652 | -4.0091 | 6.09E-05 |  |
| KANK2 | 771.809 | -0.75 | 0.20637 | -3.6311 | 0.00028 |  |
| PVRL3 | 471.099 | -0.75 | 0.2407 | -3.1152 | 0.00184 |  |
| LATS2 | 3088.39 | -0.75 | 0.19319 | -3.8842 | 0.0001 |  |
| LAT2 | 224.851 | -0.75 | 0.29121 | -2.5768 | 0.00997 |  |
| ODC1 | 4615.83 | -0.75 | 0.16551 | -4.5363 | 5.72E-06 |  |
| PRICKLE1 | 132.98 | -0.75 | 0.30141 | -2.4945 | 0.01261 |  |
| TRIM46 | 68.0988 | -0.75 | 0.28813 | -2.6135 | 0.00896 |  |
| SGTB | 267.205 | -0.75 | 0.26725 | -2.8182 | 0.00483 |  |
| RHBDF1 | 1344.91 | -0.75 | 0.17662 | -4.2646 | 2.00E-05 |  |
| HRH1 | 415.139 | -0.75 | 0.20049 | -3.7615 | 0.00017 |  |
| PLOD1 | 1180.11 | -0.76 | 0.19166 | -3.9405 | 8.13E-05 |  |
| NBPF18P | 160.005 | -0.76 | 0.22298 | -3.3959 | 0.00068 |  |
| RHOD | 4653.24 | -0.76 | 0.2046 | -3.7041 | 0.00021 |  |
| PMP22 | 310.416 | -0.76 | 0.25476 | -2.9799 | 0.00288 |  |
| ACAP1 | 168.386 | -0.76 | 0.19731 | -3.8523 | 0.00012 |  |
| DHCR7 | 3183.37 | -0.76 | 0.17787 | -4.2819 | 1.85E-05 |  |
| MYO5A | 164.817 | -0.76 | 0.25173 | -3.033 | 0.00242 |  |
| SYT15 | 265.912 | -0.76 | 0.26355 | -2.8972 | 0.00376 |  |
| GDF11 | 79.6706 | -0.76 | 0.25937 | -2.9479 | 0.0032 |  |
| LINC00842 | 1339.08 | -0.77 | 0.21194 | -3.6101 | 0.00031 |  |
| PGK1 | 10392.4 | -0.77 | 0.17476 | -4.3842 | 1.16E-05 |  |
| TM4SF1 | 13769.4 | -0.77 | 0.13851 | -5.5376 | 3.07E-08 |  |
| GLDN | 55.2298 | -0.77 | 0.30595 | -2.5081 | 0.01214 |  |
| CLCN4 | 768.589 | -0.77 | 0.27384 | -2.8036 | 0.00505 |  |
| DUSP10 | 115.563 | -0.77 | 0.22627 | -3.3988 | 0.00068 |  |
| FGD6 | 2132.73 | -0.77 | 0.17561 | -4.3823 | 1.17E-05 |  |
| ECE1 | 6122.68 | -0.77 | 0.20043 | -3.8429 | 0.00012 |  |
| ANKLE2 | 4892.33 | -0.77 | 0.12993 | -5.9321 | 2.99E-09 |  |
| KRT80 | 6937.79 | -0.77 | 0.24689 | -3.1236 | 0.00179 |  |
| RP1 | 70.1929 | -0.77 | 0.29853 | -2.5836 | 0.00978 |  |
| IRS1 | 1025.26 | -0.77 | 0.21037 | -3.6787 | 0.00023 |  |
| NMNAT2 | 329.722 | -0.78 | 0.2547 | -3.0449 | 0.00233 |  |
| PLEC | 56915 | -0.78 | 0.12281 | -6.3226 | 2.57E-10 |  |
| SOCS3 | 289.045 | -0.78 | 0.23834 | -3.2699 | 0.00108 |  |
| ITPR3 | 13173 | -0.78 | 0.18683 | -4.1735 | 3.00E-05 |  |
| DBN1 | 5860.52 | -0.78 | 0.14117 | -5.5277 | 3.25E-08 |  |
| DLX4 | 70.627 | -0.78 | 0.304 | -2.5679 | 0.01023 |  |
| OSBPL10 | 824.13 | -0.78 | 0.21606 | -3.6242 | 0.00029 |  |
| CACNB3 | 827.891 | -0.78 | 0.21922 | -3.5724 | 0.00035 |  |
| FAXDC2 | 7848 | -0.78 | 0.18179 | -4.3103 | 1.63E-05 |  |
| BTBD19 | 166.722 | -0.78 | 0.23944 | -3.275 | 0.00106 |  |
| AHNAK | 45380 | -0.78 | 0.09129 | -8.592 | 8.55E-18 |  |
| MB21D2 | 392.757 | -0.79 | 0.28731 | -2.733 | 0.00628 |  |
| CDCP1 | 1906.67 | -0.79 | 0.17406 | -4.5121 | 6.42E-06 |  |
| C16orf74 | 427.819 | -0.79 | 0.20825 | -3.7739 | 0.00016 |  |
| CLTB | 7135.97 | -0.79 | 0.24816 | -3.1691 | 0.00153 |  |
| KLHL5 | 1133.16 | -0.79 | 0.18336 | -4.2906 | 1.78E-05 |  |
| DGCR11 | 73.8917 | -0.79 | 0.2874 | -2.7391 | 0.00616 |  |
| LOC100132077 | 179.916 | -0.79 | 0.2165 | -3.6405 | 0.00027 |  |
| LDLR | 2583.63 | -0.79 | 0.17224 | -4.5809 | 4.63E-06 |  |
| PGBD1 | 169.815 | -0.79 | 0.22614 | -3.4954 | 0.00047 |  |
| NAB2 | 485.419 | -0.79 | 0.28161 | -2.8076 | 0.00499 |  |
| DYSF | 987.735 | -0.79 | 0.32819 | -2.4128 | 0.01583 |  |
| MSC-AS1 | 40.1061 | -0.79 | 0.321 | -2.4672 | 0.01362 |  |
| PSORS1C1 | 329.782 | -0.79 | 0.23693 | -3.3433 | 0.00083 |  |
| MYADM | 13154.9 | -0.79 | 0.15236 | -5.2092 | 1.90E-07 |  |
| LIX1L | 383.878 | -0.79 | 0.25567 | -3.1054 | 0.0019 |  |
| CBLB | 423.482 | -0.79 | 0.16832 | -4.7208 | 2.35E-06 |  |
| HIVEP1 | 814.26 | -0.80 | 0.17157 | -4.6364 | 3.55E-06 |  |
| TSNARE1 | 403.344 | -0.80 | 0.16107 | -4.9405 | 7.79E-07 |  |
| RAB11FIP5 | 1346.77 | -0.80 | 0.19762 | -4.03 | 5.58E-05 |  |
| HIVEP2 | 531.299 | -0.80 | 0.1737 | -4.5988 | 4.25E-06 |  |
| SOWAHC | 337.119 | -0.80 | 0.26804 | -2.9806 | 0.00288 |  |
| TSC22D2 | 646.797 | -0.80 | 0.13883 | -5.7603 | 8.40E-09 |  |
| ZDHHC3 | 1388.62 | -0.80 | 0.14398 | -5.5569 | 2.75E-08 |  |
| ATXN1 | 1277.83 | -0.80 | 0.17804 | -4.506 | 6.61E-06 |  |
| DMBX1 | 50.7646 | -0.80 | 0.31699 | -2.5312 | 0.01137 |  |
| ATF3 | 851.424 | -0.80 | 0.23553 | -3.41 | 0.00065 |  |
| FADS3 | 358.755 | -0.80 | 0.18496 | -4.3486 | 1.37E-05 |  |
| SCEL | 4039.98 | -0.81 | 0.17276 | -4.6788 | 2.89E-06 |  |
| DCDC5 | 198.006 | -0.81 | 0.24372 | -3.3175 | 0.00091 |  |
| ADRA2C | 157.675 | -0.81 | 0.33532 | -2.4141 | 0.01577 |  |
| KLHL26 | 184.356 | -0.81 | 0.27504 | -2.9522 | 0.00316 |  |
| TMOD2 | 118.339 | -0.81 | 0.28369 | -2.8622 | 0.00421 |  |
| SHROOM2 | 294.185 | -0.81 | 0.31586 | -2.5709 | 0.01014 |  |
| PFKFB3 | 1682.52 | -0.81 | 0.13331 | -6.0995 | 1.06E-09 |  |
| SYNE3 | 49.9334 | -0.81 | 0.31404 | -2.5898 | 0.0096 |  |
| VAT1 | 9848.92 | -0.81 | 0.17788 | -4.5784 | 4.68E-06 |  |
| ULBP2 | 39.1023 | -0.82 | 0.34323 | -2.3793 | 0.01734 |  |
| GSN-AS1 | 64.1094 | -0.82 | 0.29323 | -2.7861 | 0.00533 |  |
| TINAGL1 | 1487.05 | -0.82 | 0.12746 | -6.4133 | 1.42E-10 |  |
| PMAIP1 | 224.8 | -0.82 | 0.22237 | -3.6768 | 0.00024 |  |
| MMP14 | 116.018 | -0.82 | 0.34115 | -2.3982 | 0.01648 |  |
| MTHFD1L | 963.188 | -0.82 | 0.15442 | -5.3017 | 1.15E-07 |  |
| MVD | 2092.17 | -0.82 | 0.15551 | -5.2658 | 1.40E-07 |  |
| R3HDML | 50.3254 | -0.82 | 0.33746 | -2.429 | 0.01514 |  |
| FARP1 | 1799.83 | -0.82 | 0.19405 | -4.231 | 2.33E-05 |  |
| ESYT2 | 4962.13 | -0.82 | 0.13419 | -6.1202 | 9.35E-10 |  |
| LOC654342 | 274.338 | -0.82 | 0.22671 | -3.6247 | 0.00029 |  |
| TMEM150C | 92.11 | -0.82 | 0.2697 | -3.0521 | 0.00227 |  |
| PTGES | 2474.36 | -0.82 | 0.29612 | -2.78 | 0.00544 |  |
| PROS1 | 1712.68 | -0.82 | 0.13004 | -6.3309 | 2.44E-10 |  |
| MXI1 | 503.227 | -0.83 | 0.23873 | -3.4602 | 0.00054 |  |
| C19orf71 | 242.743 | -0.83 | 0.28853 | -2.8652 | 0.00417 |  |
| EPB41L2 | 856.713 | -0.83 | 0.18726 | -4.4174 | 9.99E-06 |  |
| TMEM43 | 1464.79 | -0.83 | 0.20214 | -4.1151 | 3.87E-05 |  |
| HIF1A | 1246.39 | -0.83 | 0.16629 | -5.0136 | 5.34E-07 |  |
| VAV1 | 87.0692 | -0.83 | 0.25843 | -3.2284 | 0.00124 |  |
| BMPR2 | 3046.14 | -0.83 | 0.22192 | -3.7622 | 0.00017 |  |
| NUMBL | 1405.64 | -0.83 | 0.11321 | -7.3748 | 1.65E-13 |  |
| GAA | 1969.8 | -0.84 | 0.14753 | -5.662 | 1.50E-08 |  |
| FLNB | 25651.6 | -0.84 | 0.14758 | -5.6644 | 1.48E-08 |  |
| PCDHGA9 | 69.2226 | -0.84 | 0.28692 | -2.9163 | 0.00354 |  |
| SDC3 | 1646.88 | -0.84 | 0.25838 | -3.2429 | 0.00118 |  |
| HDAC7 | 2128.58 | -0.84 | 0.18326 | -4.5737 | 4.79E-06 |  |
| CTPS1 | 940.865 | -0.84 | 0.18593 | -4.5103 | 6.47E-06 |  |
| CNTNAP2 | 469.255 | -0.84 | 0.2383 | -3.5229 | 0.00043 |  |
| CSPG4 | 93.0877 | -0.84 | 0.27481 | -3.0555 | 0.00225 |  |
| GJB4 | 418.914 | -0.84 | 0.28583 | -2.9407 | 0.00328 |  |
| IGFBP6 | 176.116 | -0.84 | 0.32943 | -2.5549 | 0.01062 |  |
| ONECUT2 | 429.428 | -0.84 | 0.21504 | -3.9188 | 8.90E-05 |  |
| ITGBL1 | 1206.51 | -0.84 | 0.28733 | -2.933 | 0.00336 |  |
| MARCH2        . | 355.76 | -0.84 | 0.16817 | -5.023 | 5.09E-07 |  |
| BICD2 | 1108.43 | -0.85 | 0.25469 | -3.3183 | 0.00091 |  |
| RASSF2 | 161.628 | -0.85 | 0.25208 | -3.3599 | 0.00078 |  |
| LOC284581 | 281.824 | -0.85 | 0.32046 | -2.6486 | 0.00808 |  |
| TBC1D16 | 996.315 | -0.85 | 0.23839 | -3.5657 | 0.00036 |  |
| TMC7 | 452.333 | -0.85 | 0.24608 | -3.4601 | 0.00054 |  |
| BAIAP3 | 226.859 | -0.85 | 0.33791 | -2.522 | 0.01167 |  |
| MYO15A | 43.7129 | -0.85 | 0.3303 | -2.5847 | 0.00975 |  |
| DLGAP4 | 2811.44 | -0.86 | 0.1693 | -5.0577 | 4.24E-07 |  |
| RGS20 | 127.993 | -0.86 | 0.35156 | -2.4408 | 0.01465 |  |
| PCDHGC5 | 34.4959 | -0.86 | 0.33634 | -2.5529 | 0.01068 |  |
| TIMP4 | 31.6579 | -0.86 | 0.34369 | -2.5053 | 0.01223 |  |
| ACSS2 | 1134.09 | -0.86 | 0.15392 | -5.5974 | 2.18E-08 |  |
| BCAR3 | 1094.18 | -0.86 | 0.24397 | -3.5377 | 0.0004 |  |
| QSOX1 | 2299.76 | -0.87 | 0.14168 | -6.1098 | 9.97E-10 |  |
| IL1RAP | 120.434 | -0.87 | 0.26598 | -3.2552 | 0.00113 |  |
| ANXA3 | 5683.52 | -0.87 | 0.18409 | -4.7048 | 2.54E-06 |  |
| WWC3 | 1874.79 | -0.87 | 0.2361 | -3.6743 | 0.00024 |  |
| HMOX1 | 290.442 | -0.87 | 0.30818 | -2.8262 | 0.00471 |  |
| FBN2 | 24.7555 | -0.87 | 0.36229 | -2.4057 | 0.01614 |  |
| FOLR1 | 241.735 | -0.87 | 0.26115 | -3.342 | 0.00083 |  |
| CCDC102A | 239.79 | -0.87 | 0.27299 | -3.2013 | 0.00137 |  |
| NCF2 | 1371.73 | -0.88 | 0.20569 | -4.2669 | 1.98E-05 |  |
| CPSF1 | 4223.3 | -0.88 | 0.19976 | -4.3966 | 1.10E-05 |  |
| TARSL2 | 436.536 | -0.88 | 0.20947 | -4.1954 | 2.72E-05 |  |
| USP2 | 24.1085 | -0.88 | 0.36061 | -2.438 | 0.01477 |  |
| GLI2 | 1380.47 | -0.88 | 0.27721 | -3.1797 | 0.00147 |  |
| LRP1 | 2896.26 | -0.88 | 0.12536 | -7.0435 | 1.87E-12 |  |
| PDK1 | 787.795 | -0.88 | 0.1696 | -5.2065 | 1.92E-07 |  |
| ACTG1 | 210126 | -0.88 | 0.1638 | -5.3911 | 7.00E-08 |  |
| CD151 | 19905.7 | -0.88 | 0.18457 | -4.7893 | 1.67E-06 |  |
| IGFBP4 | 19882 | -0.89 | 0.19768 | -4.487 | 7.22E-06 |  |
| PCED1B | 3213.45 | -0.89 | 0.23047 | -3.8584 | 0.00011 |  |
| GPR155 | 274.864 | -0.89 | 0.28427 | -3.1297 | 0.00175 |  |
| NRXN3 | 143.228 | -0.89 | 0.35528 | -2.5111 | 0.01203 |  |
| ITPRIP | 98.9498 | -0.90 | 0.30951 | -2.8962 | 0.00378 |  |
| TIMP2 | 12124.5 | -0.90 | 0.28186 | -3.1825 | 0.00146 |  |
| P4HA1 | 1694.84 | -0.90 | 0.2461 | -3.6506 | 0.00026 |  |
| SLC38A2 | 3330.39 | -0.90 | 0.17867 | -5.0287 | 4.94E-07 |  |
| SLC6A20 | 80.577 | -0.90 | 0.36145 | -2.486 | 0.01292 |  |
| MEX3B | 263.988 | -0.90 | 0.24052 | -3.7419 | 0.00018 |  |
| S100A2 | 294.172 | -0.90 | 0.23522 | -3.8334 | 0.00013 |  |
| MLC1 | 80.5567 | -0.90 | 0.35794 | -2.5206 | 0.01172 |  |
| INSIG1 | 958.76 | -0.90 | 0.17833 | -5.0595 | 4.20E-07 |  |
| MACF1 | 3192.7 | -0.90 | 0.1771 | -5.0978 | 3.44E-07 |  |
| SEMA6C | 146.583 | -0.90 | 0.23325 | -3.872 | 0.00011 |  |
| ENAH | 1626.82 | -0.90 | 0.16629 | -5.4403 | 5.32E-08 |  |
| LOC100506548 | 579.625 | -0.91 | 0.19244 | -4.7114 | 2.46E-06 |  |
| GABRA5 | 33.5295 | -0.91 | 0.35889 | -2.5301 | 0.0114 |  |
| LOC284454 | 625.57 | -0.91 | 0.21939 | -4.1477 | 3.36E-05 |  |
| KLC1 | 2616.58 | -0.91 | 0.11692 | -7.7873 | 6.84E-15 |  |
| FAM160A1 | 84.2027 | -0.91 | 0.2552 | -3.5688 | 0.00036 |  |
| MYCN | 78.3346 | -0.91 | 0.37884 | -2.4055 | 0.01615 |  |
| HSPG2 | 9031.12 | -0.91 | 0.21156 | -4.3081 | 1.65E-05 |  |
| GJB3 | 2098.69 | -0.91 | 0.21308 | -4.2822 | 1.85E-05 |  |
| CSRP1 | 8999.78 | -0.91 | 0.1103 | -8.278 | 1.25E-16 |  |
| P4HA2 | 1023.84 | -0.91 | 0.1732 | -5.2751 | 1.33E-07 |  |
| PCSK6 | 1864.98 | -0.91 | 0.23797 | -3.8442 | 0.00012 |  |
| KLF4 | 3798.09 | -0.92 | 0.15592 | -5.8684 | 4.40E-09 |  |
| GJC2 | 263.93 | -0.92 | 0.37937 | -2.4162 | 0.01568 |  |
| LHX1 | 52.3762 | -0.92 | 0.36882 | -2.4871 | 0.01288 |  |
| EVL | 321.013 | -0.92 | 0.17404 | -5.2779 | 1.31E-07 |  |
| ZDHHC8 | 461.145 | -0.92 | 0.25572 | -3.5949 | 0.00032 |  |
| KANK4 | 124.778 | -0.92 | 0.35921 | -2.5609 | 0.01044 |  |
| ADCY7 | 2061.54 | -0.92 | 0.24911 | -3.694 | 0.00022 |  |
| STC2 | 2593.7 | -0.92 | 0.17522 | -5.2528 | 1.50E-07 |  |
| GCSAM | 88.2086 | -0.92 | 0.25273 | -3.6462 | 0.00027 |  |
| LUZP1 | 937.482 | -0.92 | 0.12852 | -7.1716 | 7.41E-13 |  |
| ADGRG1 | 13674.1 | -0.92 | 0.17543 | -5.255 | 1.48E-07 |  |
| DAPK3 | 1363.76 | -0.92 | 0.17533 | -5.2586 | 1.45E-07 |  |
| KIAA0754 | 254.284 | -0.92 | 0.2176 | -4.2432 | 2.20E-05 |  |
| DACT1 | 12.1669 | -0.93 | 0.38373 | -2.4106 | 0.01593 |  |
| JUNB | 3351.4 | -0.93 | 0.27617 | -3.3525 | 0.0008 |  |
| IFFO2 | 283.209 | -0.93 | 0.21078 | -4.3943 | 1.11E-05 |  |
| UBL3 | 1449.38 | -0.93 | 0.20839 | -4.4564 | 8.34E-06 |  |
| RECK | 62.5023 | -0.93 | 0.30983 | -3.001 | 0.00269 |  |
| KISS1 | 31.4438 | -0.93 | 0.37184 | -2.5032 | 0.01231 |  |
| ZNF699 | 33.2253 | -0.93 | 0.34496 | -2.7027 | 0.00688 |  |
| MSN | 7143.13 | -0.93 | 0.16238 | -5.7457 | 9.15E-09 |  |
| LIFR | 37.7867 | -0.93 | 0.35436 | -2.6336 | 0.00845 |  |
| TRIO | 9033.07 | -0.93 | 0.20304 | -4.6037 | 4.15E-06 |  |
| ACTN1 | 4054.48 | -0.94 | 0.19729 | -4.7403 | 2.13E-06 |  |
| MATK | 12.3124 | -0.94 | 0.3807 | -2.4641 | 0.01373 |  |
| SERTAD4 | 212.295 | -0.94 | 0.26939 | -3.4861 | 0.00049 |  |
| MACROD2 | 18.2453 | -0.94 | 0.38271 | -2.4572 | 0.014 |  |
| SH3KBP1 | 1895.56 | -0.94 | 0.13131 | -7.1923 | 6.37E-13 |  |
| AEBP1 | 18.7701 | -0.94 | 0.37075 | -2.5475 | 0.01085 |  |
| LSAMP | 126.253 | -0.95 | 0.27836 | -3.4039 | 0.00066 |  |
| FNDC3B | 1730.43 | -0.95 | 0.19584 | -4.8404 | 1.30E-06 |  |
| SYNPO | 8697.91 | -0.95 | 0.21347 | -4.4409 | 8.96E-06 |  |
| LAMC1 | 5101.64 | -0.95 | 0.1534 | -6.1832 | 6.28E-10 |  |
| PEG10 | 228.967 | -0.95 | 0.22637 | -4.1952 | 2.73E-05 |  |
| ARHGEF18 | 2965.13 | -0.95 | 0.23126 | -4.1106 | 3.95E-05 |  |
| VCL | 5883.19 | -0.95 | 0.19164 | -4.964 | 6.90E-07 |  |
| FGF1 | 413.41 | -0.95 | 0.22861 | -4.1679 | 3.07E-05 |  |
| FAM20C | 1191.05 | -0.95 | 0.3869 | -2.4642 | 0.01373 |  |
| SP6 | 137.292 | -0.96 | 0.32775 | -2.9171 | 0.00353 |  |
| ITGA6 | 6595.35 | -0.96 | 0.22256 | -4.2988 | 1.72E-05 |  |
| KIF26B | 48.0012 | -0.96 | 0.38864 | -2.4665 | 0.01365 |  |
| STARD4 | 900.905 | -0.96 | 0.1891 | -5.0736 | 3.90E-07 |  |
| LOX | 25.5195 | -0.96 | 0.37091 | -2.5889 | 0.00963 |  |
| LOC389602 | 159.41 | -0.96 | 0.26569 | -3.6141 | 0.0003 |  |
| CADM4 | 398.695 | -0.96 | 0.22403 | -4.2944 | 1.75E-05 |  |
| LOC100506990 | 832.328 | -0.96 | 0.19359 | -4.972 | 6.63E-07 |  |
| IL31RA | 68.445 | -0.96 | 0.32727 | -2.9473 | 0.00321 |  |
| PIM1 | 1967.23 | -0.97 | 0.15256 | -6.3256 | 2.52E-10 |  |
| TUBB6 | 1787.97 | -0.97 | 0.17501 | -5.5195 | 3.40E-08 |  |
| MAFK | 7814.28 | -0.97 | 0.1804 | -5.3549 | 8.56E-08 |  |
| COL4A3 | 1991.04 | -0.97 | 0.14066 | -6.8692 | 6.46E-12 |  |
| PID1 | 844.954 | -0.97 | 0.27017 | -3.578 | 0.00035 |  |
| ANKRD29 | 65.4231 | -0.97 | 0.36595 | -2.647 | 0.00812 |  |
| DNAJB2 | 3623.49 | -0.97 | 0.19768 | -4.9041 | 9.39E-07 |  |
| BICC1 | 706.215 | -0.97 | 0.32181 | -3.0154 | 0.00257 |  |
| TIAM1 | 48.6204 | -0.97 | 0.31703 | -3.0655 | 0.00217 |  |
| PLCG2 | 84.8958 | -0.97 | 0.2527 | -3.8504 | 0.00012 |  |
| LARP6 | 339.66 | -0.97 | 0.2418 | -4.0242 | 5.72E-05 |  |
| TNFAIP1 | 3687.75 | -0.97 | 0.14436 | -6.7439 | 1.54E-11 |  |
| ETNK2 | 127.078 | -0.98 | 0.24642 | -3.9578 | 7.56E-05 |  |
| GRID1 | 323.149 | -0.98 | 0.3352 | -2.9097 | 0.00362 |  |
| LOC154761 | 127.732 | -0.98 | 0.22867 | -4.2667 | 1.98E-05 |  |
| MYH9 | 22367.2 | -0.98 | 0.1277 | -7.6533 | 1.96E-14 |  |
| PCSK9 | 564.222 | -0.98 | 0.27478 | -3.56 | 0.00037 |  |
| CLDN1 | 8824.74 | -0.98 | 0.16875 | -5.7973 | 6.74E-09 |  |
| ZBTB46 | 117.687 | -0.98 | 0.27251 | -3.5907 | 0.00033 |  |
| FZR1 | 1454.23 | -0.98 | 0.20053 | -4.8872 | 1.02E-06 |  |
| EDIL3 | 20.8198 | -0.98 | 0.3512 | -2.793 | 0.00522 |  |
| SLC7A5 | 29251.9 | -0.98 | 0.19748 | -4.9679 | 6.77E-07 |  |
| KLHL21 | 929.348 | -0.98 | 0.18416 | -5.329 | 9.88E-08 |  |
| SLC16A2 | 50.9281 | -0.98 | 0.29841 | -3.2921 | 0.00099 |  |
| SAMD4A | 608.237 | -0.98 | 0.17674 | -5.56 | 2.70E-08 |  |
| TNFRSF21 | 2401 | -0.99 | 0.22338 | -4.4133 | 1.02E-05 |  |
| TMEM74B | 26.2285 | -0.99 | 0.35876 | -2.7501 | 0.00596 |  |
| DNAH17 | 294.962 | -0.99 | 0.19233 | -5.1305 | 2.89E-07 |  |
| SNTA1 | 376.195 | -0.99 | 0.19795 | -4.9896 | 6.05E-07 |  |
| TCF7L1 | 217.81 | -0.99 | 0.21124 | -4.6788 | 2.89E-06 |  |
| PLCD1 | 301.935 | -0.99 | 0.24949 | -3.9688 | 7.22E-05 |  |
| KRT34 | 16.8975 | -0.99 | 0.39211 | -2.5265 | 0.01152 |  |
| SCARA3 | 2259.63 | -0.99 | 0.20187 | -4.9179 | 8.75E-07 |  |
| JUN | 4229.37 | -0.99 | 0.14632 | -6.7865 | 1.15E-11 |  |
| SYNC | 19.8829 | -0.99 | 0.37911 | -2.6209 | 0.00877 |  |
| TMCC1 | 2423.3 | -0.99 | 0.22452 | -4.4277 | 9.52E-06 |  |
| ART4 | 49.1847 | -0.99 | 0.39388 | -2.525 | 0.01157 |  |
| EFNA2 | 602.732 | -1.00 | 0.30612 | -3.2505 | 0.00115 |  |
| MAPK8IP3 | 6700.37 | -1.00 | 0.17027 | -5.8453 | 5.06E-09 |  |
| PITPNC1 | 73.7062 | -1.00 | 0.28129 | -3.5398 | 0.0004 |  |
| RAG1 | 30.6965 | -1.00 | 0.36937 | -2.6988 | 0.00696 |  |
| CRCT1 | 82.3726 | -1.00 | 0.38464 | -2.5947 | 0.00947 |  |
| CSRNP1 | 823.547 | -1.00 | 0.18139 | -5.5224 | 3.34E-08 |  |
| SPAG4 | 107.457 | -1.00 | 0.23506 | -4.271 | 1.95E-05 |  |
| FUT11 | 829.301 | -1.01 | 0.29162 | -3.4471 | 0.00057 |  |
| HSPA2 | 249.666 | -1.01 | 0.21021 | -4.7857 | 1.70E-06 |  |
| FBN1 | 64.6988 | -1.01 | 0.36317 | -2.7705 | 0.0056 |  |
| FBLIM1 | 2854.6 | -1.01 | 0.15414 | -6.5332 | 6.44E-11 |  |
| CAP1 | 6505.5 | -1.01 | 0.16998 | -5.9365 | 2.91E-09 |  |
| AKT3 | 144.524 | -1.01 | 0.3639 | -2.7731 | 0.00555 |  |
| MIR181A2HG | 21.0133 | -1.01 | 0.38249 | -2.6387 | 0.00832 |  |
| FADS2 | 13061.1 | -1.01 | 0.16096 | -6.2723 | 3.56E-10 |  |
| TGFBR1 | 2476.81 | -1.01 | 0.20703 | -4.88 | 1.06E-06 |  |
| MICAL3 | 1068.48 | -1.01 | 0.15363 | -6.5795 | 4.72E-11 |  |
| KCNA7 | 79.4947 | -1.01 | 0.39142 | -2.5892 | 0.00962 |  |
| CAV2 | 1968.91 | -1.01 | 0.19555 | -5.1873 | 2.13E-07 |  |
| NEK10 | 11.9658 | -1.01 | 0.39325 | -2.5805 | 0.00987 |  |
| PCDHGB5 | 549.61 | -1.02 | 0.22392 | -4.5434 | 5.53E-06 |  |
| TSC22D3 | 3471.17 | -1.02 | 0.15269 | -6.6643 | 2.66E-11 |  |
| ADAMTS16 | 48.6151 | -1.02 | 0.32789 | -3.1043 | 0.00191 |  |
| SPRR3 | 30.212 | -1.02 | 0.38072 | -2.676 | 0.00745 |  |
| EEPD1 | 392.968 | -1.02 | 0.18043 | -5.6512 | 1.59E-08 |  |
| SLMO1 | 70.3105 | -1.02 | 0.32068 | -3.1817 | 0.00146 |  |
| PLCB4 | 251.032 | -1.02 | 0.19113 | -5.3408 | 9.25E-08 |  |
| SOX8 | 11.7864 | -1.02 | 0.3939 | -2.5935 | 0.0095 |  |
| PEA15 | 3415.77 | -1.02 | 0.1787 | -5.7217 | 1.05E-08 |  |
| MOB3B | 1214.78 | -1.02 | 0.19518 | -5.2414 | 1.59E-07 |  |
| FAM178B | 9.45411 | -1.02 | 0.39394 | -2.6017 | 0.00928 |  |
| PCDHGB7 | 113.57 | -1.03 | 0.22928 | -4.4715 | 7.77E-06 |  |
| AFAP1L1 | 357.78 | -1.03 | 0.26285 | -3.9043 | 9.45E-05 |  |
| ITGA10 | 72.0734 | -1.03 | 0.33793 | -3.0394 | 0.00237 |  |
| LOXL1 | 177.577 | -1.03 | 0.2634 | -3.9013 | 9.57E-05 |  |
| PNCK | 28.7961 | -1.03 | 0.38393 | -2.6767 | 0.00743 |  |
| DIRAS1 | 84.0649 | -1.03 | 0.32418 | -3.1713 | 0.00152 |  |
| PCDHGA4 | 70.1927 | -1.03 | 0.27592 | -3.7337 | 0.00019 |  |
| AKAP12 | 2267.67 | -1.03 | 0.29869 | -3.4524 | 0.00056 |  |
| PCDHGB1 | 170.643 | -1.03 | 0.24133 | -4.2742 | 1.92E-05 |  |
| LSAMP-AS1 | 14.9326 | -1.03 | 0.38841 | -2.6579 | 0.00786 |  |
| TMSB4X | 25824.7 | -1.03 | 0.17014 | -6.0772 | 1.22E-09 |  |
| RBMS2 | 5801.72 | -1.03 | 0.18297 | -5.6523 | 1.58E-08 |  |
| ADTRP | 42.2976 | -1.03 | 0.3213 | -3.2213 | 0.00128 |  |
| PCBP4 | 568.638 | -1.04 | 0.22638 | -4.5721 | 4.83E-06 |  |
| SLC2A14 | 17.2863 | -1.04 | 0.39376 | -2.6289 | 0.00857 |  |
| FANK1 | 71.8269 | -1.04 | 0.34065 | -3.0424 | 0.00235 |  |
| AUTS2 | 43.8218 | -1.04 | 0.33101 | -3.1355 | 0.00172 |  |
| SH3PXD2B | 2429.03 | -1.04 | 0.20007 | -5.195 | 2.05E-07 |  |
| SMURF2 | 540.716 | -1.04 | 0.19713 | -5.2801 | 1.29E-07 |  |
| GRK5 | 139.834 | -1.04 | 0.26698 | -3.9001 | 9.61E-05 |  |
| ADM | 1742.93 | -1.04 | 0.23153 | -4.4974 | 6.88E-06 |  |
| LINC00312 | 21.5916 | -1.04 | 0.37215 | -2.8001 | 0.00511 |  |
| PLAG1 | 100.538 | -1.04 | 0.3409 | -3.0581 | 0.00223 |  |
| FZD2 | 121.876 | -1.04 | 0.27903 | -3.7377 | 0.00019 |  |
| TRIM9 | 32.8467 | -1.04 | 0.34126 | -3.0596 | 0.00222 |  |
| RBM24 | 28.1121 | -1.05 | 0.35143 | -2.9753 | 0.00293 |  |
| NUAK1 | 552.245 | -1.05 | 0.2336 | -4.4829 | 7.36E-06 |  |
| MXRA7 | 1322.76 | -1.05 | 0.16547 | -6.3339 | 2.39E-10 |  |
| NPC1 | 867.4 | -1.05 | 0.14483 | -7.2468 | 4.27E-13 |  |
| SLC4A3 | 80.6256 | -1.05 | 0.29154 | -3.6024 | 0.00032 |  |
| SLC25A37 | 1317.4 | -1.05 | 0.15185 | -6.9184 | 4.57E-12 |  |
| DSE | 63.5939 | -1.05 | 0.36857 | -2.8521 | 0.00434 |  |
| CASC10 | 78.6532 | -1.05 | 0.3203 | -3.2822 | 0.00103 |  |
| TTYH3 | 2341.41 | -1.05 | 0.17067 | -6.1601 | 7.27E-10 |  |
| BLACAT1 | 23.8967 | -1.05 | 0.37491 | -2.8065 | 0.00501 |  |
| SERPINB9 | 338.239 | -1.05 | 0.16036 | -6.5728 | 4.94E-11 |  |
| FOSB | 288.037 | -1.05 | 0.2985 | -3.5334 | 0.00041 |  |
| ERRFI1 | 3498.63 | -1.06 | 0.14007 | -7.5387 | 4.75E-14 |  |
| SH3TC2 | 43.8181 | -1.06 | 0.35618 | -2.9654 | 0.00302 |  |
| ENKUR | 69.0156 | -1.06 | 0.30304 | -3.4871 | 0.00049 |  |
| XYLT1 | 102.951 | -1.06 | 0.34983 | -3.0254 | 0.00248 |  |
| FBXO2 | 125.264 | -1.06 | 0.32971 | -3.2144 | 0.00131 |  |
| GPER1 | 805.448 | -1.07 | 0.23044 | -4.6361 | 3.55E-06 |  |
| GALNT2 | 2931.92 | -1.07 | 0.15226 | -7.024 | 2.16E-12 |  |
| ELK3 | 1699.42 | -1.07 | 0.1679 | -6.3748 | 1.83E-10 |  |
| FBN3 | 10.8247 | -1.07 | 0.39403 | -2.7208 | 0.00651 |  |
| SH3D21 | 913.854 | -1.07 | 0.19509 | -5.502 | 3.75E-08 |  |
| PSG5 | 96.4263 | -1.08 | 0.39237 | -2.7432 | 0.00608 |  |
| ZFP36L1 | 3818.75 | -1.08 | 0.10638 | -10.122 | 4.43E-24 |  |
| NEDD9 | 1002.7 | -1.08 | 0.2291 | -4.7018 | 2.58E-06 |  |
| TNFRSF11B | 20.8884 | -1.08 | 0.37331 | -2.8857 | 0.00391 |  |
| ITGB4 | 32558.8 | -1.08 | 0.22355 | -4.8208 | 1.43E-06 |  |
| ERO1A | 8842.36 | -1.08 | 0.20676 | -5.2165 | 1.82E-07 |  |
| FURIN | 4247.05 | -1.08 | 0.21885 | -4.9294 | 8.25E-07 |  |
| FOXF2 | 13.9411 | -1.08 | 0.39307 | -2.7491 | 0.00598 |  |
| ANO6 | 3127.94 | -1.08 | 0.18072 | -5.9856 | 2.16E-09 |  |
| PIK3IP1 | 164.262 | -1.08 | 0.21932 | -4.9376 | 7.91E-07 |  |
| FER1L4 | 146.301 | -1.08 | 0.28945 | -3.7428 | 0.00018 |  |
| ALOX5AP | 102.66 | -1.08 | 0.28086 | -3.8602 | 0.00011 |  |
| KNDC1 | 157.864 | -1.09 | 0.36141 | -3.0045 | 0.00266 |  |
| PRKACB | 562.31 | -1.09 | 0.27696 | -3.9261 | 8.63E-05 |  |
| SCG3 | 312.916 | -1.09 | 0.19345 | -5.6224 | 1.88E-08 |  |
| GLIPR1 | 54.9173 | -1.09 | 0.34481 | -3.1547 | 0.00161 |  |
| TBKBP1 | 109.351 | -1.09 | 0.25851 | -4.2098 | 2.56E-05 |  |
| CLIC3 | 1605.91 | -1.09 | 0.28791 | -3.783 | 0.00015 |  |
| PLAUR | 5833.66 | -1.09 | 0.1997 | -5.4554 | 4.89E-08 |  |
| HOMER3 | 561.258 | -1.09 | 0.21156 | -5.1531 | 2.56E-07 |  |
| FLJ23867 | 139.794 | -1.09 | 0.28451 | -3.8331 | 0.00013 |  |
| VEGFA | 4557.48 | -1.09 | 0.25798 | -4.2289 | 2.35E-05 |  |
| LHX8 | 27.7656 | -1.09 | 0.36891 | -2.9607 | 0.00307 |  |
| CREG2 | 16.3287 | -1.09 | 0.38826 | -2.8152 | 0.00487 |  |
| UBASH3B | 886.205 | -1.09 | 0.26815 | -4.0788 | 4.53E-05 |  |
| INHA | 66.4478 | -1.10 | 0.35528 | -3.0838 | 0.00204 |  |
| CCL24 | 60.2447 | -1.10 | 0.3708 | -2.9552 | 0.00312 |  |
| LOC100288911 | 24.3371 | -1.10 | 0.38692 | -2.8345 | 0.00459 |  |
| SEC14L2 | 151.366 | -1.10 | 0.22649 | -4.8424 | 1.28E-06 |  |
| KCNH1 | 29.6751 | -1.10 | 0.36339 | -3.0182 | 0.00254 |  |
| KISS1R | 46.7309 | -1.10 | 0.3938 | -2.7904 | 0.00526 |  |
| TNC | 18.7788 | -1.10 | 0.39394 | -2.7902 | 0.00527 |  |
| NYNRIN | 125.842 | -1.10 | 0.27917 | -3.9442 | 8.01E-05 |  |
| MIR210HG | 471.907 | -1.10 | 0.16692 | -6.5972 | 4.19E-11 |  |
| WTIP | 399.886 | -1.10 | 0.2811 | -3.9199 | 8.86E-05 |  |
| NFIX | 288.503 | -1.10 | 0.25986 | -4.2478 | 2.16E-05 |  |
| C8orf58 | 418.285 | -1.11 | 0.22286 | -4.9638 | 6.91E-07 |  |
| MFSD12 | 2499.77 | -1.11 | 0.24871 | -4.4527 | 8.48E-06 |  |
| SBK1 | 138.502 | -1.11 | 0.30393 | -3.6454 | 0.00027 |  |
| NATD1 | 347.38 | -1.11 | 0.19404 | -5.7122 | 1.12E-08 |  |
| MT1X | 643.591 | -1.11 | 0.20138 | -5.5048 | 3.70E-08 |  |
| TNFRSF10D | 777.274 | -1.11 | 0.1517 | -7.3121 | 2.63E-13 |  |
| THBD | 1289.33 | -1.11 | 0.19418 | -5.7147 | 1.10E-08 |  |
| LOC100505942 | 23.4072 | -1.11 | 0.38624 | -2.8747 | 0.00404 |  |
| MASP1 | 20.1215 | -1.11 | 0.37805 | -2.9375 | 0.00331 |  |
| B3GNT4 | 77.2246 | -1.11 | 0.30011 | -3.7115 | 0.00021 |  |
| BNC1 | 21.8855 | -1.11 | 0.39398 | -2.828 | 0.00468 |  |
| NAV1 | 233.119 | -1.11 | 0.18385 | -6.0623 | 1.34E-09 |  |
| GPR132 | 71.1906 | -1.12 | 0.36518 | -3.0636 | 0.00219 |  |
| TEAD2 | 671.669 | -1.12 | 0.18829 | -5.9466 | 2.74E-09 |  |
| WIPF1 | 1697.44 | -1.12 | 0.18846 | -5.9426 | 2.81E-09 |  |
| TNFAIP8 | 951.931 | -1.12 | 0.31648 | -3.549 | 0.00039 |  |
| EFR3B | 55.3141 | -1.13 | 0.32945 | -3.4195 | 0.00063 |  |
| VGLL3 | 811.587 | -1.13 | 0.27997 | -4.0271 | 5.65E-05 |  |
| DUOXA1 | 22.2963 | -1.13 | 0.36993 | -3.0524 | 0.00227 |  |
| KDM3A | 1900.05 | -1.13 | 0.14499 | -7.8069 | 5.86E-15 |  |
| CYTH3 | 754.598 | -1.14 | 0.20223 | -5.6357 | 1.74E-08 |  |
| ARID3A | 961.316 | -1.14 | 0.28476 | -4.0024 | 6.27E-05 |  |
| LRRC8A | 5082.64 | -1.14 | 0.2058 | -5.5471 | 2.91E-08 |  |
| UACA | 1399.52 | -1.14 | 0.2067 | -5.5263 | 3.27E-08 |  |
| HDAC5 | 365.319 | -1.15 | 0.23667 | -4.8463 | 1.26E-06 |  |
| A4GALT | 225.095 | -1.15 | 0.37102 | -3.0948 | 0.00197 |  |
| SERPINA1 | 403.812 | -1.15 | 0.35505 | -3.2448 | 0.00118 |  |
| ITGA2 | 1570.69 | -1.15 | 0.2719 | -4.2436 | 2.20E-05 |  |
| IL20RB | 1234.79 | -1.16 | 0.24305 | -4.7536 | 2.00E-06 |  |
| ARHGAP23 | 1628.58 | -1.16 | 0.26 | -4.455 | 8.39E-06 |  |
| LOC100507487 | 110.51 | -1.16 | 0.25157 | -4.6046 | 4.13E-06 |  |
| RASSF5 | 110.625 | -1.16 | 0.34377 | -3.373 | 0.00074 |  |
| NLGN2 | 45.0581 | -1.16 | 0.31787 | -3.6509 | 0.00026 |  |
| TPM1 | 6210.99 | -1.16 | 0.14883 | -7.8158 | 5.46E-15 |  |
| TNNC1 | 289.716 | -1.17 | 0.29587 | -3.9375 | 8.23E-05 |  |
| CDK5R1 | 197.279 | -1.17 | 0.31531 | -3.7162 | 0.0002 |  |
| EPPK1 | 5173.84 | -1.17 | 0.19993 | -5.8634 | 4.54E-09 |  |
| VIM-AS1 | 27.1008 | -1.17 | 0.36525 | -3.2096 | 0.00133 |  |
| PORCN | 153.61 | -1.17 | 0.30793 | -3.8103 | 0.00014 |  |
| ULK1 | 3486.37 | -1.18 | 0.19403 | -6.0659 | 1.31E-09 |  |
| KIAA1549L | 44.6902 | -1.18 | 0.35539 | -3.3121 | 0.00093 |  |
| PLXND1 | 6383.34 | -1.18 | 0.27788 | -4.2395 | 2.24E-05 |  |
| ITGAV | 2423.93 | -1.18 | 0.19469 | -6.0687 | 1.29E-09 |  |
| RAI14 | 922.97 | -1.18 | 0.16172 | -7.3107 | 2.66E-13 |  |
| CALY | 229.78 | -1.18 | 0.34714 | -3.4079 | 0.00065 |  |
| NTF4 | 81.4834 | -1.19 | 0.34383 | -3.4485 | 0.00056 |  |
| HBEGF | 702.21 | -1.19 | 0.28538 | -4.157 | 3.22E-05 |  |
| LAPTM5 | 27.1913 | -1.19 | 0.38736 | -3.0672 | 0.00216 |  |
| SDC4 | 6332.89 | -1.19 | 0.18344 | -6.4833 | 8.97E-11 |  |
| FAM101B | 296.74 | -1.19 | 0.22375 | -5.3171 | 1.05E-07 |  |
| PSG2 | 77.1339 | -1.19 | 0.39395 | -3.0238 | 0.0025 |  |
| KRT14 | 10.9419 | -1.19 | 0.38471 | -3.0969 | 0.00196 |  |
| CGB7 | 26.3613 | -1.19 | 0.37032 | -3.2186 | 0.00129 |  |
| IGSF9 | 27.0899 | -1.19 | 0.38093 | -3.1365 | 0.00171 |  |
| ABL2 | 2786.61 | -1.20 | 0.16412 | -7.282 | 3.29E-13 |  |
| COL3A1 | 24.7299 | -1.20 | 0.38683 | -3.0923 | 0.00199 |  |
| PLEK2 | 2393.36 | -1.20 | 0.19677 | -6.0804 | 1.20E-09 |  |
| NGEF | 1568.27 | -1.20 | 0.15383 | -7.779 | 7.31E-15 |  |
| AMZ1 | 38.1772 | -1.20 | 0.34015 | -3.5187 | 0.00043 |  |
| KLF7 | 4053.37 | -1.20 | 0.1178 | -10.161 | 2.96E-24 |  |
| MSRB3 | 572.622 | -1.20 | 0.20167 | -5.9407 | 2.84E-09 |  |
| PHLDA1 | 1736.73 | -1.20 | 0.26538 | -4.5299 | 5.90E-06 |  |
| LINC-ROR | 57.7266 | -1.21 | 0.37121 | -3.2502 | 0.00115 |  |
| TENM3 | 369.807 | -1.21 | 0.25787 | -4.6799 | 2.87E-06 |  |
| JARID2 | 344.101 | -1.21 | 0.1717 | -7.0347 | 2.00E-12 |  |
| KCTD16 | 17.7256 | -1.21 | 0.39005 | -3.0988 | 0.00194 |  |
| CNN1 | 10.2201 | -1.21 | 0.37822 | -3.1986 | 0.00138 |  |
| SLC6A16 | 91.8338 | -1.21 | 0.3473 | -3.4872 | 0.00049 |  |
| PRSS53 | 70.3924 | -1.21 | 0.2978 | -4.073 | 4.64E-05 |  |
| PCDHGA11 | 147.919 | -1.21 | 0.24833 | -4.888 | 1.02E-06 |  |
| ADAMTSL5 | 655.553 | -1.21 | 0.27812 | -4.3653 | 1.27E-05 |  |
| TCN1 | 26.2386 | -1.22 | 0.3893 | -3.1273 | 0.00176 |  |
| CLDN14 | 85.142 | -1.22 | 0.29231 | -4.1736 | 3.00E-05 |  |
| FAM171A1 | 446.041 | -1.22 | 0.23657 | -5.1572 | 2.51E-07 |  |
| C10orf55 | 20.5596 | -1.22 | 0.37337 | -3.2687 | 0.00108 |  |
| RNF24 | 88.6877 | -1.22 | 0.26642 | -4.5829 | 4.59E-06 |  |
| NDRG1 | 10118.9 | -1.22 | 0.18652 | -6.5619 | 5.31E-11 |  |
| RRAD | 46.6408 | -1.23 | 0.38693 | -3.1688 | 0.00153 |  |
| MATN2 | 1942.63 | -1.23 | 0.20714 | -5.9201 | 3.22E-09 |  |
| EFEMP1 | 53.7656 | -1.23 | 0.34763 | -3.5324 | 0.00041 |  |
| C2orf82 | 553.631 | -1.23 | 0.19835 | -6.1927 | 5.91E-10 |  |
| SERTAD4-AS1 | 57.7941 | -1.23 | 0.32669 | -3.767 | 0.00017 |  |
| SCN5A | 24.9636 | -1.23 | 0.39344 | -3.1376 | 0.0017 |  |
| ZSWIM6 | 324.487 | -1.24 | 0.20214 | -6.1128 | 9.79E-10 |  |
| MFGE8 | 1086.07 | -1.24 | 0.18909 | -6.5435 | 6.01E-11 |  |
| SERPINE2 | 670.205 | -1.24 | 0.25503 | -4.8535 | 1.21E-06 |  |
| RGMA | 159.994 | -1.24 | 0.32862 | -3.7714 | 0.00016 |  |
| SLC12A3 | 23.8325 | -1.24 | 0.38499 | -3.2247 | 0.00126 |  |
| PPP1R3B | 1483.45 | -1.24 | 0.147 | -8.4491 | 2.93E-17 |  |
| GFPT2 | 27.3328 | -1.24 | 0.37777 | -3.2889 | 0.00101 |  |
| PDGFC | 994.473 | -1.25 | 0.18184 | -6.8481 | 7.48E-12 |  |
| LPPR3 | 31.3261 | -1.25 | 0.37196 | -3.3539 | 0.0008 |  |
| SLC7A1 | 6962.1 | -1.25 | 0.1601 | -7.8352 | 4.68E-15 |  |
| RAB31 | 88.5058 | -1.26 | 0.2879 | -4.3673 | 1.26E-05 |  |
| ZEB1 | 247.029 | -1.26 | 0.20623 | -6.0976 | 1.08E-09 |  |
| AQP7P3 | 13.0373 | -1.26 | 0.39398 | -3.1935 | 0.00141 |  |
| VLDLR | 172.449 | -1.26 | 0.22559 | -5.5797 | 2.41E-08 |  |
| COL1A2 | 47.0079 | -1.26 | 0.37671 | -3.3413 | 0.00083 |  |
| B4GALNT2 | 28.4671 | -1.26 | 0.38389 | -3.2822 | 0.00103 |  |
| UCN2 | 11.0468 | -1.26 | 0.38819 | -3.2485 | 0.00116 |  |
| FAT4 | 403.909 | -1.27 | 0.31378 | -4.0317 | 5.54E-05 |  |
| ESAM | 56.0141 | -1.27 | 0.31378 | -4.0349 | 5.46E-05 |  |
| NDST1 | 5085.5 | -1.27 | 0.17783 | -7.1233 | 1.05E-12 |  |
| PTPRF | 5472.98 | -1.27 | 0.16072 | -7.8851 | 3.14E-15 |  |
| PNMA2 | 445.062 | -1.27 | 0.26792 | -4.7374 | 2.16E-06 |  |
| FHL3 | 239.825 | -1.27 | 0.24507 | -5.196 | 2.04E-07 |  |
| WNT9A | 181.227 | -1.28 | 0.30971 | -4.1213 | 3.77E-05 |  |
| BNIP3L | 2891.29 | -1.28 | 0.20607 | -6.2205 | 4.95E-10 |  |
| HDAC9 | 92.6579 | -1.28 | 0.28934 | -4.4331 | 9.29E-06 |  |
| ITGB6 | 554.374 | -1.28 | 0.33659 | -3.8118 | 0.00014 |  |
| EFEMP2 | 243.363 | -1.29 | 0.30662 | -4.1933 | 2.75E-05 |  |
| PLK2 | 841.568 | -1.29 | 0.19653 | -6.5593 | 5.41E-11 |  |
| ITGA5 | 353.198 | -1.29 | 0.31659 | -4.0756 | 4.59E-05 |  |
| AMIGO2 | 9725.82 | -1.29 | 0.23474 | -5.5028 | 3.74E-08 |  |
| COL4A4 | 4263.75 | -1.29 | 0.12472 | -10.381 | 3.02E-25 |  |
| TMEM200C | 56.3292 | -1.29 | 0.39184 | -3.3047 | 0.00095 |  |
| CTSV | 258.027 | -1.30 | 0.23156 | -5.5957 | 2.20E-08 |  |
| RGL1 | 136.974 | -1.30 | 0.25697 | -5.0438 | 4.56E-07 |  |
| SGK1 | 250.052 | -1.30 | 0.2876 | -4.5119 | 6.42E-06 |  |
| CDK14 | 455.769 | -1.30 | 0.24465 | -5.3118 | 1.09E-07 |  |
| STC1 | 9.46737 | -1.30 | 0.38518 | -3.3771 | 0.00073 |  |
| FIBCD1 | 118.448 | -1.30 | 0.27224 | -4.7847 | 1.71E-06 |  |
| TNXB | 298.994 | -1.30 | 0.26245 | -4.9723 | 6.61E-07 |  |
| FSTL3 | 14189.9 | -1.31 | 0.22198 | -5.8995 | 3.65E-09 |  |
| ANXA8L1 | 1043.11 | -1.31 | 0.25474 | -5.1448 | 2.68E-07 |  |
| EGFR | 1836.73 | -1.31 | 0.22107 | -5.9376 | 2.89E-09 |  |
| GPR157 | 172.587 | -1.31 | 0.27842 | -4.7164 | 2.40E-06 |  |
| TSPAN5 | 19.1716 | -1.31 | 0.37977 | -3.4625 | 0.00054 |  |
| CDH3 | 108.113 | -1.32 | 0.32276 | -4.075 | 4.60E-05 |  |
| ATP10A | 3352.54 | -1.32 | 0.37902 | -3.4907 | 0.00048 |  |
| NID1 | 996.958 | -1.33 | 0.27384 | -4.8386 | 1.31E-06 |  |
| PLEKHG2 | 1926.93 | -1.33 | 0.16594 | -8.0004 | 1.24E-15 |  |
| COL7A1 | 1104.16 | -1.33 | 0.28573 | -4.6475 | 3.36E-06 |  |
| AFAP1L2 | 920.405 | -1.33 | 0.30656 | -4.3344 | 1.46E-05 |  |
| ENO2 | 811.167 | -1.33 | 0.39385 | -3.3747 | 0.00074 |  |
| RPS6KA2 | 116.798 | -1.33 | 0.25749 | -5.1672 | 2.38E-07 |  |
| RUSC2 | 1207.01 | -1.33 | 0.16877 | -7.887 | 3.09E-15 |  |
| TANC2 | 1751.11 | -1.33 | 0.18665 | -7.1383 | 9.45E-13 |  |
| LINC00941 | 179.012 | -1.33 | 0.28799 | -4.6355 | 3.56E-06 |  |
| PPP1R18 | 2207.73 | -1.34 | 0.26535 | -5.0311 | 4.88E-07 |  |
| ADAMTS1 | 1118.04 | -1.34 | 0.18735 | -7.1305 | 1.00E-12 |  |
| SLC2A1 | 14762.6 | -1.34 | 0.27265 | -4.9145 | 8.90E-07 |  |
| CASC8 | 37.1019 | -1.34 | 0.39048 | -3.438 | 0.00059 |  |
| C8orf46 | 22.1642 | -1.34 | 0.38111 | -3.5253 | 0.00042 |  |
| LIPG | 69.7418 | -1.34 | 0.36801 | -3.6527 | 0.00026 |  |
| GRIN3B | 72.2013 | -1.35 | 0.36233 | -3.7131 | 0.0002 |  |
| ZYX | 6588.69 | -1.35 | 0.13796 | -9.7607 | 1.66E-22 |  |
| PALM | 1265.73 | -1.35 | 0.2921 | -4.6102 | 4.02E-06 |  |
| TSPAN2 | 17.1379 | -1.35 | 0.39387 | -3.421 | 0.00062 |  |
| MICALL1 | 1516.18 | -1.35 | 0.13527 | -9.9712 | 2.04E-23 |  |
| GSN | 3389.16 | -1.35 | 0.23779 | -5.6792 | 1.35E-08 |  |
| NDUFA4L2 | 313.306 | -1.35 | 0.33097 | -4.0821 | 4.46E-05 |  |
| SYDE1 | 31.6906 | -1.35 | 0.37581 | -3.5986 | 0.00032 |  |
| CYP26B1 | 392.679 | -1.36 | 0.2095 | -6.4779 | 9.30E-11 |  |
| AHNAK2 | 34845.2 | -1.36 | 0.20255 | -6.7198 | 1.82E-11 |  |
| USP54 | 2029.61 | -1.37 | 0.22279 | -6.1575 | 7.39E-10 |  |
| ACKR2 | 67.9232 | -1.37 | 0.37056 | -3.7053 | 0.00021 |  |
| CDH11 | 238.109 | -1.38 | 0.21529 | -6.3895 | 1.66E-10 |  |
| RASSF4 | 82.2755 | -1.38 | 0.35359 | -3.9038 | 9.47E-05 |  |
| C15orf52 | 2712.63 | -1.38 | 0.16012 | -8.6244 | 6.44E-18 |  |
| RTN2 | 229.779 | -1.38 | 0.22064 | -6.2723 | 3.56E-10 |  |
| COX6B2 | 87.4971 | -1.39 | 0.30335 | -4.5705 | 4.87E-06 |  |
| KRT5 | 49.8824 | -1.39 | 0.37629 | -3.6868 | 0.00023 |  |
| NTN4 | 485.444 | -1.39 | 0.32747 | -4.2476 | 2.16E-05 |  |
| PRR5L | 249.067 | -1.39 | 0.28219 | -4.934 | 8.05E-07 |  |
| ABTB2 | 473.504 | -1.39 | 0.16147 | -8.6296 | 6.16E-18 |  |
| CXCR1 | 11.2161 | -1.40 | 0.39127 | -3.5666 | 0.00036 |  |
| ZCCHC24 | 361.389 | -1.40 | 0.21479 | -6.4981 | 8.14E-11 |  |
| OSMR | 2747.5 | -1.40 | 0.13579 | -10.288 | 8.01E-25 |  |
| OLFML2A | 85.1139 | -1.40 | 0.38095 | -3.6699 | 0.00024 |  |
| SHC2 | 734.748 | -1.40 | 0.16937 | -8.2557 | 1.51E-16 |  |
| DPYSL2 | 561.6 | -1.40 | 0.28686 | -4.8822 | 1.05E-06 |  |
| PITPNM3 | 79.7438 | -1.40 | 0.2969 | -4.7195 | 2.36E-06 |  |
| PTK7 | 706.034 | -1.41 | 0.23103 | -6.0816 | 1.19E-09 |  |
| CDKN1A | 3941.79 | -1.41 | 0.18802 | -7.4806 | 7.40E-14 |  |
| CSF1R | 22.4717 | -1.41 | 0.38777 | -3.6388 | 0.00027 |  |
| SLAMF9 | 10.9721 | -1.41 | 0.38312 | -3.6905 | 0.00022 |  |
| MFAP4 | 47.2776 | -1.42 | 0.33154 | -4.279 | 1.88E-05 |  |
| PCDH1 | 1006.48 | -1.42 | 0.38986 | -3.643 | 0.00027 |  |
| MFI2 | 405.499 | -1.42 | 0.20055 | -7.085 | 1.39E-12 |  |
| ID3 | 98.6364 | -1.42 | 0.32525 | -4.377 | 1.20E-05 |  |
| PGM2L1 | 287.75 | -1.42 | 0.31053 | -4.5865 | 4.51E-06 |  |
| NPC2 | 2032 | -1.42 | 0.16388 | -8.6938 | 3.50E-18 |  |
| APBB2 | 1151.37 | -1.43 | 0.28279 | -5.0492 | 4.44E-07 |  |
| PFKFB4 | 283.434 | -1.43 | 0.22436 | -6.3736 | 1.85E-10 |  |
| TRPV4 | 348.422 | -1.44 | 0.26053 | -5.5381 | 3.06E-08 |  |
| ARID5B | 47.602 | -1.44 | 0.34055 | -4.2373 | 2.26E-05 |  |
| LAMB3 | 22331 | -1.44 | 0.11371 | -12.705 | 5.57E-37 |  |
| PRSS23 | 9855.15 | -1.45 | 0.36467 | -3.9681 | 7.24E-05 |  |
| COL15A1 | 18.3548 | -1.45 | 0.39185 | -3.6962 | 0.00022 |  |
| LGI3 | 55.5283 | -1.45 | 0.34906 | -4.1499 | 3.33E-05 |  |
| WDR66 | 565.667 | -1.45 | 0.32032 | -4.5248 | 6.04E-06 |  |
| HTR1D | 349.568 | -1.45 | 0.21243 | -6.8385 | 8.00E-12 |  |
| CLDN6 | 52.8949 | -1.45 | 0.31239 | -4.6511 | 3.30E-06 |  |
| SERPINF1 | 858.217 | -1.45 | 0.17126 | -8.4889 | 2.09E-17 |  |
| SHANK3 | 405.177 | -1.46 | 0.21483 | -6.7788 | 1.21E-11 |  |
| EFNA3 | 300.684 | -1.47 | 0.27899 | -5.2529 | 1.50E-07 |  |
| WDR54 | 486.246 | -1.47 | 0.23395 | -6.2657 | 3.71E-10 |  |
| IGFBP7 | 43.0624 | -1.47 | 0.36797 | -3.9861 | 6.72E-05 |  |
| CDH2 | 440.035 | -1.47 | 0.22541 | -6.5078 | 7.63E-11 |  |
| SARDH | 13.5265 | -1.47 | 0.39381 | -3.7335 | 0.00019 |  |
| ADAM12 | 15.1397 | -1.47 | 0.39368 | -3.737 | 0.00019 |  |
| CALD1 | 4451.75 | -1.47 | 0.14549 | -10.122 | 4.41E-24 |  |
| FAM214B | 963.037 | -1.47 | 0.17225 | -8.5503 | 1.23E-17 |  |
| MFAP3L | 177.701 | -1.48 | 0.28437 | -5.1885 | 2.12E-07 |  |
| ANXA8 | 178.981 | -1.48 | 0.28923 | -5.1177 | 3.09E-07 |  |
| IL11 | 119.227 | -1.48 | 0.34562 | -4.2908 | 1.78E-05 |  |
| VANGL2 | 540.52 | -1.48 | 0.16264 | -9.128 | 6.98E-20 |  |
| EFNB2 | 1554.72 | -1.48 | 0.19816 | -7.4921 | 6.78E-14 |  |
| RAET1L | 26.2802 | -1.49 | 0.38214 | -3.9045 | 9.44E-05 |  |
| TRIML2 | 51.2948 | -1.50 | 0.37505 | -3.9864 | 6.71E-05 |  |
| CTIF | 491.136 | -1.50 | 0.21994 | -6.7987 | 1.06E-11 |  |
| FAM171A2 | 26.6474 | -1.50 | 0.37704 | -3.9734 | 7.08E-05 |  |
| CD59 | 6155.96 | -1.50 | 0.14118 | -10.642 | 1.91E-26 |  |
| EMP3 | 6156.19 | -1.50 | 0.14711 | -10.215 | 1.70E-24 |  |
| FOLR3 | 45.1186 | -1.51 | 0.39346 | -3.8343 | 0.00013 |  |
| S100A4 | 3412.06 | -1.52 | 0.28457 | -5.3495 | 8.82E-08 |  |
| TNS1 | 5575.57 | -1.52 | 0.12599 | -12.083 | 1.29E-33 |  |
| TUBA4B | 82.9064 | -1.52 | 0.34407 | -4.4261 | 9.59E-06 |  |
| PDLIM4 | 48.9586 | -1.52 | 0.32269 | -4.7247 | 2.30E-06 |  |
| COL8A1 | 14.3838 | -1.53 | 0.39397 | -3.8825 | 0.0001 |  |
| PLOD2 | 2328.67 | -1.54 | 0.2586 | -5.9463 | 2.74E-09 |  |
| LAMC2 | 39326.3 | -1.54 | 0.15216 | -10.138 | 3.75E-24 |  |
| BHLHE40 | 3656.78 | -1.55 | 0.1756 | -8.8151 | 1.20E-18 |  |
| UBTD1 | 70.7814 | -1.55 | 0.3306 | -4.69 | 2.73E-06 |  |
| MXRA8 | 848.63 | -1.55 | 0.39312 | -3.9446 | 7.99E-05 |  |
| EHD2 | 1289.08 | -1.55 | 0.2937 | -5.2934 | 1.20E-07 |  |
| LTBP1 | 453.142 | -1.56 | 0.28144 | -5.5273 | 3.25E-08 |  |
| HEG1 | 215.559 | -1.56 | 0.26023 | -5.9932 | 2.06E-09 |  |
| KIRREL | 881.684 | -1.56 | 0.25894 | -6.0264 | 1.68E-09 |  |
| ECM1 | 715.625 | -1.56 | 0.28706 | -5.4374 | 5.41E-08 |  |
| FAM65A | 3208.03 | -1.56 | 0.20878 | -7.4781 | 7.54E-14 |  |
| SLIT3 | 539.14 | -1.56 | 0.33196 | -4.7126 | 2.45E-06 |  |
| PDLIM7 | 5709.88 | -1.57 | 0.20016 | -7.819 | 5.32E-15 |  |
| SCD | 14420.6 | -1.57 | 0.2249 | -6.9682 | 3.21E-12 |  |
| CD37 | 68.9842 | -1.58 | 0.34293 | -4.598 | 4.27E-06 |  |
| CLEC16A | 1992.68 | -1.58 | 0.17593 | -8.9704 | 2.95E-19 |  |
| C22orf23 | 360.141 | -1.58 | 0.16441 | -9.6282 | 6.08E-22 |  |
| GLIPR2 | 38.593 | -1.58 | 0.35183 | -4.4997 | 6.80E-06 |  |
| NEURL1B | 2579.06 | -1.59 | 0.26485 | -5.9931 | 2.06E-09 |  |
| ANTXR1 | 38.5531 | -1.59 | 0.3781 | -4.2143 | 2.51E-05 |  |
| PPP1R13L | 3178.05 | -1.59 | 0.24263 | -6.5729 | 4.93E-11 |  |
| LOC101928841 | 1456.81 | -1.60 | 0.20988 | -7.6047 | 2.86E-14 |  |
| SEMA3C | 1666.18 | -1.60 | 0.25005 | -6.3879 | 1.68E-10 |  |
| PYGL | 2985.71 | -1.60 | 0.1484 | -10.766 | 4.97E-27 |  |
| HSPA6 | 93.8581 | -1.60 | 0.35168 | -4.5454 | 5.48E-06 |  |
| DDIT4 | 3754.01 | -1.61 | 0.22311 | -7.197 | 6.15E-13 |  |
| SEMA4A | 124.364 | -1.61 | 0.24608 | -6.5317 | 6.50E-11 |  |
| ABHD12B | 30.3523 | -1.61 | 0.37623 | -4.2796 | 1.87E-05 |  |
| HK2 | 1302.78 | -1.62 | 0.17481 | -9.2468 | 2.31E-20 |  |
| AATK-AS1 | 16.443 | -1.62 | 0.39352 | -4.1099 | 3.96E-05 |  |
| CRIM1 | 2139.06 | -1.62 | 0.24459 | -6.6208 | 3.57E-11 |  |
| CDKN1C | 2146.22 | -1.62 | 0.21944 | -7.3919 | 1.45E-13 |  |
| LINC01260 | 16.6978 | -1.62 | 0.39403 | -4.1191 | 3.80E-05 |  |
| EFNA1 | 9722.69 | -1.62 | 0.24384 | -6.6593 | 2.75E-11 |  |
| MYH16 | 70.3591 | -1.63 | 0.28466 | -5.7172 | 1.08E-08 |  |
| CHST3 | 3954.87 | -1.63 | 0.28907 | -5.6317 | 1.78E-08 |  |
| PTHLH | 77.108 | -1.63 | 0.34153 | -4.7806 | 1.75E-06 |  |
| DCBLD1 | 228.583 | -1.64 | 0.22381 | -7.3186 | 2.50E-13 |  |
| PODXL | 4130.75 | -1.65 | 0.29288 | -5.6178 | 1.93E-08 |  |
| TUBB3 | 2820.17 | -1.65 | 0.21068 | -7.8105 | 5.69E-15 |  |
| RASGRP1 | 88.4558 | -1.65 | 0.28034 | -5.8812 | 4.07E-09 |  |
| CDA | 2008.95 | -1.65 | 0.18343 | -8.9959 | 2.34E-19 |  |
| PIK3CD | 199.774 | -1.65 | 0.26152 | -6.323 | 2.57E-10 |  |
| GPR17 | 30.0677 | -1.65 | 0.36504 | -4.5319 | 5.85E-06 |  |
| PHLDB1 | 1877.9 | -1.65 | 0.18239 | -9.0726 | 1.16E-19 |  |
| MN1 | 394.163 | -1.66 | 0.33966 | -4.8939 | 9.88E-07 |  |
| ADAMTS15 | 59.6525 | -1.66 | 0.30368 | -5.4785 | 4.29E-08 |  |
| LEMD1 | 14.4199 | -1.67 | 0.38777 | -4.2969 | 1.73E-05 |  |
| KCTD11 | 1657.33 | -1.67 | 0.2146 | -7.7777 | 7.39E-15 |  |
| KRT16 | 670.815 | -1.67 | 0.27154 | -6.1521 | 7.64E-10 |  |
| SPTBN5 | 70.2523 | -1.67 | 0.3751 | -4.4551 | 8.39E-06 |  |
| LDLRAD4 | 39.5806 | -1.68 | 0.39349 | -4.2583 | 2.06E-05 |  |
| SMOX | 439.121 | -1.68 | 0.20366 | -8.2604 | 1.45E-16 |  |
| CYP27C1 | 127.918 | -1.68 | 0.35384 | -4.7617 | 1.92E-06 |  |
| ARHGAP30 | 145.064 | -1.69 | 0.24604 | -6.8683 | 6.50E-12 |  |
| SH3RF3 | 16.8113 | -1.69 | 0.39398 | -4.2903 | 1.78E-05 |  |
| VCAN | 21.8273 | -1.69 | 0.39063 | -4.3386 | 1.43E-05 |  |
| BMP1 | 3087.88 | -1.70 | 0.15423 | -11.054 | 2.10E-28 |  |
| ANGPTL4 | 1135.94 | -1.72 | 0.16407 | -10.471 | 1.17E-25 |  |
| TMEM132A | 377.006 | -1.73 | 0.32877 | -5.2627 | 1.42E-07 |  |
| ALDOC | 290.179 | -1.73 | 0.31085 | -5.5813 | 2.39E-08 |  |
| LPAR5 | 503.312 | -1.75 | 0.31084 | -5.6206 | 1.90E-08 |  |
| CLDN9 | 39.5835 | -1.75 | 0.36102 | -4.8493 | 1.24E-06 |  |
| NES | 52.0814 | -1.76 | 0.35682 | -4.9268 | 8.36E-07 |  |
| GRB10 | 2512.62 | -1.76 | 0.20466 | -8.5953 | 8.30E-18 |  |
| NKILA | 82.023 | -1.76 | 0.28785 | -6.1225 | 9.21E-10 |  |
| LINC00862 | 27.8125 | -1.76 | 0.37737 | -4.6703 | 3.01E-06 |  |
| RGCC | 23.7106 | -1.76 | 0.38909 | -4.5305 | 5.89E-06 |  |
| LUM | 72.1819 | -1.77 | 0.38327 | -4.6091 | 4.04E-06 |  |
| LCK | 16.0401 | -1.77 | 0.39339 | -4.4915 | 7.07E-06 |  |
| SCN9A | 740.544 | -1.77 | 0.20745 | -8.5296 | 1.47E-17 |  |
| HSPB1 | 8096.77 | -1.77 | 0.11846 | -14.947 | 1.64E-50 |  |
| KIAA1462 | 105.564 | -1.77 | 0.27196 | -6.5206 | 7.00E-11 |  |
| FERMT2 | 749.447 | -1.78 | 0.16637 | -10.693 | 1.10E-26 |  |
| CCNJL | 189.353 | -1.79 | 0.35625 | -5.0333 | 4.82E-07 |  |
| RBMS3 | 46.9512 | -1.79 | 0.3932 | -4.5624 | 5.06E-06 |  |
| PSG4 | 255.295 | -1.79 | 0.36202 | -4.9564 | 7.18E-07 |  |
| FLNA | 49580 | -1.81 | 0.24338 | -7.4374 | 1.03E-13 |  |
| NXPH4 | 118.364 | -1.81 | 0.34211 | -5.2972 | 1.18E-07 |  |
| ACKR3 | 4113.49 | -1.82 | 0.3731 | -4.8815 | 1.05E-06 |  |
| ATP2B4 | 2158.32 | -1.83 | 0.24647 | -7.4346 | 1.05E-13 |  |
| SPHK1 | 620.529 | -1.84 | 0.30026 | -6.1245 | 9.09E-10 |  |
| ADAMTS17 | 122.615 | -1.89 | 0.3326 | -5.6757 | 1.38E-08 |  |
| ARHGAP31 | 151.429 | -1.89 | 0.2628 | -7.187 | 6.62E-13 |  |
| GPC4 | 406.144 | -1.89 | 0.33178 | -5.7017 | 1.19E-08 |  |
| CADM1 | 419.766 | -1.89 | 0.32501 | -5.8264 | 5.66E-09 |  |
| MAML2 | 141.214 | -1.90 | 0.35866 | -5.2866 | 1.25E-07 |  |
| PNPLA3 | 293.569 | -1.90 | 0.22346 | -8.5139 | 1.68E-17 |  |
| RTL1 | 18.3954 | -1.90 | 0.39228 | -4.8503 | 1.23E-06 |  |
| MYL2 | 14.248 | -1.91 | 0.39245 | -4.858 | 1.19E-06 |  |
| CEP131 | 2559.42 | -1.91 | 0.15638 | -12.23 | 2.16E-34 |  |
| ANKRD37 | 134.453 | -1.93 | 0.2325 | -8.2872 | 1.16E-16 |  |
| ITGA11 | 31.3786 | -1.94 | 0.37038 | -5.2417 | 1.59E-07 |  |
| CMTM3 | 1571.35 | -1.95 | 0.18298 | -10.662 | 1.52E-26 |  |
| MKRN9P | 22.4107 | -1.95 | 0.38352 | -5.0938 | 3.51E-07 |  |
| KCNJ16 | 82.2673 | -1.95 | 0.33933 | -5.7584 | 8.49E-09 |  |
| LRRN2 | 41.2578 | -1.96 | 0.37769 | -5.1822 | 2.19E-07 |  |
| SLC35F3 | 85.723 | -1.96 | 0.32138 | -6.1085 | 1.01E-09 |  |
| SULF2 | 316.474 | -1.96 | 0.29755 | -6.6038 | 4.01E-11 |  |
| GPR87 | 30.1128 | -1.97 | 0.38544 | -5.1069 | 3.27E-07 |  |
| CNN3 | 139.975 | -1.98 | 0.30035 | -6.5921 | 4.34E-11 |  |
| SORCS2 | 51.5737 | -2.00 | 0.39234 | -5.0856 | 3.66E-07 |  |
| SLC2A3 | 3948.13 | -2.00 | 0.39371 | -5.083 | 3.71E-07 |  |
| ST8SIA2 | 33.8577 | -2.01 | 0.37986 | -5.2801 | 1.29E-07 |  |
| LPCAT2 | 576.266 | -2.01 | 0.28864 | -6.9572 | 3.47E-12 |  |
| CLTCL1 | 208.904 | -2.04 | 0.22372 | -9.1115 | 8.13E-20 |  |
| COL6A2 | 4362.79 | -2.07 | 0.24635 | -8.394 | 4.70E-17 |  |
| TUFT1 | 3904.38 | -2.07 | 0.14768 | -14.018 | 1.22E-44 |  |
| LGR6 | 44.7937 | -2.07 | 0.36627 | -5.6533 | 1.57E-08 |  |
| FAM49A | 51.1815 | -2.08 | 0.3566 | -5.819 | 5.92E-09 |  |
| AQP1 | 205.185 | -2.08 | 0.253 | -8.2096 | 2.22E-16 |  |
| CHST11 | 646.321 | -2.08 | 0.29231 | -7.1128 | 1.14E-12 |  |
| TSHZ3 | 17.9516 | -2.08 | 0.3912 | -5.3172 | 1.05E-07 |  |
| MRAS | 295.269 | -2.10 | 0.20473 | -10.235 | 1.39E-24 |  |
| FBLN2 | 43.4847 | -2.11 | 0.38347 | -5.4985 | 3.83E-08 |  |
| FN1 | 391.52 | -2.11 | 0.20382 | -10.366 | 3.54E-25 |  |
| TNFRSF19 | 54.6715 | -2.12 | 0.3492 | -6.0678 | 1.30E-09 |  |
| LAMA3 | 10212.1 | -2.12 | 0.17917 | -11.829 | 2.77E-32 |  |
| TGFB1I1 | 1072.87 | -2.12 | 0.28446 | -7.4511 | 9.26E-14 |  |
| PPFIA4 | 100.776 | -2.13 | 0.28686 | -7.4175 | 1.19E-13 |  |
| MMP11 | 142.812 | -2.13 | 0.30156 | -7.0676 | 1.58E-12 |  |
| KCNJ15 | 39.1014 | -2.15 | 0.34128 | -6.2918 | 3.14E-10 |  |
| PHLDB2 | 361.847 | -2.15 | 0.2862 | -7.5189 | 5.52E-14 |  |
| CSDC2 | 32.9892 | -2.18 | 0.3524 | -6.1926 | 5.92E-10 |  |
| COL22A1 | 236.731 | -2.20 | 0.26573 | -8.2639 | 1.41E-16 |  |
| CACNA1B | 63.2769 | -2.20 | 0.32884 | -6.683 | 2.34E-11 |  |
| ECEL1 | 261.379 | -2.20 | 0.24682 | -8.9166 | 4.81E-19 |  |
| TGFB1 | 649.996 | -2.22 | 0.34278 | -6.4677 | 9.95E-11 |  |
| HAPLN3 | 63.8933 | -2.22 | 0.33226 | -6.6955 | 2.15E-11 |  |
| BDNF | 213.539 | -2.23 | 0.34755 | -6.4187 | 1.37E-10 |  |
| SERPINA3 | 191.221 | -2.23 | 0.38603 | -5.7836 | 7.31E-09 |  |
| PDGFB | 887.021 | -2.24 | 0.29214 | -7.6642 | 1.80E-14 |  |
| GDF6 | 26.4941 | -2.24 | 0.39367 | -5.6972 | 1.22E-08 |  |
| DKK3 | 348.596 | -2.25 | 0.32069 | -7.0096 | 2.39E-12 |  |
| CACNG4 | 225.686 | -2.25 | 0.28394 | -7.9354 | 2.10E-15 |  |
| SLC38A5 | 76.9717 | -2.27 | 0.37183 | -6.093 | 1.11E-09 |  |
| ALPL | 1585.42 | -2.27 | 0.30643 | -7.3962 | 1.40E-13 |  |
| ADAMTSL4 | 519.513 | -2.28 | 0.18304 | -12.452 | 1.36E-35 |  |
| DOCK2 | 29.6503 | -2.28 | 0.3914 | -5.8245 | 5.73E-09 |  |
| PXDNL | 23.7635 | -2.30 | 0.38471 | -5.97 | 2.37E-09 |  |
| BMP6 | 66.0247 | -2.31 | 0.37856 | -6.0912 | 1.12E-09 |  |
| COL4A2 | 13325.5 | -2.31 | 0.26034 | -8.8696 | 7.34E-19 |  |
| PLAU | 2910.68 | -2.31 | 0.29854 | -7.7405 | 9.90E-15 |  |
| ATP8A2 | 100.014 | -2.32 | 0.29294 | -7.918 | 2.41E-15 |  |
| COL4A1 | 4356.8 | -2.33 | 0.26279 | -8.8718 | 7.20E-19 |  |
| SNAI2 | 304.557 | -2.33 | 0.27169 | -8.5869 | 8.93E-18 |  |
| NOX4 | 216.268 | -2.34 | 0.38362 | -6.104 | 1.03E-09 |  |
| SLCO2A1 | 23.4822 | -2.35 | 0.38908 | -6.0282 | 1.66E-09 |  |
| ETS1 | 1180.21 | -2.35 | 0.22566 | -10.414 | 2.14E-25 |  |
| CERCAM | 461.044 | -2.39 | 0.28714 | -8.3171 | 9.01E-17 |  |
| CGB5 | 22.5372 | -2.47 | 0.38802 | -6.3565 | 2.06E-10 |  |
| LINC01279 | 37.5872 | -2.50 | 0.35822 | -6.9857 | 2.84E-12 |  |
| SPOCK1 | 40.6782 | -2.52 | 0.37329 | -6.7473 | 1.51E-11 |  |
| CTGF | 3081.67 | -2.52 | 0.11817 | -21.343 | ####### |  |
| ANKRD1 | 472.547 | -2.55 | 0.34052 | -7.4858 | 7.11E-14 |  |
| CRLF1 | 70.6485 | -2.55 | 0.37965 | -6.7224 | 1.79E-11 |  |
| BIRC7 | 112.986 | -2.57 | 0.32882 | -7.8054 | 5.93E-15 |  |
| PKP1 | 48.32 | -2.61 | 0.39364 | -6.6278 | 3.41E-11 |  |
| C5orf46 | 27.1226 | -2.63 | 0.39123 | -6.7189 | 1.83E-11 |  |
| IGFBP2 | 2241.14 | -2.64 | 0.27655 | -9.5459 | 1.35E-21 |  |
| MFAP5 | 155.531 | -2.65 | 0.38235 | -6.9209 | 4.49E-12 |  |
| EGLN3 | 1748.4 | -2.66 | 0.31449 | -8.4471 | 2.99E-17 |  |
| TUBA1A | 344.559 | -2.67 | 0.20768 | -12.844 | 9.33E-38 |  |
| FRMD6 | 154.241 | -2.67 | 0.25005 | -10.688 | 1.16E-26 |  |
| COL1A1 | 1179.99 | -2.69 | 0.16425 | -16.371 | 3.08E-60 |  |
| ZBED2 | 1196.89 | -2.70 | 0.31603 | -8.5487 | 1.24E-17 |  |
| PMEPA1 | 15420.5 | -2.70 | 0.18668 | -14.474 | 1.77E-47 |  |
| LIMS2 | 312.244 | -2.74 | 0.3027 | -9.0533 | 1.39E-19 |  |
| C10orf10 | 143.127 | -2.77 | 0.30015 | -9.2425 | 2.41E-20 |  |
| EPHA4 | 253.356 | -2.79 | 0.25634 | -10.886 | 1.35E-27 |  |
| TNS4 | 5850.02 | -2.79 | 0.26541 | -10.53 | 6.29E-26 |  |
| COL12A1 | 730.346 | -2.79 | 0.22421 | -12.465 | 1.15E-35 |  |
| LOXL2 | 160.194 | -2.85 | 0.31263 | -9.1287 | 6.93E-20 |  |
| C5AR2 | 141.531 | -2.88 | 0.35385 | -8.141 | 3.92E-16 |  |
| DKFZp434J0226 | 117.367 | -2.88 | 0.25737 | -11.21 | 3.66E-29 |  |
| NPTX1 | 93.5619 | -2.89 | 0.37824 | -7.6318 | 2.32E-14 |  |
| CYR61 | 1019.44 | -2.91 | 0.16394 | -17.74 | 2.06E-70 |  |
| PTRF | 1355.65 | -2.99 | 0.25792 | -11.585 | 4.90E-31 |  |
| LTBP2 | 757.021 | -3.00 | 0.21618 | -13.866 | 1.01E-43 |  |
| RBP1 | 599.415 | -3.03 | 0.26261 | -11.527 | 9.62E-31 |  |
| JPH2 | 98.7935 | -3.04 | 0.34288 | -8.8737 | 7.07E-19 |  |
| LBH | 4310.48 | -3.05 | 0.23642 | -12.902 | 4.40E-38 |  |
| MGAT5B | 271.407 | -3.05 | 0.27287 | -11.195 | 4.32E-29 |  |
| PVRL4 | 638.052 | -3.07 | 0.22281 | -13.78 | 3.35E-43 |  |
| ALPK2 | 148.883 | -3.08 | 0.30456 | -10.112 | 4.89E-24 |  |
| NNMT | 1060.4 | -3.10 | 0.32607 | -9.4968 | 2.16E-21 |  |
| KPRP | 42.6939 | -3.13 | 0.38169 | -8.2129 | 2.16E-16 |  |
| VASN | 253.725 | -3.14 | 0.27925 | -11.235 | 2.75E-29 |  |
| MARCH4         . | 102.967 | -3.16 | 0.32943 | -9.6 | 7.99E-22 |  |
| WNT11 | 1278.36 | -3.22 | 0.29948 | -10.747 | 6.15E-27 |  |
| C6orf15 | 164.622 | -3.24 | 0.37393 | -8.6555 | 4.91E-18 |  |
| FOXS1 | 48.9219 | -3.34 | 0.35408 | -9.438 | 3.80E-21 |  |
| CAV1 | 1648.29 | -3.35 | 0.243 | -13.787 | 3.04E-43 |  |
| FLJ16779 | 58.7289 | -3.43 | 0.35965 | -9.5284 | 1.60E-21 |  |
| WNT5B | 222.36 | -3.45 | 0.26074 | -13.235 | 5.52E-40 |  |
| TGFBI | 5777.58 | -3.49 | 0.16872 | -20.697 | 3.65E-95 |  |
| TGFB2 | 67.8183 | -3.50 | 0.35379 | -9.8798 | 5.09E-23 |  |
| SEMA7A | 1250.26 | -3.53 | 0.30119 | -11.709 | 1.15E-31 |  |
| INHBA | 149.914 | -3.55 | 0.2565 | -13.842 | 1.42E-43 |  |
| FSTL1 | 541.087 | -3.55 | 0.23519 | -15.115 | 1.29E-51 |  |
| IGFL2 | 159.441 | -3.56 | 0.37972 | -9.386 | 6.23E-21 |  |
| MAFB | 96.0318 | -3.69 | 0.34764 | -10.619 | 2.42E-26 |  |
| ADAM19 | 196.352 | -3.83 | 0.28372 | -13.509 | 1.38E-41 |  |
| CGB8 | 96.9067 | -3.89 | 0.34691 | -11.212 | 3.56E-29 |  |
| MYL9 | 1643.86 | -3.95 | 0.16118 | -24.496 | ####### |  |
| PLXDC2 | 548.713 | -3.96 | 0.2038 | -19.445 | 3.20E-84 |  |
| THBS1 | 5006.59 | -4.00 | 0.1605 | -24.926 | ####### |  |
| VIM | 8753.1 | -4.04 | 0.2476 | -16.316 | 7.62E-60 |  |
| BEAN1 | 180.763 | -4.06 | 0.297 | -13.676 | 1.41E-42 |  |
| KRT6A | 9762.24 | -4.45 | 0.27849 | -15.977 | 1.86E-57 |  |
| CST6 | 309.256 | -4.64 | 0.2784 | -16.662 | 2.46E-62 |  |
| EPHB2 | 449.792 | -4.70 | 0.22893 | -20.533 | 1.09E-93 |  |
| TAGLN | 6294.16 | -4.74 | 0.1122 | -42.228 | 0 |  |
| MAF | 772.431 | -4.81 | 0.28011 | -17.182 | 3.63E-66 |  |
| TIMP3 | 580.137 | -4.84 | 0.33607 | -14.4 | 5.20E-47 |  |
| SERPINE1 | 1848.09 | -4.99 | 0.23331 | -21.403 | ####### |  |
| CCDC80 | 347.613 | -5.13 | 0.26108 | -19.656 | 5.18E-86 |  |
| MMP2 | 398.356 | -5.23 | 0.32227 | -16.239 | 2.66E-59 |  |
| KRT17 | 33576.9 | -5.32 | 0.15932 | -33.362 | ####### |  |
| COL5A1 | 1755.34 | -5.83 | 0.28911 | -20.18 | 1.47E-90 |  |
|  |  |  |  |  |  |  |
|  |  |  |  |  |  |  |
|  |  |  |  |  |  |  |
|  |  |  |  |  |  |  |
|  |  |  |  |  |  |  |
|  |  |  |  |  |  |  |
|  |  |  |  |  |  |  |
|  |  |  |  |  |  |  |
|  |  |  |  |  |  |  |
|  |  |  |  |  |  |  |
|  |  |  |  |  |  |  |
|  |  |  |  |  |  |  |
|  |  |  |  |  |  |  |
|  |  |  |  |  |  |  |
|  |  |  |  |  |  |  |
|  |  |  |  |  |  |  |
|  |  |  |  |  |  |  |
|  |  |  |  |  |  |  |
|  |  |  |  |  |  |  |
|  |  |  |  |  |  |  |
|  |  |  |  |  |  |  |
|  |  |  |  |  |  |  |
|  |  |  |  |  |  |  |
|  |  |  |  |  |  |  |
|  |  |  |  |  |  |  |
|  |  |  |  |  |  |  |
|  |  |  |  |  |  |  |
|  |  |  |  |  |  |  |
|  |  |  |  |  |  |  |
|  |  |  |  |  |  |  |
|  |  |  |  |  |  |  |
|  |  |  |  |  |  |  |
|  |  |  |  |  |  |  |
|  |  |  |  |  |  |  |
|  |  |  |  |  |  |  |
|  |  |  |  |  |  |  |
|  |  |  |  |  |  |  |
|  |  |  |  |  |  |  |
|  |  |  |  |  |  |  |
|  |  |  |  |  |  |  |
|  |  |  |  |  |  |  |
|  |  |  |  |  |  |  |
|  |  |  |  |  |  |  |
|  |  |  |  |  |  |  |
|  |  |  |  |  |  |  |
|  |  |  |  |  |  |  |
|  |  |  |  |  |  |  |
|  |  |  |  |  |  |  |
|  |  |  |  |  |  |  |
|  |  |  |  |  |  |  |
|  |  |  |  |  |  |  |
|  |  |  |  |  |  |  |
|  |  |  |  |  |  |  |
|  |  |  |  |  |  |  |
|  |  |  |  |  |  |  |
|  |  |  |  |  |  |  |
|  |  |  |  |  |  |  |
|  |  |  |  |  |  |  |
|  |  |  |  |  |  |  |
|  |  |  |  |  |  |  |
|  |  |  |  |  |  |  |
|  |  |  |  |  |  |  |
|  |  |  |  |  |  |  |
|  |  |  |  |  |  |  |
|  |  |  |  |  |  |  |
|  |  |  |  |  |  |  |
|  |  |  |  |  |  |  |
|  |  |  |  |  |  |  |
|  |  |  |  |  |  |  |
|  |  |  |  |  |  |  |
|  |  |  |  |  |  |  |
|  |  |  |  |  |  |  |
|  |  |  |  |  |  |  |
|  |  |  |  |  |  |  |
|  |  |  |  |  |  |  |
|  |  |  |  |  |  |  |
|  |  |  |  |  |  |  |
|  |  |  |  |  |  |  |
|  |  |  |  |  |  |  |
|  |  |  |  |  |  |  |
|  |  |  |  |  |  |  |
|  |  |  |  |  |  |  |
|  |  |  |  |  |  |  |
|  |  |  |  |  |  |  |
|  |  |  |  |  |  |  |
|  |  |  |  |  |  |  |
|  |  |  |  |  |  |  |
|  |  |  |  |  |  |  |
|  |  |  |  |  |  |  |
|  |  |  |  |  |  |  |
|  |  |  |  |  |  |  |
|  |  |  |  |  |  |  |
|  |  |  |  |  |  |  |
|  |  |  |  |  |  |  |
|  |  |  |  |  |  |  |
|  |  |  |  |  |  |  |
|  |  |  |  |  |  |  |
|  |  |  |  |  |  |  |
|  |  |  |  |  |  |  |
|  |  |  |  |  |  |  |
|  |  |  |  |  |  |  |
