# Supplementary material for: A functional genomic screen in vivo identifies CEACAM5 as a clinically relevant driver of breast cancer metastasis
Source: NPJ Breast Cancer. 2018 Apr 30;4:9. doi: 10.1038/s41523-018-0062-x (PMC5928229; doi:10.1038/s41523-018-0062-x)
Supplement: Supplementary file 3 — Supplementary Table 2 [file 41523_2018_62_MOESM3_ESM.docx]

| rank order (enrichment in lung) | gene | number of mice | ave.M-ref | **ave.diff (ddCT_L-ddCT-M)** | pool_bc |  | rank order (enrichment in lung) | number of mice | ave.M-ref | **ave.diff (ddCT_L-ddCT-M)** | gene | pool_bc |
| --- | --- | --- | --- | --- | --- | --- | --- | --- | --- | --- | --- | --- |
| 1 | DPP4 | 3 | -3.80 | **8.0** | 19_BC5 |  | 1 | 4 | 1.70 | **7.8** | NDRG2 | 10_BC8 |
| 2 | OLFM1 | 4 | -5.65 | **8.0** | 13_BC6 |  | 2 | 5 | 1.11 | **7.3** | MSX2 | 7_BC12 |
| 3 | SLC44A5 | 3 | -1.37 | **7.6** | 19_BC2 |  | 3 | 4 | 1.66 | **7.2** | FOXL1 | 10_BC3 |
| 4 | CEACAM5 | 4 | -3.32 | **7.5** | 18_BC12 |  | 4 | 3 | 1.16 | **6.5** | NAALADL2 | 9_BC5 |
| 5 | LGALS9C | 4 | -1.59 | **7.3** | 10_BC6 |  | 5 | 5 | 3.35 | **6.3** | SMOC1 | 12_BC3 |
| 6 | RAB40B | 4 | -2.26 | **7.3** | 8_BC5 |  | 6 | 3 | 1.50 | **6.3** | STARD10 | 8_BC12 |
| 7 | F7 | 5 | -3.45 | **7.1** | 13_BC5 |  | 7 | 4 | 2.78 | **6.1** | NNAT | 3_BC2 |
| 8 | GIPR | 5 | -3.35 | **6.9** | 13_BC3 |  | 8 | 3 | 1.93 | **6.1** | IRF8 | 11_BC12 |
| 9 | HUNK | 4 | -1.36 | **6.9** | 18_BC13 |  | 9 | 4 | 1.24 | **5.7** | KIAA1147 | 3_BC1 |
| 10 | SLC5A2 | 3 | -3.29 | **6.9** | 18_BC7 |  | 10 | 4 | 1.65 | **5.7** | TFF1 | 3_BC7 |
| 11 | GABRB3 | 5 | -3.33 | **6.9** | 13_BC9 |  | 11 | 5 | 1.42 | **5.2** | HOXC6 | 6_BC10 |
| 12 | GDF15 | 3 | -1.32 | **6.8** | 9_BC2 |  | 12 | 6 | 2.42 | **4.6** | C11orf53 | 6_BC11 |
| 13 | HSD11B2 | 5 | -1.38 | **6.5** | 11_BC8 |  |  |  |  |  |  |  |
| 14 | TUBB4 | 5 | -1.96 | **6.5** | 12_BC4 |  |  |  |  |  |  |  |
| 15 | SHH | 4 | -2.66 | **6.5** | 13_BC2 |  |  |  |  |  |  |  |
| 16 | GLTPD2 | 4 | -1.37 | **6.4** | 8_BC11 |  |  |  |  |  |  |  |
| 17 | CA11 | 3 | -1.61 | **6.4** | 9_BC9 |  |  |  |  |  |  |  |
| 18 | GPA33 | 4 | -2.92 | **6.3** | 9_BC4 |  |  |  |  |  |  |  |
| 19 | FAM155A | 5 | -1.12 | **6.2** | 12_BC8 |  |  |  |  |  |  |  |
| 20 | PHACTR3 | 3 | -1.68 | **6.2** | 12_BC5 |  |  |  |  |  |  |  |
| 21 | SLCO4A1 | 3 | -1.71 | **6.1** | 19_BC3 |  |  |  |  |  |  |  |
| 22 | ZBTB20 | 4 | -4.56 | **6.0** | 18_BC6 |  |  |  |  |  |  |  |
| 23 | C20orf112 | 4 | -1.61 | **6.0** | 11_BC7 |  |  |  |  |  |  |  |
| 24 | PCDHGA6 | 3 | -2.06 | **6.0** | 19_BC9 |  |  |  |  |  |  |  |
| 25 | SLC17A9 | 4 | -1.26 | **5.8** | 11_BC4 |  |  |  |  |  |  |  |
| 26 | BAIAP2L2 | 3 | -4.52 | **5.8** | 15_BC3 |  |  |  |  |  |  |  |
| 27 | C6orf222 | 4 | -1.61 | **5.7** | 18_BC4 |  |  |  |  |  |  |  |
| 28 | ZBED3 | 6 | -2.61 | **5.6** | 6_BC9 |  |  |  |  |  |  |  |
| 29 | EFCAB4A | 4 | -1.57 | **5.5** | 9_BC1 |  |  |  |  |  |  |  |
| 30 | PRRT2 | 3 | -3.97 | **5.5** | 9_BC12 |  |  |  |  |  |  |  |
| 31 | NEU4 | 6 | -2.82 | **5.5** | 14_BC11 |  |  |  |  |  |  |  |
| 32 | CLDN15 | 6 | -1.66 | **5.4** | 6_BC7 |  |  |  |  |  |  |  |
| 33 | TTYH1 | 3 | -2.45 | **5.4** | 7_BC1 |  |  |  |  |  |  |  |
| 34 | SESN3 | 3 | -1.86 | **5.3** | 9_BC6 |  |  |  |  |  |  |  |
| 35 | INHBB | 3 | -1.04 | **5.3** | 11_BC9 |  |  |  |  |  |  |  |
| 36 | SLC3A1 | 4 | -2.12 | **5.3** | 18_BC11 |  |  |  |  |  |  |  |
| 37 | NRIP2 | 4 | -1.10 | **5.1** | 8_BC7 |  |  |  |  |  |  |  |
| 38 | CYP2B6 | 4 | -5.41 | **5.1** | 14_BC6 |  |  |  |  |  |  |  |
| 39 | NR0B1 | 5 | -1.83 | **5.0** | 13_BC8 |  |  |  |  |  |  |  |
| 40 | TMPRSS2 | 5 | -3.81 | **5.0** | 14_BC9 |  |  |  |  |  |  |  |
| 41 | SLC7A9 | 5 | -1.94 | **5.0** | 14_BC2 |  |  |  |  |  |  |  |
| 42 | EPB41L3 | 3 | -3.56 | **4.9** | 19_BC11 |  |  |  |  |  |  |  |
| 43 | DKFZP564O0823/PARM1 | 4 | -2.45 | **4.8** | 9_BC3 |  |  |  |  |  |  |  |
| 44 | PAG1 | 5 | -1.35 | **4.7** | 12_BC2 |  |  |  |  |  |  |  |
