# Supplementary material for: A functional genomic screen in vivo identifies CEACAM5 as a clinically relevant driver of breast cancer metastasis
Source: NPJ Breast Cancer. 2018 Apr 30;4:9. doi: 10.1038/s41523-018-0062-x (PMC5928229; doi:10.1038/s41523-018-0062-x)
Supplement: Supplementary file 5 — Supplementary Table 4 [file 41523_2018_62_MOESM5_ESM.docx]

Supplementary Table 4

| Barcode | Barcode Seq | SYBR qPCR Primer |
| --- | --- | --- |
| 1 | TATTATGAGAAAGTTGAATAGTAG | GGTATTATGAGAAAGTTGAATAGTAGCCCT |
| 2 | ATGAAAGTTAGAGTTTATGATAAG | GGATGAAAGTTAGAGTTTATGATAAGCCCT |
| 3 | AATAGATAAGATTGATTGTGTTTG | GGAATAGATAAGATTGATTGTGTTTGCCCT |
| 5 | ATAGATTTAAGTGAAGAGAGTTAT | GGATAGATTTAAGTGAAGAGAGTTATCCCT |
| 6 | GAATGTTTGTAAATGTATAGATAG | GGGAATGTTTGTAAATGTATAGATAGCCCT |
| 7 | AAATTGTGAAAGATTGTTTGTGTA | GGAAATTGTGAAAGATTGTTTGTGTACCCT |
| 8 | TGTAAGTGAAATAGTGAGTTATTT | GGTGTAAGTGAAATAGTGAGTTATTTCCCT |
| 10 | AGTGAGATTATGAGTATTGATTTA | GGAGTGAGATTATGAGTATTGATTTACCCT |
| 11 | GTGTAAATGTTTGAGATGTATATT | GGGTGTAAATGTTTGAGATGTATATTCCCT |
| 17 | TTGTGAATGATTAATGAATAGAAG | GGTTGTGAATGATTAATGAATAGAAGCCCT |
| 19 | GTGTGTTATTTGTTTGTAAAGTAT | GGGTGTGTTATTTGTTTGTAAAGTATCCCT |
| 20 | AAATTAGTTGAAAGTATGAGAAAG | GGAAATTAGTTGAAAGTATGAGAAAGCCCT |
| 23 | TAGTGTAGATATTTGATAGTTATG | GGTAGTGTAGATATTTGATAGTTATGCCCT |
| 27 | AAGATGATAGTTAAGTGTAAGTTA | GGAAGATGATAGTTAAGTGTAAGTTACCCT |
| 28 | GATAGATTTAGAATGAATTAAGTG | GGGATAGATTTAGAATGAATTAAGTGCCCT |
| 29 | TTTAAGTGAGTTATAGAAGTAGTA | GGTTTAAGTGAGTTATAGAAGTAGTACCCT |
| 32 | AAGTGTTTAATGTAAGAGAATGAA | GGAAGTGTTTAATGTAAGAGAATGAACCCT |
| 33 | TATTAGAGTTTGAGAATAAGTAGT | GGTATTAGAGTTTGAGAATAAGTAGTCCCT |
| 34 | TGATATAGTAGTGAAGAAATAAGT | GGTGATATAGTAGTGAAGAAATAAGTCCCT |
| 35 | AATAAGAGAATTGATATGAAGATG | GGAATAAGAGAATTGATATGAAGATGCCCT |
| 37 | TGTATATGTTAATGAGATGTTGTA | GGTGTATATGTTAATGAGATGTTGTACCCT |
| 38 | AGTAAGTGTTAGATAGTATTGAAT | GGAGTAAGTGTTAGATAGTATTGAATCCCT |
| 39 | TTGTGATAGTAGTTAGATATTTGT | GGTTGTGATAGTAGTTAGATATTTGTCCCT |
| 40 | AATGTAGAGATTGTAGTGAATATT | GGAATGTAGAGATTGTAGTGAATATTCCCT |
| 43 | AAATAAGAATAGAGAGAGAAAGTT | GGAAATAAGAATAGAGAGAGAAAGTTCCCT |
| 44 | AATGTAAAGTAAAGAAAGTGATGA | GGAATGTAAAGTAAAGAAAGTGATGACCCT |
| 45 | GTTAGTTATGATGAATATTGTGTA | GGGTTAGTTATGATGAATATTGTGTACCCT |
| 46 | GTGATTGAATAGTAGATTGTTTAA | GGGTGATTGAATAGTAGATTGTTTAACCCT |
| 47 | TATTGTTGAATGTGTTTAAAGAGA | GGTATTGTTGAATGTGTTTAAAGAGACCCT |
| 48 | TATGAATGTTATTGTGTGTTGATT | GGTATGAATGTTATTGTGTGTTGATTCCCT |
| 50 | AGAGATATTAGAATGTAAGAATAG | GGAGAGATATTAGAATGTAAGAATAGCCCT |
| 52 | GTAAGATTAGAAGTTAATGAAGAA | GGGTAAGATTAGAAGTTAATGAAGAACCCT |
| 53 | GTTTGTGTTTGTATAAGTTGTTAA | GGGTTTGTGTTTGTATAAGTTGTTAACCCT |
| 54 | TAGAGAAAGAGAGAATTGTATTAA | GGTAGAGAAAGAGAGAATTGTATTAACCCT |
| 56 | AATTAGAAGTAAGTAGAGTTTAAG | GGAATTAGAAGTAAGTAGAGTTTAAGCCCT |
| 57 | AGAGTATTAGTAGTTATTGTAAGT | GGAGAGTATTAGTAGTTATTGTAAGTCCCT |
| 58 | TGAGAATGTAAAGAATGTTTATTG | GGTGAGAATGTAAAGAATGTTTATTGCCCT |
| 59 | ATTTAAGTAAAGTGTAGAGATAAG | GGATTTAAGTAAAGTGTAGAGATAAGCCCT |
| 61 | TATTAGAGAGAAATTGTAGAGATT | GGTATTAGAGAGAAATTGTAGAGATTCCCT |
| 63 | TTTGTTGTTAAGTATGTGATTTAG | GGTTTGTTGTTAAGTATGTGATTTAGCCCT |
| 64 | ATGATGTGTTTGATTTGAATTGAA | GGATGATGTGTTTGATTTGAATTGAACCCT |
| 65 | TGAGTAAGTTTGTATGTTTAAGTA | GGTGAGTAAGTTTGTATGTTTAAGTACCCT |
| 68 | TATTTGATAAGAGAATGAAGAAGT | GGTATTTGATAAGAGAATGAAGAAGTCCCT |
| 69 | ATGAAAGATTTAGTTGTGAGATAT | GGATGAAAGATTTAGTTGTGAGATATCCCT |
| 70 | GATTAGTATTTAGTAGTAATAGAG | GGGATTAGTATTTAGTAGTAATAGAGCCCT |
| 72 | AATTGAGAAAGAGATAAATGATAG | GGAATTGAGAAAGAGATAAATGATAGCCCT |
| 73 | GTTGAGAATTAGAATTTGATAAAG | GGGTTGAGAATTAGAATTTGATAAAGCCCT |
| 74 | AATGAAATAGTGTTAAATGAGTGT | GGAATGAAATAGTGTTAAATGAGTGTCCCT |
| 75 | TTTGTTAGAATGAGAAGATTTATG | GGTTTGTTAGAATGAGAAGATTTATGCCCT |
| 76 | AAAGAATTAGTATGATAGATGAGA | GGAAAGAATTAGTATGATAGATGAGACCCT |
| 77 | GTTATGATATAGTGAGTTGTTATT | GGGTTATGATATAGTGAGTTGTTATTCCCT |
| 78 | GTATAGTGTGATTAGATTTGTAAA | GGGTATAGTGTGATTAGATTTGTAAACCCT |
| 79 | TAGTAGAATTGTTGTTAAAGAATG | GGTAGTAGAATTGTTGTTAAAGAATGCCCT |
| 80 | GATGAATATAGTAAGTATTGAGTA | GGGATGAATATAGTAAGTATTGAGTACCCT |
| 84 | TGAATTGAATAAGAATTTGTTGTG | GGTGAATTGAATAAGAATTTGTTGTGCCCT |
| 85 | GTTATGAAAGAGTATGTGTTAAAT | GGGTTATGAAAGAGTATGTGTTAAATCCCT |
| 90 | TAGATTAGTTGATAAGTGTGTAAT | GGTAGATTAGTTGATAAGTGTGTAATCCCT |
| 98 | AAATGTGTGTTTAGTAGTTGTAAA | GGAAATGTGTGTTTAGTAGTTGTAAACCCT |
| 99 | GTTAGAATGTATATAGAGTTAGAT | GGGTTAGAATGTATATAGAGTTAGATCCCT |
| 100 | TTAAGAAGAATTGTATATGAGAGT | GGTTAAGAAGAATTGTATATGAGAGTCCCT |
| 101 | TTTAGAGTTTGATTAGTATGTTTG | GGTTTAGAGTTTGATTAGTATGTTTGCCCT |
| 103 | GATGTATAATGATGTGTGTAAATT | GGGATGTATAATGATGTGTGTAAATTCCCT |
| 104 | TAAAGAGTGATGTAAATAGAAGTT | GGTAAAGAGTGATGTAAATAGAAGTTCCCT |
| 105 | TGTAGTGTTTAGAGTAAGTTATTA | GGTGTAGTGTTTAGAGTAAGTTATTACCCT |
| 106 | GTAATAAGTTGTGAAAGAAGATTA | GGGTAATAAGTTGTGAAAGAAGATTACCCT |
| 109 | TTTATGTGTGATTGAGTGTTTAAT | GGTTTATGTGTGATTGAGTGTTTAATCCCT |
| 110 | TATTTAGTTAGATAGATAGAGAGT | GGTATTTAGTTAGATAGATAGAGAGTCCCT |
| 111 | ATGTGTTTATGTGAAAGATTTGTA | GGATGTGTTTATGTGAAAGATTTGTACCCT |
| 116 | AGTTATAAGTAAATGATGTTGATG | GGAGTTATAAGTAAATGATGTTGATGCCCT |
| 117 | TTGTATGTGAGTTTAGATTAATGA | GGTTGTATGTGAGTTTAGATTAATGACCCT |
| 120 | TATAAGAAGTAATTTGAGAAGAGT | GGTATAAGAAGTAATTTGAGAAGAGTCCCT |
| 121 | TTTGATTTATGTGTTATGTTGAGT | GGTTTGATTTATGTGTTATGTTGAGTCCCT |
| 122 | AGATTGAAATAGATTAGAAAGTTG | GGAGATTGAAATAGATTAGAAAGTTGCCCT |
| 123 | GTTGTTATAAGAAATAGTTTGTTG | GGGTTGTTATAAGAAATAGTTTGTTGCCCT |
| 124 | AAGAAGTAAGAGAGAAATTTGAAT | GGAAGAAGTAAGAGAGAAATTTGAATCCCT |
| 125 | AGATAAGTTAAAGTAAAGAGAATG | GGAGATAAGTTAAAGTAAAGAGAATGCCCT |
| 126 | TAGTTGAAGTTAGTTTAAGTGTTA | GGTAGTTGAAGTTAGTTTAAGTGTTACCCT |
| 127 | AGTAAGAATGTAATATGATGATAG | GGAGTAAGAATGTAATATGATGATAGCCCT |
| 129 | ATTGTGTTTAAGAAATATGATGAG | GGATTGTGTTTAAGAAATATGATGAGCCCT |
| 130 | ATTTGTGTGATGTTTGAAATATGA | GGATTTGTGTGATGTTTGAAATATGACCCT |
| 131 | AGAGAAATTGAATTTAGAAATGTG | GGAGAGAAATTGAATTTAGAAATGTGCCCT |
| 132 | TTAGAAGATAGATTATTGAGAAAG | GGTTAGAAGATAGATTATTGAGAAAGCCCT |
| 133 | AGTAAGAAAGTTTAGTTTAGTTAG | GGAGTAAGAAAGTTTAGTTTAGTTAGCCCT |
| 135 | TTAAAGATGTTAAAGAATGAGTGA | GGTTAAAGATGTTAAAGAATGAGTGACCCT |
| 136 | TGTAGTATGAAGAATAATGAAATG | GGTGTAGTATGAAGAATAATGAAATGCCCT |
| 137 | ATTTAGAGTTGTAAGAAGATATTG | GGATTTAGAGTTGTAAGAAGATATTGCCCT |
| 138 | GAGAAATTGTAATTGTTAGAGTAT | GGGAGAAATTGTAATTGTTAGAGTATCCCT |
| 139 | AATATTGAAGATGTAGTGAGTTAT | GGAATATTGAAGATGTAGTGAGTTATCCCT |
| 141 | TGAGTAAATAGTTTATGAGTAGTA | GGTGAGTAAATAGTTTATGAGTAGTACCCT |
| 142 | TTTAGTTTGATGTGTTTATGAGAT | GGTTTAGTTTGATGTGTTTATGAGATCCCT |
| 144 | TTAAGTGAAGTGTTGTTTATTGAA | GGTTAAGTGAAGTGTTGTTTATTGAACCCT |
| 147 | TGATTTAGTATGTATTAGAGTTGA | GGTGATTTAGTATGTATTAGAGTTGACCCT |
| 148 | ATTTATGTAGTTGAGAGTGATAAA | GGATTTATGTAGTTGAGAGTGATAAACCCT |
| 149 | GTAAAGATAGTTTGAGTAATTTGA | GGGTAAAGATAGTTTGAGTAATTTGACCCT |
