# Supplementary material for: A functional genomic screen in vivo identifies CEACAM5 as a clinically relevant driver of breast cancer metastasis
Source: NPJ Breast Cancer. 2018 Apr 30;4:9. doi: 10.1038/s41523-018-0062-x (PMC5928229; doi:10.1038/s41523-018-0062-x)
Supplement: Supplementary file 6 — Supplementary methods [file 41523_2018_62_MOESM6_ESM.docx]

**Supplemental Methods**

**PCR method to assess presence of human stromal fibroblasts**

To assess whether the co-implanted human EG fibroblasts, which stably express GFP, were cleared after tumor formation, gDNA was extracted from snap-frozen tumor fragments. Primers recognizing the gene encoding GFP and 50 ng of template gDNA were used in a PCR reaction with TEMPase 2x hot start polymerase (Apex) according to the manufacturer’s specifications. Amplicons were visualized by agarose gel electrophoresis and ethidium bromide staining to detect GFP amplicons. PCR using primers recognizing the gene encoding GAPDH was used as a positive control to ensure the quantity and quality of DNA was sufficient for PCR analysis. Primer pairs for GFP were: Fwd 5’-AAGTTCATCTGCACCACCG; Rev 5’-TCCTTGAAGAAGATGGTGCG. Primer pairs for GFP were: Fwd 5’-ACATCATCCCTGCCTCTAC; Rev 5’-TCAAAGGTGGAGGAGTGG.

**FACS analysis to assess the presence of stromal fibroblasts**

BC3_A2 cells were implanted into MFPs of NOD/SCID mice as described above, and tumors were harvested when they reached approximately 1 cm in diameter (approximately 10 weeks after engraftment). Tumors were digested to single cells and organoids in collagenase and hyaluronidase as described above and subjected to FACS to quantify representation of GFP-positive EG fibroblasts.

**Bioluminescence Imaging**

Bioluminescence imaging (BLI) was performed as previously described (28). Briefly, animals were administered an intraperitoneal (i.p.) injection of 150 μg/g body weight D-luciferin (GoldBio, St. Louis, MO, USA) in phosphate-buffered saline (PBS). Ten minutes after injection, isoflurane-anesthetized animals were imaged with a charge-coupled device camera-based BLI system (IVIS Lumina and IVIS Spectrum; PerkinElmer, Waltham, MA, USA). Signals were displayed as photons/s/cm^2^/sr for image representation. Living Image Software was used to manually define regions of interest, and quantified data were expressed as total photon flux (photons/second). The first images were taken 1–2 weeks after orthotopic tumor implantation to MFPs, or immediately after tail vein injection of labeled cells, and weekly thereafter. MFP tumors were covered to block signal from the primary site and allow visualization of metastases to the axillary lymph node. To quantify organ distribution, D-luciferin was administered to live animals, and tissues were assessed with BLI *ex vivo* at necropsy.

**Cell culture and lentiviral transduction**

BC3_A2 cells were grown in DMEM supplemented with 10% FBS at 5% CO_2_ and 5% O_2_ in humidified air. For EMT assays, TGF-β was purchased from R&D Systems (40-B-002). BC3 cells were transduced with lentivirus following a modified published protocol (28). 293T cells were transfected using LT1 (Mirus) transfection reagent in 6-well plates with one target plasmid per well along with packaging vectors VSVG and pDELTA8.9. Virus was removed from cells 4 days post-transfection and passed slowly through a 0.45 um filter. BC3_A2 cells were immediately added to virus-containing media in the presence of 1 μg/ml polybrene. Cells were transduced at an approximate multiplicity of infection (MOI) of 3 so that one ORF was expressed at an average of one copy per cell. Single ORFs were expressed in PDX cells. One week later, transduced cells were trypsinized and counted using a fluoresecence-coupled automated cell counter (CellOMeter, Nexcelcom). For high throughput screening, GFP-positive cells were pooled in equal numbers (77,000 cells expressing an individual ORF (12 targets per pool) along with internal GFP control (total 13 ORFs per pool), and 1x10^6^ pooled cells were implanted into MFPs of 10 mice. A reference pellet was frozen on the day of implantation. A negative control group of 7 mice was included with tumors expressing pHAGE-GFP alone to assess the level of stochastic metastatic events in each staggered cohort. Thirteen weeks after engraftment, BLI was performed on MFP tumors and lung metastases at animal necropsy.

To silence CEACAM5 expression, lentivirus was produced and BC3_A2 cells were transduced as described above. Cells were selected in the presence of 1 ug/ml puromycin. shRNA sequences were as follows: shCEACAM5 #3: CCGGGCAGTATTCTTGGCGTATCAACTCGAGTTGATACGCCAAGAATACTGCTTTTTG; shCEACAM5 #5: CCGGGCACAGTATTCTTGGCTGATTCTCGAGAATCAGCCAAGAATACTGTGCTTTTTG. A non-specific shRNA (against firefly luciferase), shLuc, was used as a negative control.

Identity of cell lines was authenticated by STR profiling. All cell lines used in this study were confirmed mycoplasma negative.

**qPCR for *in vivo* screen**

Genomic DNA (gDNA) was used as a template for qPCR using SYBR Green AxuraQuant Probe Fast qPCR Mix LoRox (Azura Genomics, AZ-2801) according to the manufacturers instructions and using an annealing temperature of 61^o^C. For MFP tumors, 40 ng of template gDNA was used; when possible, 80 ng of template gDNA was used for lung metastases (40 ng was used when quantities were limiting). Primer pairs were as follows: WPRE (Lentiviral internal integration control) F: TCCTGGTTGCTGTCTCTTTATG, R: TGACAGGTGGTGGCAATG; Barcode amplification control F: ccaactttcttgtacaaagt, R: gccactgtgctggatatca. Barcode amplification control primers bind in the small region between the ORF and the barcode, approximately 10 base pairs upstream of the barcode sequence start site. For amplification of unique barcode regions, a unique forward primer (see Table S4) and the reverse primer from the barcode amplification control were used as a pair.

**Complexity test**

The open reading frame (ORF) for HOXA1 was spliced into the lentiviral plasmid DNA backbone pHAGE-GFP using gateway cloning, and 293T cells were transfected (along with a constant amount of lentiviral packaging vectors per well) in varying ratios of HOXA1:GFP as follows: 100% GFP, 3 ug pHAGE-GFP; 100% HOXA1, 3 ug pHAGE-HOXA1; 1:10, 0.3 ug pHAGE-HOXA1 + 2.7 ug pHAGE-GFP; 1:20, 0.15 ug pHAGE-HOXA1 + 2.85 ug pHAGE-GFP; 1:40, 0.075 ug pHAGE-HOXA1 + 2.925 ug pHAGE-GFP. Lentivirus was collected 48 and 72 hours post-transduction, and BC3_A2 cells were transduced in the presence of 0.01 mg/ml protamine sulfate at an approximate MOI of 3. One million cells were implanted into the fourth MFPs of NOD/SCID mice in the presence of activated fibroblasts as described above. Animals were euthanized 13 weeks after implantation, and metastasis to the lungs was assessed with BLI as described above.

**Nucleic acid extraction**

Tissues were homogenized using a Bullet Blender. gDNA was extracted using the DNeasy Blood & Tissue kit (Qiagen) according to the manufacturer’s specifications. Total RNA was isolated by Trizol-chloroform extraction followed by purification using the RNEASY kit as described above. RNA was DNase-treated as described above.

**Quantitative real-time polymerase chain reaction (qRT-PCR) for gene expression**

Each 40 μl RT reaction consisted of no more than 4 μg template RNA. RNA was reverse transcribed using the Superscript III system (Invitrogen). The resulting cDNA was used for qPCR using the TaqMan Gene Expression Assays (Applied Biosystems), and data were normalized to a multiplexed endogenous control, GAPDH. No-template and no-RT controls were run on each plate, and amplification was not observed for any samples. qPCR was performed on the ViiA 7 Real-Time PCR System (Life Technologies).

**Immunohistochemistry and Immunofluorescence**
Tissues were fixed in 10% neutral buffered formalin for 24- 48 hours. Samples were then washed for 10 mins three times with PBS and transferred to 70% ethanol and embedded in paraffin. Five micron sections were cut and heated at 65^o^C for 1 hour. Staining was performed as previously described (28). Antibodies were diluted as follows: 1:50 for vimentin (University of Iowa Developmental Studies Hybridoma Bank, AMF-17B); 1:800 for CEACAM5 (Abcam ab131070); 1:100 for p-p38 (CST 4631); and 1:1500 for phospho- histone H3 (Sigma-Aldrich; H9908). For vimentin staining, the M.O.M Kit was used (Vector Laboratories MP-2400). For CEACAM5 and phospho-p38, the ImmPRESS Reagent Anti-Rabbit IgG kit was used (Vector Laboratories MP-7401). For pHH3, the ImmPRESS Reagent Anti-Rat IgG kit was used (Vector Laboratories MP-7404). Quantitation was performed using the Vectra 3.0 automated imaging system.

**Western blotting**

Samples were lysed in 1% Triton X-100, 50mM HEPES, pH 7.4, 150mM NaCl, 1.5mM MgCl_2_, 1mM EGTA, 100mM NaF, 10mM Na pyrophosphate, 1mM Na_3_VO_4_, 10% glycerol, with protease and phosphatase inhibitors. 10-30 ug total protein was loaded onto 4-20% polyacrylamide gels (Criterion, BioRad) and transferred to PVDF membranes for Western blotting using standard procedures. Antibodies used for Western blotting include those recognizing vimentin (Thermo Fisher MA5-11883), E-cadherin (BD 610181), CEACAM5 (Abcam ab131070), phospho-p38 (CST 9211S), total p38 (CST 9212), phospho-Smad2/3 (CST 8828), total Smad3 (Life Technologies 511500), GAPDH (Santa Cruz sc-365062), histone H3 (CST 4499), vinculin (Abcam ab129002), b-tubulin (CST 2128S), and Hsp90 (Santa Cruz, sc-7947). E-cadherin, CEACAM5, total p38, GAPDH, histone H3, vinculin, b-tubulin and Hsp90 were diluted 1:1000. Phospho-p38, phospho-smad2/3, and total Smad3 were diluted 1:200. Quantitation was performed using ImageJ. Blots derive from the same experiment and were processed in parallel.
