# Supplementary material for: A functional genomic screen in vivo identifies CEACAM5 as a clinically relevant driver of breast cancer metastasis
Source: NPJ Breast Cancer. 2018 Apr 30;4:9. doi: 10.1038/s41523-018-0062-x (PMC5928229; doi:10.1038/s41523-018-0062-x)
Supplement: Supplementary file 6 [file 41523_2018_62_MOESM4_ESM.docx]

Supplementary Table 3

Supplemental Table 2
Patient characteristics

Paired lung met and breast tumor	Age at tissue harvest	Race	Met Grade, differentiation status: 3=poor, 2=moderate, 1=well, NA=no info available	Met Size (cm)	Met ER: ?(POS)= positive without exact percent, ?(NEG)= negative without exact percent, NA=no info available	Met PR: ?(POS)= positive without exact percent, ?(NEG)= negative without exact percent, NA=no info available	Met HER2: HER2 score as 0,1+,2+,3+. ?(NEG)=negative without exact HER2 score, POS (FISH)=positive confirmed by FISH.	Primary Breast Cancer	Primary tumor Size (cm)	Primary Tumor Grade, differentiation status: 3=poor, 2=moderate, 1=well, NA=no info available	LN Met: shown as pos LN number/total LN number, NA= no data available	Stage T	Stage N	Stage M	ER: same with Met ER	PR: same with Met PR	HER2: same with Met HER2	Ki67 (%)	Note
	51	Unknown	NA	1.8	NA	NA	2+/POS (FISH)	IDC	2.3	3	1/10	4	3	0	0	0	3+/POS (FISH)	NA	

Paired tissues

TMA location	Age at tissue harvest	Race	Met Grade, differentiation: 3=poor, 2=moderate, 1=well, NA=no info available	Met Size (cm)	Met ER: ?(POS)= positive without exact percent, ?(NEG)= negative without exact percent, NA=no info available	Met PR: ?(POS)= positive without exact percent, ?(NEG)= negative without exact percent, NA=no info available	Met HER2: HER2 score as 0,1+,2+,3+. ?(NEG)=negative without exact HER2 score, POS (FISH)=positive confirmed by FISH.	Primary Breast Cancer	Primary tumor Size (cm)	Primary Tumor Grade, differentiation: 3=poor, 2=moderate, 1=well, NA=no info available	LN Met: shown as pos LN number/total LN number, NA= no data available	Stage T	Stage N	Stage M	ER: same with Met ER	PR: same with Met PR	HER2: same with Met HER2	Ki67 (%)	Note
E 10-12	56	White	3	1.8	? (NEG)	? (NEG)	NA	IDC	2.1	3	0/3	2	0	0	0	0	1+	70%	Brain metastasis on 2013
C 10-12	53	Black	2	0.6,0.5	? (POS)	? (NEG)	NEG (FISH)	Papillary DCIS	0.9	NA	0/22	1	0	0	? (POS)	? (POS)	NA	NA	
F 4-6	42	Asian	NA	2.0	>90%	0	1+/NEG (FISH)	IDC	1.9	2	0/19	1	0	0	60%	90%	0	35%	
A 10-12	85	White	3	2.5	100%	0	3+	IDC	2.0	3	4/20	2	3	0	15%	16%	3+	36%	
A 1-3	56	Hispanic	3	NA	<5%	0	1+	IDC	1.5	3	0/8	1	0	0	NA	NA	NA	NA	Brain met on 2009 (tissue available on this TMA)
E 7-9	49	Hispanic	3	1.5	NA	NA	NA	IDC	2.3	3	0/14	2	0	0	0	0	NEG (FISH)	90%	It was very hard to determine whether this lung adenocarcinoma is a primary lung or recurrence from her breast.
A 4-6	73	White	2	NA	? (POS)	? (NEG)	NA	Breast cancer	NA	NA	NA				NA	NA	NA	NA	
E 1-3	49	White	2	1.2	? (POS)	? (NEG)	NEG (FISH)	IDC	1.2	1	NA	1	0	0	100%	100%	NA	25%	
C 4-6	53	White	3	1.0	0	0	NA	IDC	2.2	3	0/11	2	0	0	0	0	0	NA	
B 7-9	74	White	Recurrent met	3.5	NA	NA	NA	Breast cancer	NA	NA	NA				NA	NA	NA	NA	Recurrent lung met from breast (first lung met tissue located on TMA1 A4-6)
A 7-9	43	White	NA	NA	? (POS)	? (NEG)	NA	IDC	2.0	3	7/10				? (POS)	? (POS)	0	30	With history of metastatic breast carcinoma to liver, lung, and bone
F 7-9	59	Black	3	1.8	0	0	0	IDC	2.5	3	0/7	2	0	0	<1%	0	90%	90%	
E 4-6	68	White	NA	1.5	0	0	NEG (FISH)	IDC	NA	NA	0/22	1	0	0	? (NEG)	? (NEG)	? (NEG)	NA	BRCA1 mutation 5385insC
C 1-3	50	Black	NA	1.8	60%	0	POS (FISH)	IDC	5	3	0/16	3	0	0	0	0	POS (FISH)	NA	
B 10-12	40	Hispanic	NA	2.8	? (NEG)	? (NEG)	NA	IDC	2.1	3	1/29	2	1	0	? (NEG)	? (POS)	0	NA	With history of metastatic breast carcinoma to lung, bone and brain
G 1-3	41	Asian	3	2.5	0	0	3+	IDC	1.0	3	1/8				0	0	3+	NA	Liver metastasis on 2013
D 7-9	58	Black	3	1.0	NA	NA	NA	Breast cancer	2.5	NA	10/7				? (NEG)	? (NEG)	? (POS)	NA	
D 1-3	64	White	3	NA	NA	NA	NA	IDC	5.0	3	1/8	2	1	0	? (NEG)	? (NEG)	? (NEG)	NA	
F 10-12	61	White	3	4.5	0	0	0	IDC	1.5	3	0/4				0	0	0/NEG (FISH)	80%	BRCA mutation. With a history of right breast cancer on 2004 (3.0cm, grade III, ER 100%, PR 15% and HER2 3+)
D 10-12	79	White	2	2.5	90%	NA	NEG (FISH)	IDC	0.7	3	0/3				95%	1%	NEG (FISH)	50%	
B 4-6	50	White	3	AND SKELETAl	NA	NA	NA	IDC	0.8	3	1/16				<5%	0	NEG (FISH)	NA	Brain met on 2002 (tissue available on this TMA)
C 7-9	49	White	3	4.9	0	NA	NEG (FISH)	IDC	2.5	3	NA	2	0	0	? (NEG)	? (NEG)	? (NEG)	NA	
D 4-6	60	White	3	NA	5%	0	POS (FISH)	IDC	1.5	3	0/15	4	0	0	0	0	POS (FISH)	NA	
B 1-3	66	White	3	6.0	? (NEG)	? (NEG)	? (NEG)	IDC	2.1	3	1/21	2	1	0	<5%	5%	0	NA	
F 1-3	65	White	NA	0.8	? (NEG)	? (NEG)	0	IDC	4.0	2	1/10	2	1	0	0	0	NEG (FISH)	NA	
